# Long-distance plant dispersal to North Atlantic islands: colonization routes and founder effect

**DOI:** 10.1093/aobpla/plv036

**Published:** 2015-05-14

**Authors:** Inger Greve Alsos, Dorothee Ehrich, Pernille Bronken Eidesen, Heidi Solstad, Kristine Bakke Westergaard, Peter Schönswetter, Andreas Tribsch, Siri Birkeland, Reidar Elven, Christian Brochmann

**Affiliations:** 1 Tromsø Museum, University of Tromsø, NO-9037 Tromsø, Norway; 2 Department of Arctic and Marine Biology, Faculty of Biosciences, Fisheries and Economics, University of Tromsø, NO-9037 Tromsø, Norway; 3 The University Centre in Svalbard, PO Box 156, NO-9171 Longyearbyen, Norway; 4 Museum of Natural History and Archaeology, Norwegian University of Science and Technology, NO-7491 Trondheim, Norway; 5 Norwegian Institute for Nature Research, PO Box 5685 Sluppen, NO-7485 Trondheim, Norway; 6 Institute of Botany, University of Innsbruck, Sternwartestraße 15, A-6020 Innsbruck, Austria; 7 Department of Organismic Biology, University of Salzburg, Hellbrunnerstraße 34, A-5020 Salzburg, Austria; 8 Centre for Ecological and Evolutionary Synthesis, Department of Biosciences, University of Oslo, PO Box 1066 Blindern, NO-0316 Oslo, Norway; 9 National Centre for Biosystematics, Natural History Museum, University of Oslo, PO Box 1172 Blindern, NO-0318 Oslo, Norway

**Keywords:** Amplified fragment length polymorphism (AFLP), dispersal vector, founder effect, genetic diversity, islands, long-distance dispersal (LDD), postglacial, species traits

## Abstract

Long-distance dispersal (LDD) processes influence the founder effect on islands. We use genetic data for 25 Atlantic species and similarities among regional floras to analyse colonization, and test whether the genetic founder effect on five islands is associated with dispersal distance, island size and species traits. Most species colonized postglacially via multiple dispersal events from several source regions situated 280 to >3000 km away, and often not from the closest ones. A strong founder effect was observed for insect-pollinated mixed maters, and it increased with dispersal distance and decreased with island size in accordance with the theory of island biogeography. Only a minor founder effect was observed for wind-pollinated outcrossing species. Colonization patterns were largely congruent, indicating that despite the importance of stochasticity, LDD is mainly determined by common factors, probably dispersal vectors. Our findings caution against a priori assuming a single, close source region in biogeographic analyses.

## Abstract (precis)

Our study provides new knowledge of two processes that are important for plant adaptation in a changing environment: 1) long-distance dispersal patterns, and 2) genetic founder effect on islands. Although the theoretical framework for the genetic founder effect on islands was proposed in 1973, we are the first to quantify it in relation to island size, dispersal distance, and plant traits. In addition, our genetic results are mainly coherent with post-glacial colonisation rather than *in situ* glacial survival, and should therefore bring a final end to the 140-year-long glacial survival-*tabula rasa* debate among northern biologists.

## Introduction

Long-distance dispersal (LDD) of plants is a complex process (Higgins *et al*. 2003). Direct observations of LDD are rare (Ridley 1930); therefore, it is usually inferred from the geographical distribution of species or genes (de Queiroz 2005). Effective LDD (also termed long-distance colonization) involves seed release, dispersal by one or several vectors, arrival in a favourable microhabitat, germination and successful establishment of a new population (Chambers and MacMahon 1994; Nathan 2006). Several factors may influence each of these components. Dispersal routes and frequencies may depend on historical factors such as past climate shifts and geographical distributions (Taberlet *et al*. 1998), as well as on dispersal vectors such as birds, sea currents and wind (Gillespie *et al*. 2012). Local establishment depends on the number of arriving propagules, adaptation of the newcomer to local ecological conditions, including abiotic factors and relationship to pollinators or mycorrhiza partners (Chambers and MacMahon 1994; Hegland *et al*. 2009). Because LDD of plants is rarely directly observed, quantifying its relationship to potential determining factors is challenging (Nathan 2006). The relative importance of deterministic versus stochastic processes in shaping LDD patterns is not clear (Higgins *et al*. 2003; Nathan 2006; Vargas *et al*. 2012).

Oceanic islands represent good models to study LDD, as every species (or its ancestor) on such islands must have arrived by LDD. According to the equilibrium theory of island biogeography, the number of species on an island increases with island size and decreases with distance to source regions (MacArthur and Wilson 1967), although other factors such as species traits, sea current, past and present climate, and habitat heterogeneity may also play a role (Triantis *et al*. 2012; Weigelt and Kreft 2013). Similarly, the amount of genetic diversity in island populations is expected to be positively correlated with island size, but typically to be lower than in continental populations as only a limited number of genotypes from the source populations are expected to disperse to the recipient region causing a genetic founder effects (Jaenike 1973; Frankham 1997). As the frequency of dispersal events decreases with distance (Nathan 2006; Nathan *et al*. 2008), the initial founder effect and restriction of subsequent immigration, both leading to genetic depauperation of island populations, may increase with distance to the source region (Bialozyt *et al*. 2006; Dlugosch and Parker 2008; Pauls *et al*. 2013). Genetic diversity in plant populations is not only a result of population history but also related to species traits such as pollination mode, breeding system, growth form and morphological adaptations to dispersal (Hamrick and Godt 1996; Thiel-Egenter *et al*. 2009), all factors that may affect the intensity of founder effects. If species diversity and genetic diversity on islands are shaped by the same deterministic colonization processes, relative levels of genetic diversity should be related to the levels of species diversity. Moreover, floristic and genetic similarities should point to the same source regions for island colonization.

The role of LDD in shaping the current northern flora, which contains species that typically are widely distributed across a naturally fragmented biome, is debated (Löve and Löve 1963; Brochmann *et al*. 2013). In the Arctic, efficient LDD may be frequent due to open landscapes, strong winds and numerous migrating birds, a prediction supported by genetic data for the isolated archipelago of Svalbard (Alsos *et al*. 2007). Sea ice may also facilitate dispersal, as a ‘bridge’ or as a rafting vector (Johansen and Hytteborn 2001). Nevertheless, floristic analyses have indicated that most Arctic islands are not saturated with species (Hoffmann 2012). Similarly, analyses of plant species diversity in the Arctic mainland indicate that species distributions are limited by dispersal and/or establishment conditions (Lenoir *et al*. 2012).

The potentially strongest barrier to plant dispersal in the circumpolar region is the North Atlantic Ocean. For more than 100 years it has been debated whether plants were able to cross it via LDD after the last glaciation, or whether they depended on surviving the last (or several) glaciation(s) in local ice-free refugia in different Atlantic regions (Löve and Löve 1963; Brochmann *et al*. 2003). Molecular evidence clearly shows that trans-Atlantic LDD has occurred recently in many species (Brochmann *et al*. 2003). The Atlantic Ocean (and the Greenlandic ice sheet) is nevertheless a stronger barrier against dispersal than continuous Arctic landmasses, as shown in a recent circumpolar analysis of genetic variation in 17 vascular plant species (Eidesen *et al*. 2013). Even though the current floras in various Atlantic regions mainly have established following postglacial colonization, genetic data for a few species indicate *in situ* glacial persistence (Westergaard *et al*. 2011; see also Parducci *et al*. 2012).

To gain a better understanding of the factors determining LDD, we here analysed genetic structure in 25 plant species in five islands and adjacent mainland regions in the North Atlantic, as well as similarities in species composition among regional floras. We ask whether genetic data (i) support the prevalent hypothesis of postglacial long-distance colonization or, alternatively, local glacial survival, (ii) determine the source areas for postglacial island colonization in the North Atlantic region, (iii) quantify the intensity of the genetic founder effect and investigate how it relates to distance, island size and plant species traits and (iv) compare genetic and floristic relationships among regions.

## Methods

### Geographical regions

We selected five recipient islands/archipelagos: East Greenland (182 440 km^2^ as delimited by Elven *et al*. 2011), Iceland (103 000 km^2^), Svalbard (24 453 km^2^ of non-glaciated area), the Faroe Islands (1396 km^2^) and Jan Mayen (377 km^2^). Although East Greenland is only part of an island, we treated it as an island because only narrow strips of land disrupted by glaciers connect it to North and South Greenland, and the Inland Ice Sheet forms a firm dispersal barrier to West Greenland (Eidesen *et al*. 2013). All surrounding land masses in north-eastern North America and Europe were selected as potential source regions. Minimum distances between recipient island and source regions (coast to coast) were estimated using Google Earth version 6.2.0.5905 (beta).

All recipient islands were mainly glaciated during the last glacial maximum (LGM, ∼20 000 cal. BP, Ehlers and Gibbard 2004) although minor ice-free areas existed (reviewed in Brochmann *et al*. 2003). Pollen and macrofossil studies show that a flora including many of the species we analysed for genetic variation existed on East Greenland from 12 800 to 12 300 cal. BP (Bennike 1999; Bennike *et al*. 1999), on Iceland from 13 000 to 10 800 cal. BP (Rundgren 1998; Rundgren and Ingólfsson 1999; Caseldine *et al*. 2003), on Svalbard from 9000 cal. BP (Birks 1991) and on the Faroe Islands from 13 100 cal. BP (Hannon *et al*. 2010). No late glacial or early Holocene palaeobotanical studies exist from Jan Mayen. Iceland, the Faroe Islands and Jan Mayen are true oceanic islands, whereas Svalbard and Greenland are continental islands. However, due to the previous heavy glaciation also of the latter two, they may be viewed as mainly oceanic islands in terms colonization processes.

### Genetic data

We assembled amplified fragment length polymorphism (AFLP) datasets for Arctic and north-boreal species of vascular plants present in the five recipient islands. Most data originate from published studies **[see Supporting Information—Table S1]**. We included only AFLP datasets of high quality, e.g. with error rates estimated from random replicates, test of many primers before selection of final primer set (see Alsos *et al*. 2007, 2012 for details) and based on extensive sampling in the North Atlantic area. Our final dataset comprised 25 species, 1110 local populations, 8932 individual plant samples and 3653 polymorphic markers **[see Supporting Information—Table S1]**. Details on the AFLP analyses of 24 of the 25 species have been published elsewhere [**see Supporting Information—Table S1**, for *Sibbaldia procumbens*].

### Species traits

We expected four species traits to be most important in determining the intensity of the genetic founder effect: mode of pollination (insect or wind), breeding system (outcrossing, selfing or mixed mating), growth form (woody or herbaceous) and dispersal adaptation (long-distance or short-distance). Dispersal adaptation was defined as ‘long-distance-dispersed’ if morphologically adapted to wind- or animal-dispersal, even though the regular dispersal distance in such species may be moderate rather than long (Higgins *et al*. 2003; Tamme *et al*. 2014); otherwise as ‘short-distance-dispersed’. Only 10 species in the North Atlantic region have adaptations for dispersal by sea current (Löve 1963); as none of them were analysed here, this category was not included. However, a large proportion of the species have seeds that might float (Thiel and Gutow 2005). Higher levels of genetic diversity are typically found in wind-pollinated, outcrossing and woody species than in insect-pollinated, selfing and herbaceous species (Hamrick and Godt 1996; Thiel-Egenter *et al*. 2009). Information on these traits for the 25 species in the genetic dataset was compiled from the literature, following the criteria outlined in Alsos *et al*. (2012); **[see Supporting Information—Table S1]**. The founder effect has been shown to be related to adaptation to local climate (Alsos *et al*. 2007), but the observed reduction in genetic diversity might be explained by a bottleneck due to cooler climate on Svalbard during the last 2000 years causing a decrease in distribution of, for example, *Betula nana* and *Salix herbaceae* (Birks 1991; Alsos *et al*. 2002). However, as most species are not at their climatic limit on most recipient islands at present (except some on Svalbard, Elven *et al*. 2011), we did not include that factor here.

### Genetic data analyses

**Figure 1.**
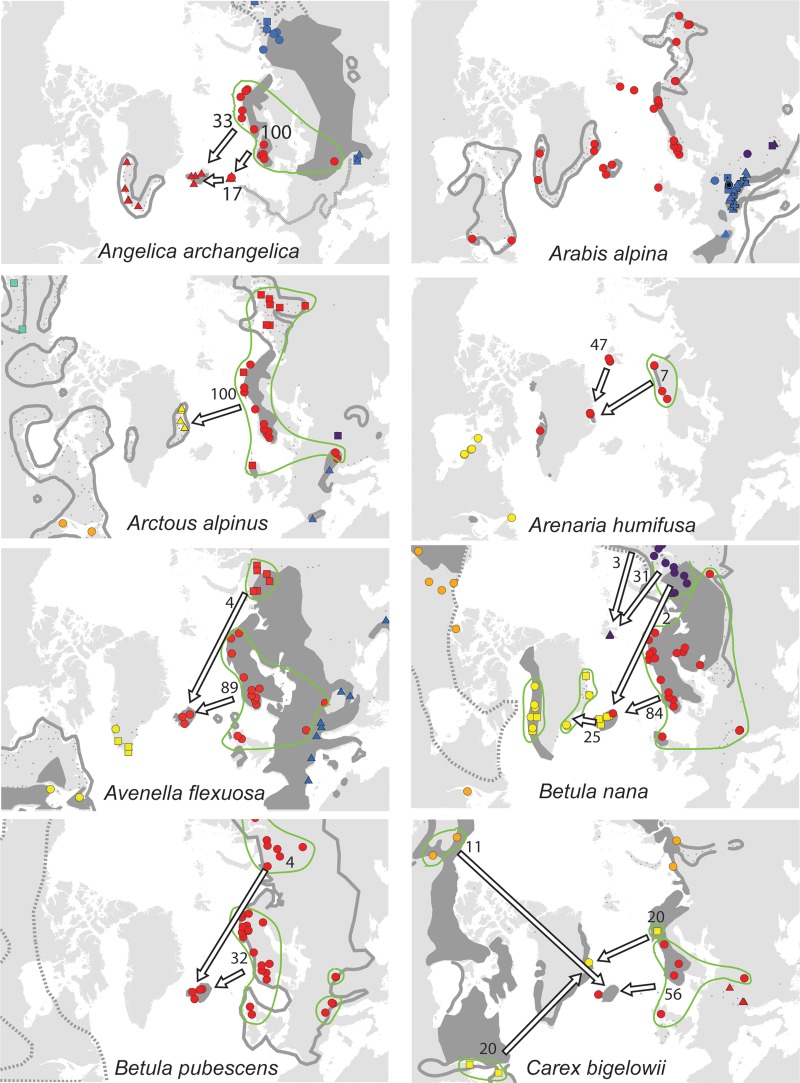

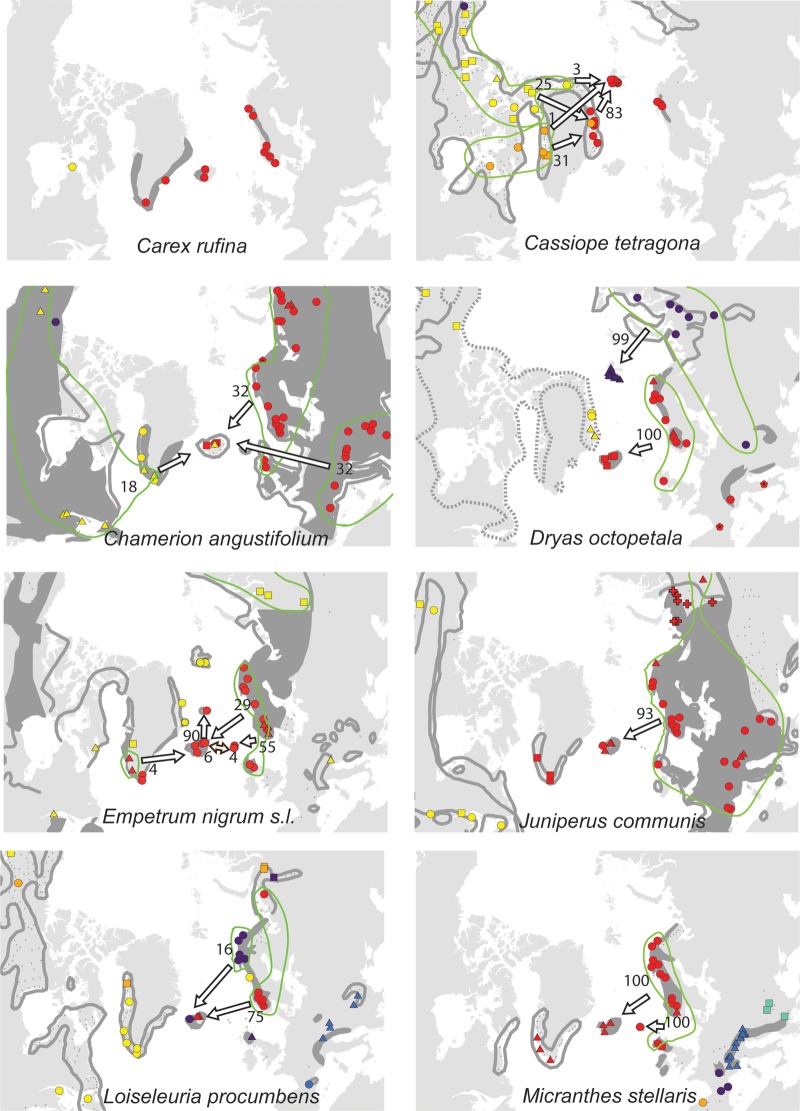

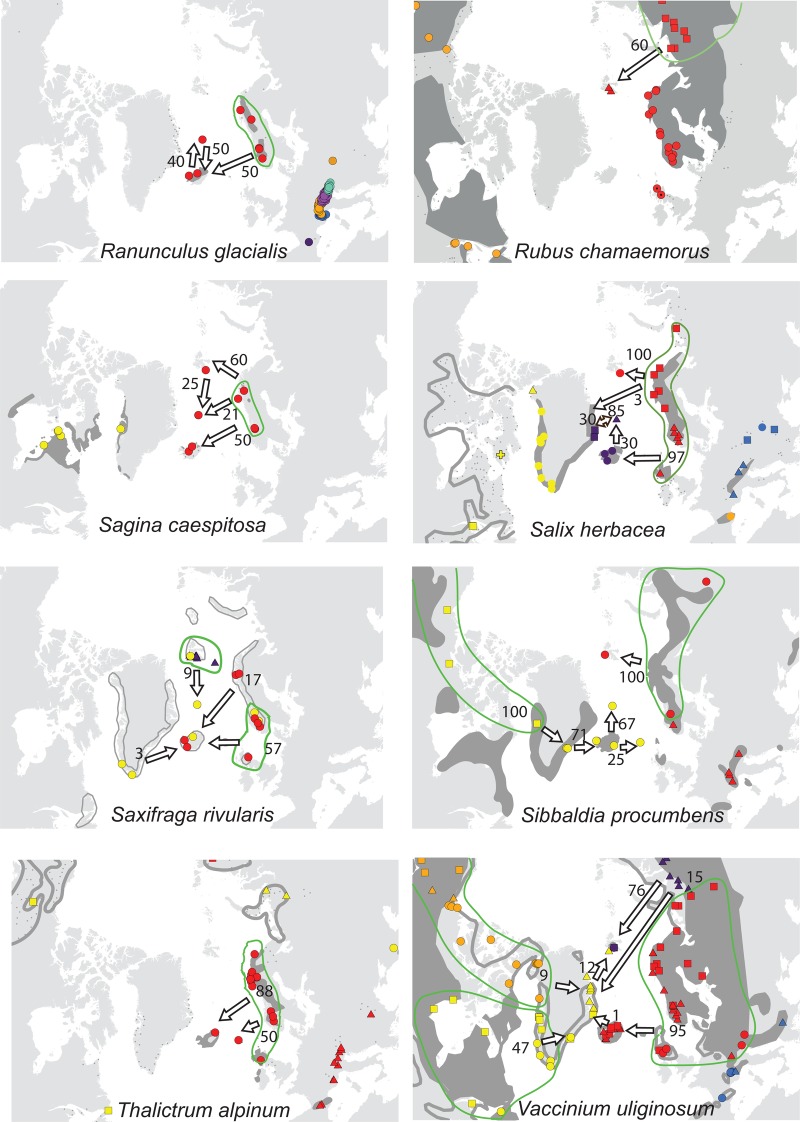

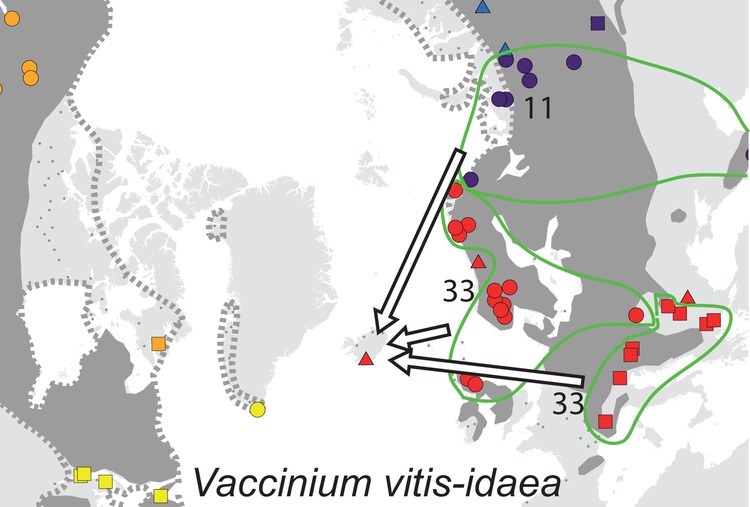
Maps showing the genetic structuring of the 25 species analysed for AFLPs. Colours identify the main genetic groups according to Bayesian clustering analyses run with STRUCTURE and other methods (see text); symbol shapes identify subgroups within main groups. The present distribution of the species is given according to Hultén and Fries (1986; dark grey area, dots and outline whereas stippled lines show vicariant taxa). Arrows represent dispersal routes inferred from assignment tests to geographical regions. Numbers show the proportion (%) of plants allocated to each source region. Due to lack of genetic variation, no assignment test was performed for *Arabis alpina* and *Carex rufina*. No assignment test was performed for *Dryas octopetala* in East Greenland as our sampling from that region only comprises assumed hybrids with *D. integrifolia* (Skrede *et al*. 2006). For *Micranthes stellaris*, Icelandic plants allocated to the combined regions Faroe Island, Scotland and Scandinavia, whereas Faroe Island plants allocated to the Scandinavian-Scottish subgroups (red dots on the map).

For each species, the sampled area was divided into regions according to geographically consistent genetic groups identified (cf. Alsos *et al*. 2007; Eidesen *et al*. 2013) **[see Supporting Information]**. The geographic distribution of the main genetic groups and subgroups for each species are shown in Fig. 1.

The source region for the populations on each of the five recipient islands was inferred by looking at shared genetic groups among regions and by carrying out a multilocus assignment test in AFLPOP (Duchesne and Bernatchez 2002). We used a log-likelihood difference of one as threshold for allocation (i.e. for a genotype to be assigned to one particular source region it should be 10 times as likely assigned to that source region than to any other source regions; [see also **Supporting Information**]). For each recipient island and each species, we calculated the proportion of individuals allocated to each source region according to the assignment test (excluding individuals that were not assigned with a log-likelihood difference of 1) resulting in 51 recipient islands × species combinations. The source region with the highest proportion of allocation was considered the main source region. As we are addressing historical colonization and not present day immigration, the results of the assignment test should not be interpreted as revealing individual immigrant individuals. Nevertheless, as the number of generations since colonization is small in evolutionary time for these mainly long-lived plant species (Alsos *et al*. 2002; de Witte and Stöcklin 2010), we are confident that our analyses reveal the main colonization directions despite possible drift in the founded population.

As in Alsos *et al*. (2007), we quantified the genetic founder effect using six different measures: (i) a minimum number of dispersed propagules that resulted in successful colonization (*propagules*; estimated as the smallest number of individual genotypes in the main source region necessary to bring all observed markers to the recipient island, [**see Supporting Information**]), (ii) proportion of intrapopulation genetic diversity observed in the recipient island relative to that in the main source region (*population diversity*; estimated as the mean of the population averages of number of pair-wise differences among individuals), (iii) proportion of regional (total) genetic diversity in the recipient island relative to that in the main source region (*regional diversity*; estimated across all individuals in the region), (iv) proportion of AFLP markers observed in the recipient island relative to those in the main source region (*markers*), (v) number of source regions inferred in order to account for all markers observed in the recipient island (*sources markers*) and (vi) number of source regions estimated from the assignment test (*sources allocation*). All six measures are also influenced by potential effective dispersal events occurring after initial colonization; in the following we therefore use the term ‘founder effect’ to encompass the overall reduction in genetic variability in a population in a colonized area as compared with its source. Correlations among the measures and differences in intensity of the founder effect among species and recipient islands were investigated using principle component analysis (PCA) as implemented in the R-package ade4 (http://cran.r-project.org/web/packages/ade4/) and R version 3.01 (R Core Team 2013).

We tested for correlations among the independent variables (species traits, island size and dispersal distance). As growth form and dispersal type were significantly correlated, we chose to use dispersal type because we assume it to be directly relevant to the founder effect (Alsos *et al*. 2012). We also found a significant correlation between pollination mode and breeding system **[see Supporting Information—Tables S1 and S2]**, and chose the predictor variable with fewest categories (pollination mode). To determine to what extent distance between source and recipient island, island size, dispersal and pollination mode were correlated with the intensity of the founder effect, we carried out a Principal Component Analyses with Instrumental Variable (PCAIV; function pcaiv in ade4, Thioulouse and Dray 2007). To further test for significant associations, we carried out a linear mixed model (LMM) analysis with regional diversity as a response variable. We chose regional diversity as an estimate of founder effect here because it was most correlated with the first axis in the PCA. Species was included as a random effect in all models. The explanatory variables included as fixed effects were distance, area, pollination mode and dispersal type. These variables were assembled into 17 candidate models comprising a constant response as the simplest model, each explanatory variable alone, as well as all possible combinations of two variables with or without interactions between them. Models were fitted using the function lmer in the R package lme4 (Bates *et al*. 2014). Maximum likelihood (ML) was used as an optimization criterion to fit models for model selection, whereas restricted ML (REML) was used to obtain parameter estimates (Pinheiro and Bates 2000). The best models were selected based on Akaike's Information Criterion corrected for small sample size (AICc, Burnham and Anderson 2007) using AICcmodavg (Mazerolle 2011) in R. Models with a difference in AICc of <2 were considered equally appropriate. The selected models were checked graphically for constant variance of the residuals, presence of outliers and approximate normality of the random effects.

The likelihood that a species immigrated to the recipient island postglacially (rather than survived the last glaciation *in situ*) was evaluated based on the amount of genetic diversity in the recipient island relative to that in potential source regions, as well as on the number of private markers (markers restricted to one geographical region and thus likely represent local mutations) found in the recipient island (Westergaard *et al*. 2011).

### Floristic data

To compile data on species occurrences, we used the Pan Arctic Flora checklist (Elven *et al*. 2011) for those of our regions that are included there, otherwise Hultén and Fries (1986), various regional sources and personal observations [see complete taxon list per region in **Supporting Information—Table S3**]. Taxa closely associated with human activity and agricultural lands, including pasture lands, were assumed to have been introduced by humans to a region (Elven *et al*. 2011; Wasowicz *et al*. 2013, Alsos *et al*. 2015) and therefore omitted. Since the occurrence of some taxa is uncertain **[see Supporting Information—Table S3]**, we calculated floristic similarities as the minimum and maximum proportion of recipient island species that also occurred in each potential source region, and used the mean proportion in further analyses.

The number of years between each successful colonization events was estimated as the time since first postglacial palaeorecord/(total number of species on the island × proportion of species assumed to colonize postglacially × average number of propagules per species). Although these numbers contain uncertainties, they provide a rough estimate useful for comparison with other islands. For Jan Mayen, where no palaeobotanical records existed, we assumed a time period of 12 800 years, similar to East Greenland (12 700) and Iceland (13 000).

## Results

### Genetic data

In most cases, we observed less genetic diversity in the recipient islands than in the source regions both at the population and regional levels and in terms of number of markers, reflecting a founder effect (Table 1). We observed only few private markers in the recipient islands. *Sagina caespitosa* had relatively high numbers of private markers (5) in a recipient island, but this was not combined with high levels of genetic diversity, and thus not interpreted as indicating *in situ* glacial survival. Only *Arenaria humifusa* and *Saxifraga rivularis* showed a genetic pattern consistent with glacial survival on Svalbard **[see Supporting Information]**. Thus, 92 % of the species analysed were assumed to have colonized postglacially.

**Table 1.** Dispersal distance, number of private markers and six measures of the genetic founder effect for each species in each target island. The measures of founder effect are (i) minimum number of colonizing propagules (*Propagules*), (ii) proportion of intrapopulation genetic diversity in target relative to source (*Population diversity*), (iii) proportion of total genetic diversity in target relative to source (*Regional diversity*), (iv) proportion of AFLP markers in target relative to source (*Markers*), (v) number of source regions inferred from AFLP markers (*Sources markers*) and (vi) number of source regions inferred from assignment tests (*Sources allocation*). Target and main source regions are the Faroe Islands (FAROE), Iceland (ICE), East Canada (ECAN), East Greenland (EGRE), Jan Mayen (JM), North Canada (NCAN), mainland Norway (NOR), Northwest Europe (NWEUR), Russia (RUS), Southwest Greenland (SWGRE) and Svalbard (SVALB). Mean ± standard deviation values for each target island and overall mean are given.

Species	Target island	Main source region	Distance (km)	Number of private markers	Founder effect					
					Propagules	Population diversity	Regional diversity	Markers	Sources markers	Sources allocation
*Angelica archangelica*	FAROE	NWEUR	570	1	9	0.82	0.79	0.76	2	1
*Angelica archangelica*	ICE	NWEUR	775	1	8	0.82	0.78	0.81	2	2
*Arctous alpinus*	EGRE	NWEUR	1270	1	13	0.86	0.76	0.57	3	1
*Arenaria humifusa*	EGRE	SVALB	570	1	4	1.00	0.82	0.94	2	2
*Avenella flexuosa*	ICE	NWEUR	775	1	11	0.96	0.94	0.79	2	2
*Betula nana*	EGRE	ICE	280	0	13	0.73	0.94	0.82	3	1
*Betula nana*	ICE	NWEUR	775	1	14	1.11	1.00	0.81	3	4
*Betula nana*	SVALB	RUS	1000	1	7	0.68	0.70	0.59	2	3
*Betula pubescens*	ICE	NWEUR	775	2	12	1.10	1.06	0.77	2	2
*Carex bigelowii*	EGRE	ECAN	880	1	12	1.08	0.84	0.75	3	2
*Carex bigelowii*	ICE	NWEUR	775	3	16	1.58	1.16	0.76	3	2
*Cassiope tetragona*	EGRE	WGRE	360	1	14	1.00	1.06	1.04	4	2
*Cassiope tetragona*	SVALB	EGRE	570	1	11	0.94	0.97	0.91	3	3
*Chamerion angustifolium*	ICE	NWEUR	775	0	14	0.29	1.02	0.66	2	2
*Dryas octopetala*	ICE	NWEUR	775	0	5	0.63	0.54	0.55	2	1
*Dryas octopetala*	SVALB	RUS	1000	0	22	0.72	0.86	0.81	4	1
*Empetrum nigrum*	FAROE	NWEUR	285	0	3	0.72	0.58	0.53	1	2
*Empetrum nigrum*	ICE	NWEUR	775	0	6	0.75	0.65	0.63	1	3
*Empetrum nigrum*	JM	ICE	555	0	3	0.30	0.20	0.64	1	1
*Juniperus communis*	ICE	NWEUR	775	1	11	0.93	0.81	0.73	2	1
*Loiseleuria procumbens*	ICE	NWEUR	775	1	9	0.93	0.97	0.93	3	2
*Micranthes stellaris*	FAROE	NWEUR	285	0	1	1.38	1.05	0.71	1	1
*Micranthes stellaris*	ICE	NWEUR	775	3	2	1.12	0.79	0.71	1	1
*Ranunculus glacialis*	ICE	NWEUR	775	0	4	0.00	1.20	1.03	3	2
*Ranunculus glacialis*	JM	ICE	555	0	1	1.00	0.14	0.86	1	1
*Rubus chamaemorus*	SVALB	RUS	1000	0	5	0.47	0.51	0.62	1	1
*Sagina caespitosa*	ICE	NWEUR	965	5	9	0.65	0.81	0.76	2	1
*Sagina caespitosa*	JM	SVALB	875	1	11	0.35	0.30	0.68	2	2
*Sagina caespitosa*	SVALB	NOR	640	1	3	0.26	0.22	0.64	2	1
*Salix herbacea*	EGRE	JM	450	0	14	0.73	0.85	1.03	3	2
*Salix herbacea*	ICE	NWEUR	775	1	16	0.74	0.67	0.64	5	3
*Salix herbacea*	JM	ICE	555	0	13	0.97	0.92	0.79	3	2
*Salix herbacea*	SVALB	NWEUR	1000	1	12	0.67	0.70	0.57	3	1
*Saxifraga rivularis*	ICE	NWEUR	775	0	7	1.09	0.83	0.81	2	3
*Saxifraga rivularis*	JM	SVALB	945	0	4	0.26	0.21	0.55	1	1
*Sibbaldia procumbens*	EGRE	ECAN	360	0	5	0.77	0.69	0.80	2	2
*Sibbaldia procumbens*	FAROE	ICE	425	0	3	0.91	0.75	0.82	2	1
*Sibbaldia procumbens*	ICE	EGRE	280	1	5	0.55	0.70	0.98	2	1
*Sibbaldia procumbens*	JM	ICE	555	0	2	0.55	0.46	0.77	1	1
*Sibbaldia procumbens*	SVALB	NWEUR	640	0	2	0.00	0.00	0.75	1	1
*Thalictrum alpinum*	FAROE	NWEUR	285	0	14	1.12	0.94	0.68	3	1
*Thalictrum alpinum*	ICE	NWEUR	775	0	12	1.18	0.99	0.68	2	1
*Vaccinium uliginosum*	EGRE	WGRE	360	0	10	1.15	1.12	1.10	3	4
*Vaccinium uliginosum*	ICE	NWEUR	775	0	11	1.05	0.90	0.75	2	3
*Vaccinium uliginosum*	SVALB	RUS	1000	0	9	0.33	0.84	0.67	2	2
*Vaccinium vitis-idaea*	ICE	NWEUR	775	0	6	1.07	0.89	0.73	2	3
East Greenland			566 ± 342	0.5 ± 0.5	11.0 ± 4.0	0.92 ± 0.16	0.89 ± 0.15	0.88 ± 0.18	2.9 ± 0.6	2.0 ± 0.9
Iceland			758 ± 124	1.1 ± 1.4	9.4 ± 4.1	0.88 ± 0.35	0.88 ± 0.17	0.76 ± 0.12	2.3 ± 0.9	2.1 ± 0.9
Svalbard			856 ± 200	0.5 ± 0.5	8.9 ± 6.4	0.51 ± 0.03	0.60 ± 0.34	0.70 ± 0.12	2.3 ± 1.0	1.6 ± 0.9
Faroe Islands			370 ± 127	0.2 ± 0.6	6.0 ± 5.4	0.99 ± 0.26	0.82 ± 0.18	0.70 ± 0.11	1.8 ± 0.8	1.0 ± 0.0
Jan Mayen			673 ± 185	0.2 ± 0.4	5.7 ± 5.1	0.57 ± 0.34	0.37 ± 0.29	0.72 ± 0.11	1.5 ± 0.8	1.3 ± 0.5
Overall mean			693 ± 233	0.7 ± 1.0	8.6 ± 4.9	0.79 ± 0.34	0.76 ± 0.28	0.76 ± 0.14	2.2 ± 0.9	1.7 ± 0.8

**Figure 2.**
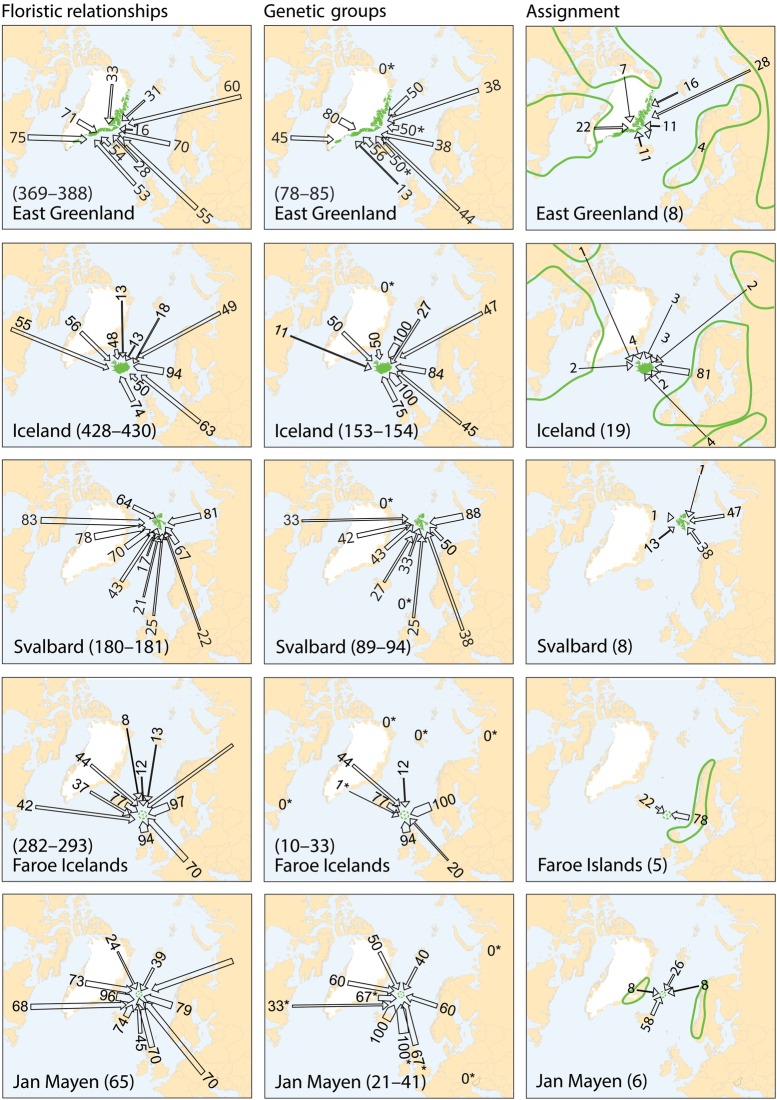
Floristic and genetic relationships between the five recipient islands (East Greenland, Iceland, Svalbard, the Faroe Islands and Jan Mayen), and potential source regions. Floristic relationships are expressed as the proportion (%) of all species occurring in each recipient island that also occur in each potential source region. Genetic relationships are expressed in two ways. First, as the proportions of genetic main groups that also are found in the source region (only counting source regions where populations have been analysed, stars denote regions where less than five comparisons were possible across species). Second, as the mean proportion (%) of plant individuals that were allocated to each source region in assignment tests (excluding individuals that were not assigned). Source regions for floristic and genetic group comparisons are as defined in **Supporting Information—Table S3**. Approximate delimitations of source regions of the assignment test are summarized across species (Fig. 1) and encircled in green. Number of species found (floristic data), genetic groups found (upper range is total number of genetic groups, lower range is number of genetic groups where observations for minimum five species are available) or species assigned (assignment data) in each recipient island are given in parentheses.

Of the 12 species analysed from East Greenland, the populations of five species belonged to amphi-Atlantic genetic groups, three to West-Atlantic groups, three to Greenlandic-Icelandic groups, *Arctous alpina* had unique groups and *Cassiope tetragona* had both a western and an eastern genetic group (Fig. 1). Overall, the highest proportions of genetic groups were shared with West Greenland (80 %, Fig. 2).

Among the 21 species analysed from Iceland, the populations of the majority of species belong to eastern (11) or amphi-Atlantic/East-Atlantic (7) genetic groups. Both eastern and western genetic groups were observed in *Betula nana* and *Chamerion angustifolium* (Fig. 1). All genetic groups were shared with Jan Mayen and Faroe Islands, whereas 75 and 84 % also were found in Great Britain and Norway, respectively (Fig. 2).

Among the 11 species analysed from Svalbard, the populations of five species belonged to amphi-Atlantic genetic groups (*Saxifraga rivularis* having a unique group in addition), four to East-Atlantic groups, one to West-Atlantic groups and *Vaccinium uliginosum* had both a western and an eastern genetic group (Fig. 1). The highest proportion of genetic groups was shared with Ural (88 %) followed by Norway (50 %) and East and West Greenland (42–43 %, Fig. 2).

In four of the five species analysed from the Faroe Islands, the populations belong to main genetic groups that were found both east and west of the archipelago although sometimes with different subgroups. Only *Sibbaldia procumbens* belong to a strict western genetic group. The high percentage of genetic groups shared among the regions is an effect of this but should be interpreted with caution due to the general low sample sizes.

All six species analysed from Jan Mayen fell into genetic groups together with individuals from Iceland. The populations of two species belonged to amphi-Atlantic groups, two to East-Atlantic groups and one to a West-Atlantic group, whereas *Salix herbacea* grouped together with Greenland and Iceland with separate subgroups in each of the three regions (Fig. 1). The highest proportion of genetic groups was shared with Iceland, whereas less than five genetic groups were shared with most other regions.

For 23 of the 25 species, populations from the recipient islands were successfully assigned to one (*n* = 22), two (*n* = 17) or more (*n* = 7) source regions (Fig. 1 and Table 1) [see also **Supporting Information**]. Assignment of *Arabis alpina* and *Carex rufina* was not possible because of lack of genetic diversity, and five recipient island × species combinations had to be excluded because the direction could not be determined **[see Supporting Information]**. On average, two source regions had to be inferred to account for all markers observed in the recipient island (Table 1). Only for the Faroe Islands, the most important source region (Scandinavia/Great Britain, 285 km away) was the geographically closest one (Fig. 2). Iceland is only 280 km away from East Greenland, but populations were allocated mainly to Northwest Europe, with Shetland (775 km) and Norway (965 km) being closest. Jan Mayen is 100 km closer to East Greenland than Iceland, where most populations allocated to. Despite the large geographic distance, Northwest Russia was the single most important source region for both East Greenland and Svalbard, although western source regions were also important (Table 1 and Fig. 2).

**Figure 3.**
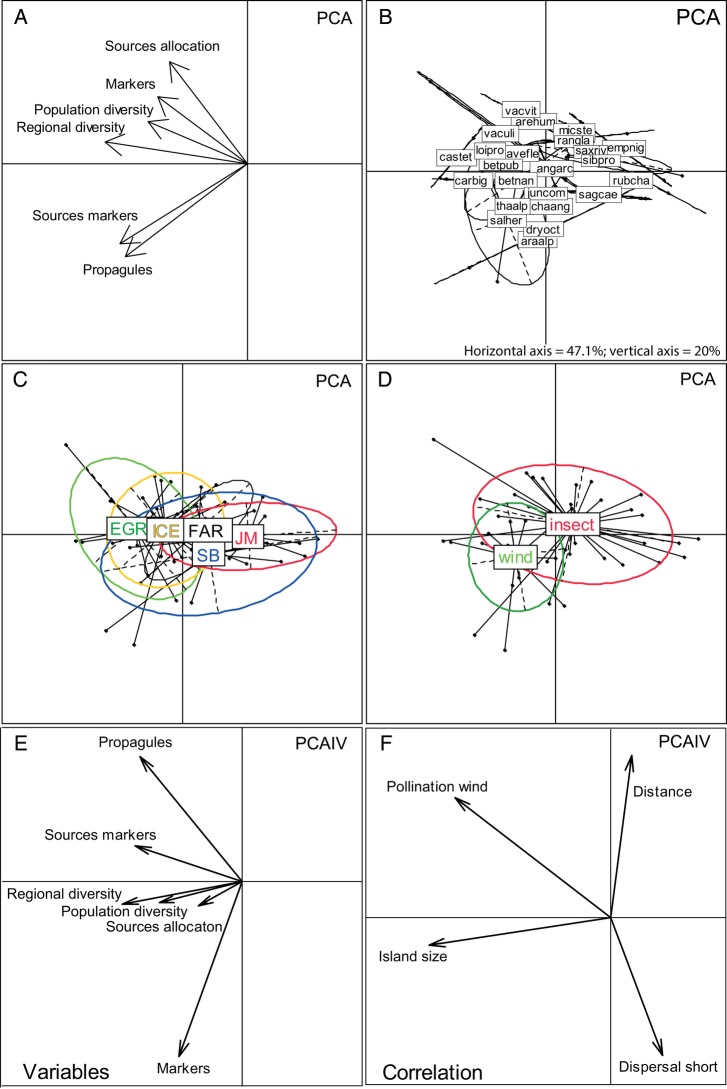
Principal component analyses (PCAs) of six variables expressing the genetic founder effect (see Methods) in the five recipient islands East Greenland (EGR), Iceland (ICE), Svalbard (SB), the Faroe Islands (FAR) and Jan Mayen (JM) relative to the source regions (*n* = 38). The founder effect is increasing from left to right in all panels. (A–D) Principal component analyses showing correlation among the variables (A) and differences in intensity of the founder effect among species (B, some names slightly moved for visibility), recipient islands (C) and pollination mode (D). (E and F) Principal component analyses of instrumental variables (PCAIV), which show to what extent the distance between source and recipient island, size of the recipient island, dispersal adaptation (long- or short-distance) and mode of pollination were correlated with the intensity of the founder effect (ordination taking into account the effect of independent variables).

In the PCA of the six measures of the founder effect, all measures were more or less correlated with axis 1 (horizontal axis), which explained 47.1 % of the variation (Fig. 3A–D). The proportion of regional genetic diversity was most strongly correlated with axis 1, whereas the other five measures were also correlated with axis 2 (vertical axis, 19.9 % of the variation), positively or negatively so. The five recipient islands were placed along the first axis according to their size, although with considerable overlap (Fig. 3C). In the PCAIV, 30.5 % of the variation was explained by the four independent variables (Fig. 3E and F). Island size was mainly related to the first axis, showing the strongest founder effect in small recipient islands. Pollination mode was strongly related to both the first and second axes, with wind-pollinated species being characterized in particular by a higher number of propagules and sources for markers. Dispersal distance was correlated with the second axis. It was negatively correlated with the proportion of markers in the recipient island, but positively with the number of propagules. Dispersal type was also correlated to the second axis, but in opposite direction, partly reflecting that distance was on average somewhat shorter for species lacking adaptations to wind- or animal-dispersal (641 km, SD = 223) than those possessing such adaptations (750 km, SD = 253, difference not significant, **see** **Supporting Information—Table S2**). Lack of adaptations to dispersal was indeed associated with a higher proportion of markers but fewer propagules (Fig. 3F).

**Figure 4.**
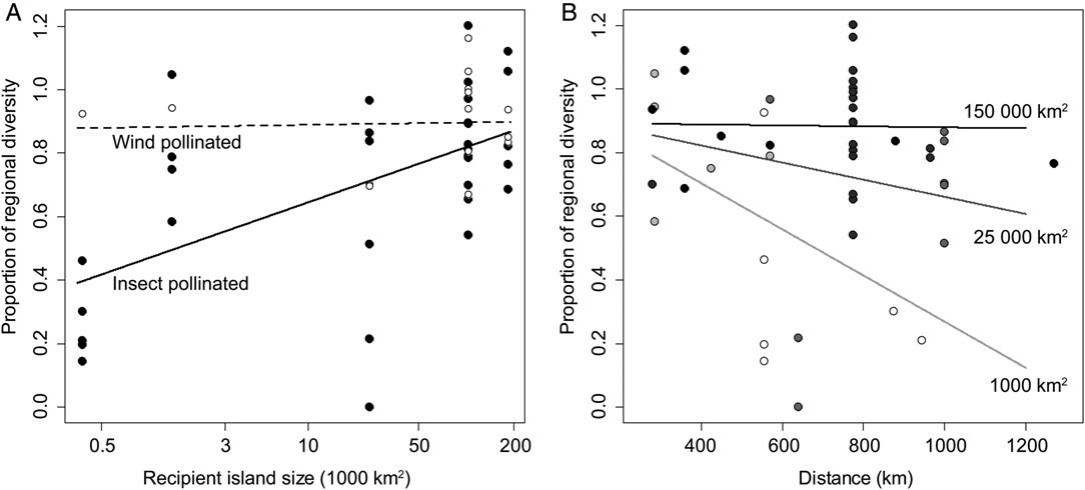
Proportion of regional genetic diversity found on islands relative to source region in relation to (A) size of the recipient island and mode of pollination (open circles represent wind pollinated and black circles indicate insect pollinated) and (B) distance to source region and size of the recipient island (increasing darkness reflects increasing island size).

Variation in the proportion of regional diversity in the recipient island was equally well explained by (i) a model including island size and pollination mode with (best model) or without interaction (AICc difference to the best model 1.05) or (ii) a model with island size, distance from source as well as their interaction (AICc difference 0.68, **Supporting Information—Table S4**) as fixed effects. For insect-pollinated species, the proportion of regional diversity decreased by 0.076 (SE = 0.018, 95 % CI = 0.043–0.113) for reduction by one of the natural logarithm of island size (for instance from 1096 to 403 km^2^ or from 162 755 to 59 874 km^2^), but this was not the case for wind-pollinated species (marginally significant interaction; Fig. 4A, Table 2). According to the second model, the proportion of regional diversity in the recipient island decreased with distance to source region for smaller islands, but less so for the largest ones (slope: −0.072 per 100 km for an island of 1000 km^2^ versus −0.001 for an island of 150 000 km^2^; marginally significant interaction, Fig. 4B and Table 2).

**Table 2.** Parameter estimates for the two most suitable LMMs with REML explaining the proportion of regional genetic diversity in the target island compared with that in the source region (*n* = 46) in function of mode of pollination (insect or wind; reference level is insect), the natural logarithm of island size in square kilometres and distance to main source region (parameter estimates per 100 km increase in distance). Species was included as random effect and variance ± standard deviation is given.

	Fixed effect	Estimate	CI	SE
(1)				
Regional diversity ∼ pollination × island size	Intercept	−0.052		0.185
Species 0	Log(size)	0.076	0.039 to 0.112	0.018
	Pollination wind	0.912	0.050 to 1.754	0.416
	Pollination wind × log(size)	−0.073	−0.157 to 0.005	0.039
(2)				
Regional diversity ∼ island size × distance	Intercept	1.129		0.459
Species 0.004 ± 0.062	Log(size)	−0.020	−1.049 to 0.077	0.045
	Distance (100 km)	−0.170	−0.318 to −0.018	0.072
	Distance × size	0.014	−0.0004 to 0.028	0.007

### Floristic data

The number of species occurring in the five recipient islands were 369–388 in East Greenland, 428–430 in Iceland, 180–181 in Svalbard, 282–293 in the Faroe Islands and 65 in Jan Mayen **[see Supporting Information—Table S3]**. There were 1, 7, 3, 2 and 0 endemic species in the recipient islands, respectively. Thus, species diversity showed the same overall pattern as the genetic data, with highest species diversity and weakest founder effect in larger islands, and lowest species diversity and strongest founder effect in smaller islands (Fig. 3C, Table 1).

Assuming that 92 % of the species colonized postglacially, and using the average number of propagules per species arriving to each recipient island from Table 1, we estimated the following number of years between each successful colonization event: East Greenland 3.3, Iceland 3.5, Svalbard 6.1, Faroe Islands 9.2 and Jan Mayen 42.0. Similarly, using the number of species per island (Fig. 2) and assuming that 92 % colonized postglacially, the years between each successful species establishment were: East Greenland 36.5, Iceland 32.9, Svalbard 54.2, Faroe Islands 49.5 and Jan Mayen 214.0.

All recipient islands showed high floristic similarity with several potential source regions, but with a clear east–west pattern (Fig. 2). For Iceland and the Faroe Islands, the potential source regions showing highest floristic similarities, Fennoscandia and Great Britain, were also identified as source regions by the genetic data. Svalbard had high floristic similarities with both East Canada and Russia, whereas Russia was identified as a major source region by the genetic data. For Jan Mayen, highest floristic similarity was with East Greenland whereas the genetic data identified Iceland as the main source region. For East Greenland, we found highest floristic similarities to West Greenland, Canada and Scandinavia, highest proportion of shared genetic groups with West Greenland, Svalbard and Iceland, whereas the assignment test suggest highest colonization rates from the North Russia followed by West Greenland/Canada (Fig. 2).

## Discussion

We have presented the first comprehensive study of LDD to oceanic islands based on combined population genetic and floristic similarity analyses. We show that the relative intensity of the founder effect is similar at the level of species and genes, and broadly corresponds to the predictions of the Island Equilibrium Theory (MacArthur and Wilson 1967). This indicates that species and genetic diversity on islands are shaped by the same processes. Compared with the floristic data, the genetic data give more detailed information particularly as it allows identification of source regions and estimating the number of colonization events. The genetic data also allowed us to quantify the founder effect in relation to island size, distance to source region and species traits.

### Source regions and colonization patterns

We were able to identify postglacial dispersal routes for most species (Figs 1 and 2), [see also **Supporting Information**], and only find indications of *in situ* glacial persistence in 2 of the 48 combinations of species and recipient islands we analysed here (Table 1; **Supporting Information**, Westergaard *et al*. 2010, 2011). It is still possible that glacial survivor populations did exist but remained undetected in our analyses because they were swamped by postglacial immigrants; however, this scenario would also involve postglacial LDD. Also, our revision of the flora confirmed earlier analyses that the number of endemic species is low on these islands (Brochmann *et al*. 2003), indicating a young age of the local floras.

**Figure 5.**
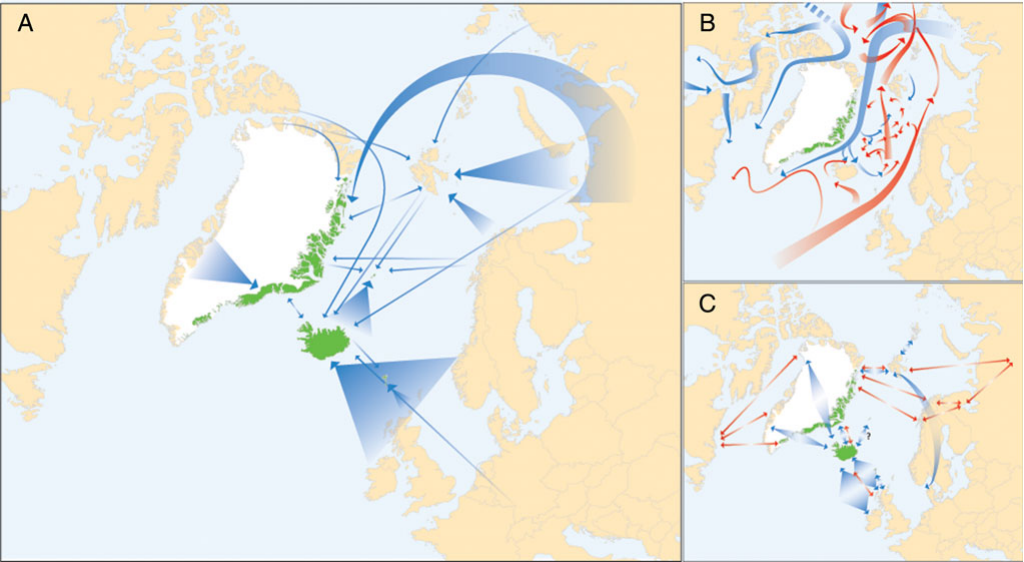
(A) Main (thick arrows) and additional (thin arrows) LDD routes of plants in the North Atlantic area inferred from genetic and floristic data (cf. Fig. 1). (B) Sea surface circulation patterns in the North Atlantic area (blue: cold water, red: warm water). (C) Main migration routes for geese species (thick blue arrows) and the supposedly efficient seed disperser *Plectrophenax nivalis* (snow bunting, thin red arrows) in the North Atlantic area (based on Madsen *et al*. 1999; Lyngs 2003).

The differences between our floristic and genetic analyses with respect to relative importance of source regions (Fig. 2) may have been affected by the selection of species for the genetic analyses, and in the case of East Greenland, also by the delimitation of this region as the proportion of eastern and western species varies (Elven *et al*. 2011). The genetic data were nevertheless most informative; in cases where the same species occurred in many regions, we could identify with reasonable certainty which and how many of them actually served as sources. We therefore give priority to the inferences based on the genetic data in our summary of dispersal routes in the amphi-Atlantic region (Fig. 5).

If LDD is a mainly stochastic process, we would expect that the closest potential source region served as the most important one, as the probability of a dispersal event decreases with distance (Nathan 2006; Nathan *et al*. 2008). On the contrary, with a single exception (the Faroe Islands) we found that the closest potential source region was not the most important one in the North Atlantic. The most extreme case was Iceland, where the main source regions Scandinavia/Great Britain are situated 2.8–4.5 times further away than East Greenland. In addition, gene-based inference of the same main source region was made for 18 of the 19 species analysed, consistent with the floristic similarities (Fig. 2). Thus, although the distance to source region has been found previously to be the second most important factor in determining species diversity on oceanic islands based on stochasticity (after island size, Weigelt and Kreft 2013), our results suggest that other, deterministic factors also are important in determining source and direction of LDD events.

Dispersal vectors are an important deterministic factor affecting LDD patterns (Nathan *et al*. 2008). Although it has been claimed that North Atlantic floras are poorly adapted for LDD based on analyses of propagule morphology (Löve and Löve 1963), dispersal vectors such as wind, sea water or birds may still lead to dispersal over long distances, regardless of morphological dispersal adaptations (Higgins *et al*. 2003; Nathan 2006). The North Atlantic Current connects the Faroe Islands, Iceland and Jan Mayen eastwards to Northwest Europe, whereas the East Greenland Current connects East Greenland, Iceland and Jan Mayen to North Greenland, Svalbard and North Russia (Fig. 5). The similarity between the main current pattern and the inferred dispersal routes is intriguing (compare Fig. 5A and B), and observations of driftwood also suggest dispersal along these routes (Johansen and Hytteborn 2001). Analyses of global patterns of species diversity on islands also indicate that ocean currents can be important (Weigelt and Kreft 2013). The dominant wind directions in the Atlantic region largely follow the ocean currents (http://go.grolier.com/atlas?id=mtlr089), making it hard to distinguish dispersal by wind from dispersal by ocean currents. The main bird migration routes connect Iceland and the Faroe Islands to Great Britain (Löve and Löve 1963; Johansen and Hytteborn 2001). In contrast to the sea currents, the bird migration routes connect East Greenland to Northwest Europe (Lyngs 2003). Many different bird species may carry seeds (Ridley 1930; Nogales *et al*. 2012). Especially Arctic geese migrate in huge numbers along these routes (Fig. 5C). Colonization of East Greenland from Svalbard and North Russia may have been caused by the only Arctic passerine bird, the snow bunting (Fig. 5C), as it is more likely to carry seeds than other birds in northern areas (Fridriksson 1970). Thus, we consider it likely that several dispersal vectors have contributed to colonization of the recipient islands analysed here. In addition, historical factors may have been important. More species might have been available to colonize from Europe during the last glaciation, where numerous fossils indicate presence of a widespread and well-developed Arctic flora during the LGM (Hultén and Fries 1986). In contrast, no LGM fossils have been found in South and East Greenland, where the possibility of glacial survival is still disputed (Cremer *et al*. 2008; Böcher 2012). However, as the patterns of colonization we inferred fit well with the main dispersal vectors, and as historical factors cannot explain the inferred colonization of East Greenland, our data support in the first place the importance of dispersal vectors.

### Factors determining the founder effect

As expected, we found fairly strong correlations among the six measures of the founder effect (Fig. 3). Species that traced back to several source regions, or for which a high minimum number of propagules was inferred, experienced the least reduction in genetic diversity following colonization. A similar pattern has been observed for invasive species (Dlugosch and Parker 2008). We also found a stronger genetic founder effect in smaller islands, congruent with the patterns of species diversity (Figs 2 and 3), in agreement with the species-area effect as predicted by island theory (MacArthur and Wilson 1967; Triantis *et al*. 2012). This effect may be explained by stochastic processes acting on small populations (Frankham 2005) and/or a lower probability of small islands to receive diaspores (Patiño *et al*. 2013). On Jan Mayen, the active volcano may have amplified the initial founder effect by exterminating plant populations. For the small islands, the founder effect also increased with distance to source region (Fig. 4 and Table 2). Similarly, a stronger bottleneck has been observed on remote Pacific Islands than on the Canary Islands (Whittaker and Fernández-Palacios 2007). An increase in founder effect with distance is expected from island theory, and this effect is also expected to be stronger for small than for large islands (Jaenike 1973).

In our analyses, the founder effect was somewhat unexpectedly determined more by mode of pollination than by adaptation to seed dispersal, whereas dispersal distance was poorly related to adaptation to seed dispersal (Fig. 3) **[see Supporting Information—Table S2]**. In our previous study of Svalbard, we also found that the intensity of the founder effect was only weakly related to adaptation to dispersal (Alsos *et al*. 2007). Thus, at dispersal distances of more than 280 km, morphological adaptations to dispersal seem to be of minor importance although they are important for overall gene flow within species (Thiel-Egenter *et al*. 2009; Alsos *et al*. 2012). At larger distances, other factors such as stochasticity and dispersal vectors may be more important for long-distant colonization (Higgins *et al*. 2003; Nathan 2006; Vargas *et al*. 2012).

Long-distance dispersal of pollen in wind-pollinated species may have caused a less severe founder effect compared with insect-pollinated species (Figs 3 and 4). However, this appears unlikely since long-distance pollination typically has been documented only over a few hundred metres, rarely up to 160 km (Ashley 2010). The average dispersal distance of 370–856 km to our five recipient islands thus seems to make successful long-distance pollination unlikely. Rather, since most insect-pollinated species in our study are mixed maters, and as lack of pollinators can shift mating towards self-pollination (Kevan 1972; Tikhmenev 1985), we suggest that the more severe founder effect we found in insect-pollinated species may have been caused by increased inbreeding during the establishment phase. Whether the founder effect in general is stronger affected by pollination mode than by dispersal distance could be investigated by, for example, comparing pollination ecology and inbreeding rates in pioneer populations on islands or glacier forelands with those of well-established sites at different distances.

The overall low founder effect and high species diversity we observed in East Greenland and Iceland support the hypothesis that LDD is frequent in Arctic plants (Alsos *et al*. 2007), contrary to the suggestion that most Arctic islands are unsaturated with species due to dispersal limitations (Hoffmann 2012). Also, rate of successful species colonization found for these islands (one per every 33–214 years) is high compared with, for example, Azores (1 per 40 000 years, Schaefer 2003) and Hawaii (1 per 20 000–250 000 years, Sohmer and Gustafson 1987). However, we also identified both island size and colonization distance as limiting factors for LDD. Independent of island size and distance (Figs 3 and 4), pollination mode was important for the extent of gene flow. A better knowledge of how these factors affect the founder effect can lead to more precise predictions about range shifts in species with different traits as well as to island (or fragmented habitats) of different sizes and distances to source regions.

## Conclusions

Our analyses of floristic and genetic patterns in the North Atlantic area suggest that species diversity and genetic diversity may have been shaped to a large degree by similar processes. The large-scale patterns we inferred from both floristic and genetic data were congruent among many species and consistent with likely dispersal vectors, indicating that deterministic factors are important in determining LDD in addition to purely stochastic ones. This is supported by the clear effect of island size on the intensity of the genetic founder effect, mirrored by species diversity. As past colonization typically occurred from more than one source region, we may expect future colonization to be complex as well, but to be governed to some extent by deterministic processes. Assuming that dispersal vectors are constant, the same main dispersal routes may be expected in the future as in the past. However, the current reduction of the extent of sea ice may limit dispersal, whereas anthropogenic dispersal may increase it. By taking into account the main determinants of the genetic founder effect and the complexity of dispersal routes when modelling future distribution of species and genes, we may improve our ability to forecast effects of the ongoing climate change.

## Sources of Funding

The work was supported by the Research Council of Norway (grant numbers 150322/720 and 170952/V40 to C.B. and 230617/E10 to I.G.A.).

## Contributions by the Authors

I.G.A. conceived the idea and drafted the manuscript together with D.E.; I.G.A., D.E., P.B.E., K.B.W., P.S., A.T. and S.B. analysed the genetic data; C.B. lead the project compiling the genetic data; H.S. and R.E. compiled the floristic data; I.G.A. and D.E. did the statistical analyses and all co-authors commented on the manuscript.

## Conflict of Interest Statement

None declared.

## Supporting Information

The following additional information is available in the online version of this article –

**File S1.** Supporting information containing details of genetic and statistical analyses, R script for estimating number of propagules.

**Table S1.** Data used for estimating founder effect and dispersal routes and traits of the 25 species analysed.

**Table S2.** Significant values of pair-wise association among the size of the island, distance to source region and four species traits.

**Table S3.** A compiled list of vascular plant taxa in recipient regions and occurrences of recipient region taxa in the potential source regions.

**Table S4.** Model selection using Akaike's information criterion.

Additional Information
