# Supplementary material for: Long-distance plant dispersal to North Atlantic islands: colonization routes and founder effect
Source: AoB Plants. 2015 May 14;7:plv036. doi: 10.1093/aobpla/plv036 (PMC4432000; doi:10.1093/aobpla/plv036)
Supplement: Additional Information [file supp_7_plv036_index.html]

Long-distance plant dispersal to North Atlantic islands: colonization routes and founder effect — Additional Information 

# Long-distance plant dispersal to North Atlantic islands: colonization routes and founder effect

## Additional Information

Additional Information

**Files in this Data Supplement:**

- Additional Information - Docx file
