# Supplementary material for: Long-distance plant dispersal to North Atlantic islands: colonization routes and founder effect
Source: AoB Plants. 2015 May 14;7:plv036. doi: 10.1093/aobpla/plv036 (PMC4432000; doi:10.1093/aobpla/plv036)
Supplement: Additional Information [file supp_plv036_plv036supp_file.docx]

**SUPPORTING INFORMATION for Long-distance plant dispersal**

**AFLP analyses of *Sibbaldia procumbens***

We collected leaf samples from up to 10 individuals per population (site), if possible at 5-10 m intervals, and dried them in silica gel. The extraction protocol followed ([Ziegenhagen *et al.*, 1993](#_ENREF_34)) and the PCR and fragment analyses followed ([Jørgensen *et al.*, 2006](#_ENREF_17)) with minor modifications. Four primer combinations were chosen: *Eco*RI AGG(VIC) – *Mse*I CAA, *Eco*RI ACA(NED) – *Mse*I CAT, *Eco*RI ACC(6-FAM) – *Mse*I CAG, and *Eco*RI ACA(NED) – *Mse*I CAC. The AFLP electropherograms were visualised in GeneMapper 3.7 (Applied Biosystem, Foster City) and scored in R ([R_Core_Team, 2013](#_ENREF_22)) using the semi-automated AFLPScore, see ([Whitlock *et al.*, 2008](#_ENREF_33)). Leaf samples and DNA extracts from most populations are deposited in the DNA Bank at the Natural History Museum, University of Oslo (O), along with voucher specimens.

**Analyses of genetic groups**

To infer geographically consistent genetic groups, we used the Bayesian model-based clustering approach implemented in Structure 2.3.4 ([Pritchard *et al.*, 2000](#_ENREF_21)), assuming a no admixture model with uncorrelated allele frequencies (which is appropriate for data collected over a large geographical scale) and recessive alleles ([Falush *et al.*, 2007](#_ENREF_13)). The number of genetic groups (*K*) was estimated based on 1,000,000 Markov Chain Monte Carlo (MCMC) iterations, with a burn-in period of 200,000 iterations. Ten replicates were run for each *K* for *K* = 1-10 on the Bioportal at the University of Oslo ([www.bioportal.uio.no/](http://www.bioportal.uio.no/), a service now transferred to https://lifeportal.uio.no). Similarity of results among different runs for the same value of *K* was calculated according to ([Rosenberg *et al.*, 2002](#_ENREF_24)) using the R script Structure.sum version 2011 updated from ([Ehrich *et al.*, 2007](#_ENREF_5)). The *K* value with the highest likelihood, with a high similarity coefficient (> 0.85, ([Rosenberg *et al.*, 2002](#_ENREF_24)) between runs, with high delta*K* ([Evanno *et al.*, 2005](#_ENREF_12)), and with most individuals clearly grouped was chosen as the number of main genetic groups. The same analyses were also run for each of the main genetic groups obtained in total dataset for each species to search for a hierarchical structure. As our aim was to determine distinct geographic regions as candidate source regions for colonisation of the North Atlantic islands, new runs using the locprior option ([Hubisz *et al.*, 2009](#_ENREF_15)) and specifying each sampling site as locality were performed when many populations were subdivided among several clusters. Finally, if different individuals in a population belonged to different genetic groups, the population was attributed to the group in which most individuals were placed. To obtain a final optimal grouping of sampling localities, we also evaluated the results of the Structure analyses by comparison with BAPS analyses ([Corander and Marttinen, 2005](#_ENREF_4)), Principal Coordinate analyses as implemented in NTSYS-pc 2.02h ([Rohlf, 1990](#_ENREF_23)), and/or Neighbor Joining trees using TREECON 1.3b ([Van de Peer and De Wachter, 1994](#_ENREF_29)).

For the assignment tests, potential source regions comprised all geographically consistent genetic main groups identified in mainland areas. If a species occurred in more than one of the five recipient islands, other recipient islands were included as separate potential source regions. In cases where genetic groups were restricted to several islands within the amphi-Atlantic region (Fig. S1), an additional assignment test of this group was performed to infer its origin and dispersal direction (see below). In such cases, regions harbouring the same genetic group but being more distant from the source region than the recipient island were omitted in the allocation tests (concerns *Angelica archangelica, Betula nana, Micranthes stellaris,* and *Salix herbacea*), whereas in *Cassiope tetragona,* Scandinavia and Svalbard were omitted as source regions as the overall colonization direction was from west to east ([Eidesen *et al.*, 2007a](#_ENREF_6)). These refinements served to increase confidence in determining correct dispersal direction. For *Dryas octopetala,* East Greenland was not regarded as a potential source region for individuals from Iceland, since all individuals analysed from East Greenland appear to be of interspecific hybrid origin ([Skrede *et al.*, 2006](#_ENREF_27)). For *Empetrum nigrum,* we omitted assignment of Svalbard and East Greenland populations as the low differentiation between Svalbard, East Greenland and Russia made the inferred direction of dispersal uncertain. No assignment test was performed for populations of *Arenaria humifusa* and *Saxifraga rivularis* on Svalbard as they likely survived the last glaciation there ([Westergaard *et al.*, 2010](#_ENREF_31); [Westergaard *et al.*, 2011b](#_ENREF_32)). The reciprocal allocation between Jan Mayen and Iceland (*R. glacialis*) and between Jan Mayen and East Greenland (*S. herbacea*, Fig. S1) most likely reflect colonisation from the larger regions to the small island Jan Mayen.

**Additional assignment tests**

For species having genetic groups that were restricted to islands within the amphi-Atlantic region, an assignment test of this group was performed to find its origin.

*Angelica archangelica*: As all 126 individuals in the amphi-Atlantic group (red triangles on Fig. S1) allocated NW Europe, Greenland was omitted as potential source region for the allocation of individuals from Island whereas both Greenland and Island were omitted for the allocation of individuals from the Faroe Islands.

*Betula nana*: The main source for the Structure group from Greenland and Iceland (all yellow symbols on Fig. S1) was Northwest Europe (74 of 110 individuals). The 36 individuals that were not allocated were all split between West Russia and Europe/Iceland. Thus, 100 % of the allocation was to eastern source regions. Accordingly, both West and East Greenland were omitted as potential source regions for Iceland whereas West Greenland was omitted as potential source region for East Greenland.

*Cassiope tetragona*: The majority of the individuals in the East Greenlandic– Svalbard-N Scandinavian Structure group (red circles on Fig. S1) allocated to West Greenland (34 individuals) or Canada (68 individuals), and the 109 that were not allocated were split between groups in these regions. Therefore, Svalbard and N Scandinavia were omitted as source regions when assigning individuals from East Greenland.

*Micranthes stellaris*: For *M. stellaris,* a northern and a southern dataset were available. Analyses of the northern dataset (all red symbols on Fig. S1) show that the Atlantic Structure subgroup (red triangles) were allocated to the Scandinavian-Scottish group (red circles, 18 individuals), although 51 individuals were not allocated as the Scandinavian-Scottish group was poorly differentiated from a Structure subgroup consisting of only one population in Scotland (red square). The southern dataset (all other symbols, including 20 individuals from the northern dataset) clearly show that all northern populations originated from populations in the Massif Central (Ehrich and Tribsch, unpublished). Accordingly, Iceland and Greenland were omitted as potential source regions for the individuals on the Faroe Islands whereas Greenland was omitted as potential source region for the individuals from Iceland. The results obtained (Fig S1) were in accordance with analyses of the southern dataset.

*Salix herbacea*: Forty-five individuals from the East Greenlandic-Jan Mayen-Icelandic Structure group (purple circles, squares and triangle on Fig. S1) were allocated to South Scandinavia-Scotland and 17 to North Scandinavia, whereas 14 were divided between these two regions. An eastern source region of the Atlantic group is also in accordance with fossil records ([Alsos *et al.*, 2009](#_ENREF_2)). Thus, East Greenland and Jan Mayen were omitted as potential source regions when assigning individuals from Iceland. For Jan Mayen, East Greenland was not omitted as potential source region as the shorter distance from Jan Mayen than Iceland makes it a likely source region.

**Estimating minimum numbers of propagules**

The minimum number of propagules was estimated as the number of individual genotypes from the main source region which could have brought all markers observed in the recipient island ([Alsos *et al.*, 2007](#_ENREF_1)). It was estimated by choosing successively the individuals from the main source region carrying most of the markers present in the recipient island. The main source region was defined as the region to which most individuals were assigned by the allocation procedure implemented in AFLPop. When some markers present in the recipient island were absent from the main source region, additional “propagules” were inferred. A second source region was chosen as the region where most of the recipient markers missing in the main source region were found, and the individuals carrying most of those markers were chosen, until all recipient markers were accounted for. The following script was written for R:

## The first line of the input file should contain 1 for all

## markers present in the recipient population and 0 for all those which are absent.

## Subsequent lines contain the genotypes of the individuals from the source region

Propagu <- function(input) {

#sourcex <- read.table(infile, header=TRUE, row.names=1)

sourcex=input

sinkm <- sourcex[1, ]

indnb <- dim(sourcex)[1]-1

manb <- dim(sourcex)[2]

sourcex <- as.matrix(sourcex[2:(indnb+1), ])

propagules <- 0

present <- sourcex[ , sinkm==1]

while (sum (sinkm) - sum(propagules) >1 ) {

sums <- apply(present, 1, sum)

propagules <- c(propagules, max(sums))

if (length(sums[sums==max(sums)]) > 1) {

x <- sample(which(sums==max(sums)), 1) } else {

x <- which(sums==max(sums))}

present1 <- present[-x, present[x, ]==0]

present <- present1

rm(present1)

rm(sums)

}

if(sum(sinkm) - sum(propagules) == 0) return(list(length(propagules)-1, propagules))

if(sum(sinkm) - sum(propagules) == 1) return(list(length(propagules), c(propagules, 1)))

}

Propag <- function(infile, replic=100) {

sourc <- read.table(infile, header=TRUE, row.names=1)

res <- vector("numeric", replic)

for (i in 1:replic) {

res[i] <- Propagu(sourc)[[1]]

}

return(list(min(res), res))

}

**Table S1.** Data used for estimating founder effect and dispersal routes, and traits of the 25 species analysed. Number of populations (Pop) and individuals (Ind) analysed for AFLPs, number of polymorphic markers (PM) and source reference for the AFLP data (AFLP data) are provided. The AFLP data have either been published with full information on sampling, published with maps showing sampling points marked according to main genetic group ([Eidesen *et al.*, 2013](#_ENREF_9)), or are unpublished (only name of investigator given here). Species traits include adaptation to dispersal (Short, Long), growth form (Woody or Herbaceous perennial), main mode of pollination (insect, wind), and breeding system (cross-fertilization, self-fertilization, or mixed).

|  | Genetic data | | | |  | Species traits | | | | |  |
| --- | --- | --- | --- | --- | --- | --- | --- | --- | --- | --- | --- |
| Species | Pop | Ind | PM | AFLP data |  | Dispersal | Growth form | Pollination | Breed­ing |  | |
| *Angelica archangelica* L. | 41 | 405 | 140 | ([Alsos *et al.*, 2012](#_ENREF_3); [Eidesen *et al.*, 2013](#_ENREF_9)) |  | S | H | insect | cross |  | |
| *Arabis alpina* L. | 36 | 305 | 242 | ([Ehrich *et al.*, 2007](#_ENREF_5)) |  | S | H | insect | selfing |  | |
| *Arctous alpinus* (L.) Nied. | 38 | 336 | 216 | ([Alsos *et al.*, 2012](#_ENREF_3); [Eidesen *et al.*, 2013](#_ENREF_9)) |  | L | W | insect | mixed |  | |
| *Arenaria humifusa* | 17 | 92 | 110 | ([Westergaard *et al.*, 2011b](#_ENREF_32)) |  | S | H | insect | mixed |  | |
| *Avenella flexuosa* (L.) Drejer | 37 | 343 | 162 | ([Alsos *et al.*, 2012](#_ENREF_3); [Eidesen *et al.*, 2013](#_ENREF_9)) |  | L | H | wind | cross |  | |
| *Betula nana* L. | 65 | 528 | 115 | ([Eidesen, 2007](#_ENREF_7); [Eidesen *et al.*, 2013](#_ENREF_9)) |  | L | W | wind | cross |  | |
| *Betula pubescens* Ehrh. | 35 | 279 | 128 | ([Eidesen, 2007](#_ENREF_7); [Eidesen *et al.*, 2013](#_ENREF_9)) |  | L | W | wind | cross |  | |
| *Carex bigelowii* Torr. ex. Schwein. | 23 | 186 | 204 | ([Schönswetter *et al.*, 2008](#_ENREF_26)) |  | S | H | wind | cross |  | |
| *Carex rufina* Drejer | 11 | 69 | 5 | ([Westergaard *et al.*, 2011a](#_ENREF_30)) |  | S | H | wind | selfing |  | |
| *Cassiope tetragona* (L.) D.Don | 58 | 579 | 171 | ([Eidesen *et al.*, 2007a](#_ENREF_6)) |  | S | W | insect | mixed |  | |
| *Chamerion angustifolium* (L.) Holub | 64 | 462 | 189 | ([Alsos *et al.*, 2012](#_ENREF_3); [Eidesen *et al.*, 2013](#_ENREF_9)) |  | L | H | insect | mixed |  | |
| *Dryas octopetala* L. | 51 | 459 | 154 | ([Skrede *et al.*, 2006](#_ENREF_27)) |  | L | W | insect | mixed |  | |
| *Empetrum nigrum* L. s.lat. | 46 | 413 | 78 | ([Alsos *et al.*, 2007](#_ENREF_1)) |  | L | W | insect | mixed |  | |
| *Juniperus communis* L. | 53 | 446 | 146 | ([Alsos *et al.*, 2012](#_ENREF_3); [Eidesen *et al.*, 2013](#_ENREF_9)) |  | L | W | wind | cross |  | |
| *Loiseleuria procumbens* (L.) Desv. | 41 | 404 | 79 | ([Eidesen, 2007](#_ENREF_7)) ([Eidesen *et al.*, 2013](#_ENREF_9)) |  | S | W | insect | mixed |  | |
| *Micranthes stellaris* (L.) Galasso, Banfi & Soldano | 25 | 182 | 309 | ([Alsos *et al.*, 2012](#_ENREF_3); [Eidesen *et al.*, 2013](#_ENREF_9)) |  | S | H | insect | mixed |  | |
| *Ranunculus glacialis* L. | 85 | 268 | 208 | ([Schönswetter *et al.*, 2003](#_ENREF_25)) |  | S | H | insect | cross |  | |
| *Rubus chamaemorus* L. | 45 | 398 | 173 | ([Alsos *et al.*, 2012](#_ENREF_3); [Eidesen *et al.*, 2013](#_ENREF_9)) |  | L | H | insect | cross |  | |
| *Sagina caespitosa* | 16 | 133 | 202 | ([Westergaard *et al.*, 2011b](#_ENREF_32)) |  | S | H | insect | mixed |  | |
| *Salix herbacea* L. | 41 | 399 | 250 | ([Alsos *et al.*, 2009](#_ENREF_2)) |  | L | W | wind | cross |  | |
| *Saxifraga rivularis* L. | 22 | 207 | 78 | ([Westergaard *et al.*, 2010](#_ENREF_31)) |  | S | H | insect | selfing |  | |
| *Sibbaldia procumbens* | 18 | 151 | 62 | Birkeland unpubl. |  | S | H | insect | mixed |  | |
| *Thalictrum alpinum* L. | 49 | 295 | 268 | ([Alsos *et al.*, 2012](#_ENREF_3)) |  | S | H | wind | mixed |  | |
| *Vaccinium uliginosum* L. | 131 | 958 | 105 | ([Eidesen *et al.*, 2007b](#_ENREF_8)) |  | L | W | insect | mixed |  | |
| *Vaccinium vitis-idaea* L. | 66 | 628 | 115 | ([Eidesen, 2007](#_ENREF_7); [Eidesen *et al.*, 2013](#_ENREF_9)) |  | L | W | insect | mixed |  | |

**Table S2.** Significance values of pairwise association among the size of the island, distance to source region, and four species traits: mode of pollination (insect, wind), the presence of dispersal adaptations (long-distance, short-distance), growth form (woody, herbaceous), and breeding system (cross-fertilization, self-fertilization or mixed). Linear models were used to test for associations involving the continuous variable distance and log(Island size), otherwise Fisher’s exact test was used. In order to account for repeated testing, relationships among variables were considered significant for p < 0.01. Sample size is 46.

|  | Dispersal | Growth form | Pollination | Breeding | Distance |
| --- | --- | --- | --- | --- | --- |
| Growth form | **0.000** |  |  |  |  |
| Pollination | 0.054 | 0.202 |  |  |  |
| Breeding | 0.120 | 0.606 | **0.000** |  |  |
| Distance | 0.125 | 0.619 | 0.822 | 0.260 |  |
| Log(Island size) | 0.216 | 0.188 | 0.258 | 0.561 | 0.149 |

**Table S3.** Compiled list of vascular plant taxa in recipient islands and occurrences of recipient island taxa in the potential source regions. The compilation is based on the Pan Arctic Flora checklist (PAF, Elven *et al.*, 2011) for those of our regions that are included there, otherwise ([Hultén and Fries, 1986](#_ENREF_16)). We used additional sources for the following regions: Iceland ([Kristinsson, 2008](#_ENREF_18)); personal observations by Reidar Elven and Heidi Solstad), Jan Mayen ([Lid, 1964](#_ENREF_19)), the Faeroe Islands ([Hansen, 1966](#_ENREF_14); [Mossberg and Stenberg, 2007](#_ENREF_20)), Great Britain ([Stace, 1997](#_ENREF_28)), and Norway ([Elven, 2005](#_ENREF_10)). The subdivision of Greenland follows the floristic regions defined in PAF ([Elven *et al.*, 2011](#_ENREF_11)). East Canada is defined as including the provinces and territories of Nunavut east of 90° W, Quebec, Newfoundland and Labrador, New Brunswick, Nova Scotia and Prince Edward Island. Nomenclature follows PAF ([Elven *et al.*, 2011](#_ENREF_11)) for the taxa included there, otherwise given as most appropriate according to R. Elven. 1 - confirmed occurrence, 1? - occurrence or taxonomic identity not fully confirmed, and v.t. - the actual recipient island taxon has one or more vicariant taxa in some source regions. The comments row includes information about the vicariant taxa, source region, endemic taxa and other relevant information.

| Taxon | Plant family | Greenland East | Iceland | Svalbard | Faeroe Islands | Jan Mayen | Canada East | Greenland West | Greenland North | N Ural mts | Fennoscandia | Great Britain | C European mts | Recipient region endemics | Comments |
| --- | --- | --- | --- | --- | --- | --- | --- | --- | --- | --- | --- | --- | --- | --- | --- |
| Achillea millefolium L. subsp. lanulosa (Nutt.) Piper | ASTERACEAE |  | 1? |  |  |  | 1 |  |  |  |  |  |  |  |  |
| Achillea millefolium L. subsp. millefolium | ASTERACEAE | 1 | 1 |  |  |  |  |  |  | 1 | 1 | 1 | 1 |  |  |
| Agrostis capillaris L. | POACEAE | 1 | 1 |  | 1 |  |  |  |  | 1 | 1 | 1 | 1 |  |  |
| Agrostis mertensii Trin. | POACEAE | 1 |  |  |  |  | 1 | 1 |  | 1 | 1 |  |  |  |  |
| Agrostis stolonifera L. | POACEAE | 1 | 1 |  | 1 |  | 1 | 1 |  | 1 | 1 | 1 | 1 |  |  |
| Agrostis vinealis Schreb. | POACEAE | 1 | 1 |  | 1 |  | 1 | 1 |  |  | 1 | 1 | 1 |  |  |
| Aira praecox L. | POACEAE |  |  |  | 1 |  |  |  |  |  | 1 | 1 |  |  |  |
| Ajuga pyramidalis L. | LAMIACEAE | 1 | 1 |  |  |  |  |  |  |  | 1 | 1 | 1 |  |  |
| Alchemilla alpina L. | ROSACEAE | 1 | 1 |  | 1 |  |  | 1 |  |  | 1 | 1 | 1 |  |  |
| Alchemilla faeroënsis (Lange) Buser | ROSACEAE |  | 1 |  | 1 |  |  |  |  |  | 1 |  |  |  |  |
| Alchemilla filicaulis Buser subsp. filicaulis | ROSACEAE | 1 | 1 |  | 1? |  | 1 | 1 |  |  | 1 | 1 | 1 |  |  |
| Alchemilla filicaulis Buser subsp. vestita (Buser) M.E.Br. | ROSACEAE | 1 | 1 |  | 1 |  | 1 | 1 |  |  | 1 | 1 | 1 |  |  |
| Alchemilla glabra Neygenf. | ROSACEAE | 1 | 1 |  |  |  |  |  |  |  | 1 | 1 | 1 |  |  |
| Alchemilla glomerulans Buser | ROSACEAE | 1 | 1 | 1 |  | 1 | 1 | 1 |  | 1 | 1 | 1 | 1 |  |  |
| Alchemilla subcrenata Buser | ROSACEAE | 1 | 1 |  |  |  |  |  |  |  | 1 | 1 | 1 |  |  |
| Alchemilla wichurae (Buser) Stefansson | ROSACEAE |  | 1 |  | 1 |  |  |  |  |  | 1 | 1 |  |  |  |
| Allium oleraceum L. | ALLIACEAE | 1 | 1 |  |  |  |  |  |  |  | 1 | 1 | 1 |  |  |
| Alopecurus aequalis Sobol. | POACEAE | 1 | 1 |  |  | 1 | 1 | 1 |  | 1 | 1 | 1? | 1 |  |  |
| Alopecurus ovatus Knapp (A. borealis Trin.) | POACEAE | 1 |  | 1 |  |  | 1 | 1 | 1 | 1 |  | 1 |  |  |  |
| Ammophila arenaria (L.) Link | POACEAE |  |  |  | 1 |  |  |  |  |  | 1 | 1 |  |  |  |
| Andromeda polifolia L. | ERICACEAE | 1 | 1 |  |  |  | 1 | 1 |  | 1 | 1 | 1 | 1 |  |  |
| Angelica archangelica L. subsp. archangelica | APIACEAE |  | 1 |  |  |  |  | 1 |  | 1 | 1 |  |  |  |  |
| Angelica archangelica L. subsp. litoralis (Wahlenb.) Thell. | APIACEAE |  |  |  | 1? |  |  |  |  |  | 1 |  |  |  |  |
| Angelica sylvestris L. | APIACEAE | 1 | 1 |  | 1 |  |  |  |  | 1 | 1 | 1 | 1 |  |  |
| Antennaria alpina (L.) Gaertn. subsp. canescens (Lange) Chmiel. | ASTERACEAE |  | 1 |  |  |  | 1 | 1 |  |  | 0 |  |  |  | Vicariant taxon in Fennoscandia subsp. alpina |
| Antennaria alpina (L.) Gaertn. subsp. porsildii (Ekman) Chmiel. | ASTERACEAE | 1 |  |  |  |  |  | 1 |  |  | 1 |  |  |  |  |
| Antennaria hansii A.Kerner | ASTERACEAE | 1? |  |  |  |  |  | 1 |  |  |  |  |  |  | Endemic to Greenland. Possibly part of the North American A. rosea Greene complex |
| Antennaria media subsp. compacta (Malte) Chmiel. | ASTERACEAE | 1 |  |  |  |  | 1 | 1 | 1 |  |  |  |  |  |  |
| Anthoxanthum nipponicum Honda | POACEAE | 1 | 1 |  | 1 |  | 1 | 1 |  | 1 | 1 | 1 | 1 |  |  |
| Anthoxanthum odoratum L. | POACEAE |  | 1 |  | 1 |  |  |  |  |  | 1 | 1 | 1 |  |  |
| Anthyllis vulneraria L. | FABACEAE | 1 | 1 |  |  |  |  |  |  |  | 1 | 1 | 1 |  |  |
| Arabidopsis arenicola (Richardson ex Hook.) Al-Shehbaz, Elven, D.F.Murray & Warwick | BRASSICACEAE | 1 |  |  |  |  | 1 | 1 |  |  |  |  |  |  |  |
| Arabidopsis petraea (L.) Dorof. subsp. petraea | BRASSICACEAE |  | 1 |  | 1 |  |  |  |  | 0 | 1 | 1 | 1 |  | Vicariant taxon in N Urals and eastwards: subsp. septentrionalis (N.Busch) Elven & D.F.Murray |
| Arabis alpina L. | BRASSICACEAE | 1 | 1 | 1 | 1 | 1 | 1 | 1 |  | 1 | 1 | 1 | 1 |  |  |
| Arctagrostis latifolia (R.Br.) Griseb. | POACEAE | 1 |  | 1 |  |  | 1 | 1 | 1 | 1 | 1 |  |  |  |  |
| Arctodupontia scleroclada (Rupr.) Tzvelev | POACEAE |  |  | 1 |  |  |  |  |  | 1 |  |  |  |  |  |
| Arctophila fulva (Trin.) Andersson | POACEAE |  |  | 1 |  |  | 1 | 1 |  | 1 | 1 |  |  |  |  |
| Arctostaphylos uva-ursi (L.) Spreng. | ERICACEAE | 1 | 1 |  |  |  | 1 | 1 |  |  | 1 | 1 | 1 |  |  |
| Arctous alpina (L.) Nied. | ERICACEAE | 1 |  |  |  | 1 | 1 | 1 |  | 1 | 1 | 1 | 1 |  |  |
| Arenaria humifusa Wahlenb. | CARYOPHYLLACEAE | 1 |  | 1 |  |  | 1 | 1 | 1 |  | 1 |  |  |  |  |
| Arenaria norvegica Gunnerus | CARYOPHYLLACEAE |  | 1 |  |  |  |  |  |  |  | 1 | 1 |  |  |  |
| Arenaria pseudofrigida (Ostenf. & O.C.Dahl) Schischk. & Knorring | CARYOPHYLLACEAE | 1 |  | 1 |  |  |  |  | 1 | 1 | 1 |  |  |  |  |
| Armeria maritima (Mill.) Willd. | PLUMBAGINACEAE |  | 1 |  | 1 |  |  | 1 |  |  | 1 | 1 |  |  |  |
| Armeria scabra Pall. ex Roem. & Schult. | PLUMBAGINACEAE | 1 |  |  |  |  | 1 | 1 | 1 | 1 | 1 |  |  |  |  |
| Arnica angustifolia Vahl subsp. angustifolia | ASTERACEAE | 1 |  | 1 |  |  | 1 | 1 | 1 | 0 | 0 |  |  |  | Vicariant taxa in N Fennoscandia: subsp. alpina (L.) I.K.Ferguson; N Urals and eastwards: subsp. iljinii (Maguire) I.K.Ferguson |
| Arrhenatherum elatius (L.) P.Beauv. ex J.Presl & C.Presl | POACEAE | 1 | 1 |  |  |  |  |  |  |  | 1 | 1 | 1 |  |  |
| Asplenium adiantum-nigrum L. | ASPLENIACEAE |  |  |  | 1 |  |  |  |  |  | 1 | 1 | 1 |  |  |
| Asplenium septentrionale (L.) Hoffm. | ASPLENIACEAE | 1 | 1 |  |  |  |  |  |  | 1 | 1 | 1 | 1 |  |  |
| Asplenium trichomanes L. ssp. trichomanes | ASPLENIACEAE |  | 1 |  | 1 |  | 1 |  |  |  | 1 | 1 | 1 |  |  |
| Asplenium viride Huds. | ASPLENIACEAE | 1 | 1 |  |  | 1 | 1 | 1 |  | 1 | 1 | 1 | 1 |  |  |
| Athyrium distentifolium Tausch ex Opiz subsp. americanum (Butters) Hultén | WOODSIACEAE | 1 |  |  |  |  | 1 | 1 |  |  |  |  |  |  |  |
| Athyrium distentifolium Tausch ex Opiz subsp. distentifolium | WOODSIACEAE | 1 | 1 |  | 1 |  |  |  |  | 1 | 1 | 1 | 1 |  |  |
| Athyrium filix-femina (L.) Roth subsp. filix-femina | WOODSIACEAE | 1 | 1 |  | 1 |  | 0 |  |  | 1 | 1 | 1 | 1 |  | Vicariant taxon in E Canada: subsp. cyclosorum (Rupr.) C.Chr. |
| Atriplex glabriuscula Edmondston | AMARANTHACEAE |  | 1 |  | 1 |  | 1 |  |  |  | 1 | 1 |  |  |  |
| Atriplex littoralis L. | AMARANTHACEAE |  | 1 |  |  |  |  |  |  |  | 1 | 1 |  |  |  |
| Atriplex nudicaulis Boguslaw (A. longipes Drejer subsp. praecox (Hülph.) Turesson) | AMARANTHACEAE |  | 1 |  |  |  | 1 | 1 |  |  | 1 | 1 |  |  |  |
| Avenella flexuosa (L.) Drejer | POACEAE | 1 | 1 |  | 1 |  | 1 | 1 |  | 1 | 1 | 1 | 1 |  |  |
| Bartsia alpina L. | OROBANCHACEAE | 1 | 1 |  | 1 |  | 1 | 1 |  | 1 | 1 | 1 | 1 |  |  |
| Bellis perennis L. | ASTERACEAE |  |  |  | 1 |  |  |  |  |  | 1 | 1 | 1 |  |  |
| Betula nana L. subsp. nana | BETULACEAE | 1 | 1 |  |  | 1 | 0 | 1 |  | 1 | 1 | 1 | 1 |  | Vicariant taxon in E Canada: subsp. exilis (Sukaczev) Hultén. |
| Betula nana L. var. tundrarum (Perfil.) Elven | BETULACEAE |  |  | 1 |  |  | 0 |  |  | 1 |  |  |  |  | Vicariant taxon in E Canada: subsp. exilis (Sukaczev) Hultén. |
| Betula pubescens Ehrh. subsp. tortuosa (Ledeb.) Nyman | BETULACEAE |  | 1 |  |  |  | 0 | 0 |  | 1 | 1 | 1 |  |  | Vicariant taxon in E Canada & W Greenland: perhaps B. minor (Tuck.) Fernald |
| Bistorta vivipara (L.) Delarbre | POLYGONACEAE | 1 | 1 | 1 | 1 | 1 | 1 | 1 | 1 | 1 | 1 | 1 | 1 |  |  |
| Blechnum spicant (L.) Sm. | BLECHNACEAE |  | 1 |  | 1 |  |  |  |  |  | 1 | 1 | 1 |  |  |
| Boechera holboellii (Hornem.) Á.Löve & D.Löve | BRASSICACEAE | 1? |  |  |  |  | 0 | 1 |  |  |  |  |  |  | Endemic to Greenland (W & E). Several close relatives in Canada, esp. B. retrofracta (Graham) Á.Löve & D.Löve |
| Botrychium boreale Milde | OPHIOGLOSSACEAE |  | 1 | 1 |  |  |  | 1 |  | 1 | 1 |  |  |  |  |
| Botrychium lanceolatum (S.G.Gmel.) Ångstr. | OPHIOGLOSSACEAE |  | 1 |  |  |  | 1 | 1 |  |  | 1 |  | 1 |  |  |
| Botrychium lunaria (L.) Sw. | OPHIOGLOSSACEAE | 1 | 1 | 1 | 1 | 1 | 1 | 1 |  | 1 | 1 | 1 | 1 |  |  |
| Botrychium minganense Vict. | OPHIOGLOSSACEAE |  | 1 |  |  |  | 1 | 1 |  |  |  |  |  |  |  |
| Botrychium simplex E.Hitchc. | OPHIOGLOSSACEAE |  | 1 |  |  |  | 1 | 1 |  |  | 1 |  | 1 |  |  |
| Botrychium simplex E.Hitchc. var. 'tenebrosum' | OPHIOGLOSSACEAE |  | 1 |  |  |  |  |  |  |  |  |  |  | End | Unresolved (and unstudied) taxon, possibly a species |
| Braya glabella Richardson subsp. purpurascens (R.Br.) Cody | BRASSICACEAE | 1 |  | 1 |  |  | 1 | 1 | 1 | 1 | 1 |  |  |  |  |
| Braya humilis (C.A.Mey.) B.L.Rob. | BRASSICACEAE | 1 |  |  |  |  | 1 | 1 | 1 |  |  |  |  |  |  |
| Braya linearis Rouy | BRASSICACEAE | 1 |  |  |  |  |  | 1 |  |  | 1 |  |  |  |  |
| Braya thorild-wulffii Ostenf. | BRASSICACEAE | 1 |  |  |  |  | 1 |  | 1 |  |  |  |  |  |  |
| Briza media L. | POACEAE |  |  |  | 1 |  |  |  |  |  | 1 | 1 | 1 |  |  |
| Cakile maritima Scop. subsp. integrifolia (Hornem.) Hyl. ex Greuter & Burdet | BRASSICACEAE |  |  |  | 1 |  |  |  |  |  | 1 | 1 |  |  |  |
| Cakile maritima Scop. subsp. islandica (Gand.) Hyl. ex Elven | BRASSICACEAE |  | 1 |  |  |  |  |  |  |  | 1 |  |  |  |  |
| Calamagrostis canadensis (Michx.) P.Beauv. subsp. langsdorffii (Link) Hultén | POACEAE | 1 |  |  |  |  | 1 | 1 |  | 1 |  |  |  |  |  |
| Calamagrostis inexpansa A.Gray | POACEAE | 1 |  |  |  |  | 1 | 1 |  |  |  |  |  |  |  |
| Calamagrostis neglecta (Ehrh.) P.Gaertn., B.Mey & Scherb. subsp. groenlandica (Schrank) Matuszk. | POACEAE |  | 1 | 1 |  |  | 1 | 1 | 1 | 1 | 1 |  |  |  |  |
| Calamagrostis neglecta (Ehrh.) P.Gaertn., B.Mey & Scherb. subsp. neglecta | POACEAE | 1 |  |  |  |  | 1 | 1 |  | 1 | 1 | 1 |  |  |  |
| Calamagrostis purpurascens R.Br. subsp. purpurascens | POACEAE | 1 |  | 1 |  |  | 1 | 1 | 1 |  |  |  |  |  |  |
| Callitriche anceps Fernald | PLANTAGINACEAE | 1? |  |  |  |  | 1 | 1 |  |  |  |  |  |  |  |
| Callitriche brutia Petagna | PLANTAGINACEAE | 1 | 1 |  |  |  |  |  |  |  | 1 | 1 | 1 |  |  |
| Callitriche hamulata Kütz. ex W.D.J.Koch | PLANTAGINACEAE | 1? | 1 |  | 1 | 1 |  | 1 |  |  | 1 | 1 | 1 |  |  |
| Callitriche hermaphroditica L. | PLANTAGINACEAE |  | 1 |  | 1 |  | 1 | 1 |  |  | 1 | 1 |  |  |  |
| Callitriche palustris L. | PLANTAGINACEAE |  | 1 |  |  |  | 1 | 1 |  | 1 | 1 |  | 1 |  |  |
| Callitriche platycarpa Kütz. | PLANTAGINACEAE |  |  |  | 1 |  |  |  |  |  | 1 | 1 |  |  |  |
| Callitriche stagnalis Scop. | PLANTAGINACEAE |  | 1 |  | 1 |  |  |  |  |  | 1 | 1 | 1 |  |  |
| Calluna vulgaris (L.) Hull | ERICACEAE |  | 1 |  | 1 |  |  |  |  |  | 1 | 1 | 1 |  |  |
| Caltha palustris L. subsp. radicans (T.F.Forst.) Hook. | RANUNCULACEAE | 1 | 1 |  | 1 | 1 | 1 |  |  | 1 | 1 | 1 | 1? |  |  |
| Campanula rotundifolia L. subsp. gieseckiana (Vest ex Schult.) Witasek | CAMPANULACEAE | 1 |  | 1 |  |  | 1 | 1 |  | 1? | 1 |  |  |  |  |
| Campanula rotundifolia L. subsp. groenlandica (Berlin) Á.Löve & D.Löve | CAMPANULACEAE | 1 |  |  |  |  | 1 | 1 |  |  | 1 | 1? |  |  |  |
| Campanula rotundifolia L. subsp. rotundifolia | CAMPANULACEAE |  | 1 |  | 1 |  |  |  |  |  | 1 | 1 | 1 |  |  |
| Campanula uniflora L. | CAMPANULACEAE |  | 1 | 1 |  |  | 1 | 1 | 1 | 1 | 1 |  |  |  |  |
| Cardamine bellidifolia L. | BRASSICACEAE |  | 1 | 1 |  |  | 1 | 1 | 1 | 1 | 1 |  |  |  |  |
| Cardamine flexuosa With. | BRASSICACEAE |  |  |  | 1 |  |  |  |  |  | 1 | 1 | 1 |  |  |
| Cardamine hirsuta L. | BRASSICACEAE |  | 1 |  | 1 |  |  |  |  |  | 1 | 1 | 1 |  |  |
| Cardamine nymanii Gand. | BRASSICACEAE |  | 1 | 1 | 1 |  | 1 | 1 | 1 | 1 | 1 | 1? |  |  |  |
| Carex adelostoma V.I.Krecz. | CYPERACEAE |  | 1 |  |  |  | 1 |  |  | 1? | 1 |  |  |  |  |
| Carex arctogena Harry Sm. | CYPERACEAE | 1 |  |  |  |  | 1 | 1 |  |  | 1 |  |  |  |  |
| Carex atrata L. s.l. | CYPERACEAE | 1 | 1 |  | 1 |  | 1 | 1 |  | 1 | 1 | 1 | 1 |  |  |
| Carex atrofusca Schkuhr | CYPERACEAE | 1 |  |  |  | 1 | 1 | 1 | 1 | 1 | 1 | 1 | 1 |  |  |
| Carex bicolor Bellardi ex All. | CYPERACEAE | 1? | 1 |  |  |  | 1 | 1 |  | 1 | 1 |  | 1 |  |  |
| Carex bigelowii Torr. ex Schwein. subsp. arctisibirica (Jurtz.) Á.Löve & D.Löve | CYPERACEAE |  |  | 1 |  |  |  |  |  | 1 | 1 |  |  |  |  |
| Carex bigelowii Torr. ex Schwein. subsp. bigelowii | CYPERACEAE | 1 |  |  |  |  | 1 | 1 | 1 |  | 1 |  |  |  |  |
| Carex bigelowii Torr. ex Schwein. subsp. dacica (Heuff.) T.V.Egorova (subsp. rigida (Raf.) W.Schultze-Motel) | CYPERACEAE | 1 | 1 |  | 1 |  |  | 1? |  |  | 1 | 1 | 1 |  |  |
| Carex binervis Sm. | CYPERACEAE |  |  |  | 1 |  |  |  |  |  | 1 | 1 |  |  |  |
| Carex brunnescens (Pers.) Poir. subsp. brunnescens | CYPERACEAE |  | 1 |  |  |  | 1 | 1 |  | 1 | 1 |  | 1 |  |  |
| Carex canescens L. | CYPERACEAE | 1 | 1 |  |  | 1 | 1 | 1 |  | 1 | 1 | 1 | 1 |  |  |
| Carex capillaris L. subsp. capillaris | CYPERACEAE | 1 |  |  | 1 |  | 1? | 1? |  | 1? | 1 | 1 | 1 |  |  |
| Carex capillaris L. subsp. fuscidula (V.I.Krecz. ex T.V.Egorova) Á.Löve & D.Löve | CYPERACEAE |  | 1 | 1 |  |  | 1 | 1 | 1 | 1 | 1? |  |  |  |  |
| Carex capillaris L. var. elongata Olney ex Fernald | CYPERACEAE | 1 |  |  |  |  | 1 | 1 |  |  |  |  |  |  |  |
| Carex capitata L. | CYPERACEAE |  | 1 |  |  |  | 1 | 1? |  | 1 | 1 |  | 1 |  |  |
| Carex caryophyllea Latourr. | CYPERACEAE | 1 | 1 |  |  |  |  |  |  |  | 1 | 1 | 1 |  |  |
| Carex chordorrhiza L. f. | CYPERACEAE | 1 | 1 |  |  |  | 1 | 1 |  | 1 | 1 | 1 | 1 |  |  |
| Carex concolor R.Br. (C. aquatilis Wahlenb. subsp. stans (Drejer) Hultén) | CYPERACEAE | 1 |  | 1 |  |  | 1 | 1 | 1 | 1 | 1 |  |  |  |  |
| Carex deflexa Hornem. | CYPERACEAE | 1 |  |  |  |  | 1 | 1 |  |  |  |  |  |  |  |
| Carex demissa Hornem. | CYPERACEAE |  | 1 |  | 1 |  | 1 | 1 |  |  | 1 | 1 |  |  |  |
| Carex diandra Schrank | CYPERACEAE | 1 | 1 |  |  |  | 1 |  |  | 1 | 1 | 1 | 1 |  |  |
| Carex dioica L. | CYPERACEAE | 1 | 1 |  | 1 |  |  |  |  | 1 | 1 | 1 | 1 |  |  |
| Carex echinata Murray | CYPERACEAE |  | 1 |  | 1 |  | 1 |  |  |  | 1 | 1 | 1 |  |  |
| Carex flacca Schreb. | CYPERACEAE |  | 1 |  | 1 |  |  |  |  |  | 1 | 1 | 1 |  |  |
| Carex flava L. | CYPERACEAE | 1 | 1 |  |  |  | 1 |  |  |  | 1 | 1 | 1 |  |  |
| Carex fuliginosa Schkuhr subsp. misandra (R.Br.) Nyman | CYPERACEAE | 1 |  | 1 |  |  | 1 | 1 |  | 1 | 1 |  | 0 |  | Vicariant taxon in C Europe: subsp. fuliginosa |
| Carex glacialis Mack. | CYPERACEAE |  | 1 | 1 |  |  | 1 | 1 |  | 1 | 1 |  |  |  |  |
| Carex glareosa Schkuhr ex Wahlenb. | CYPERACEAE |  | 1 | 1 |  |  | 1 | 1 | 1 | 1 | 1 |  |  |  |  |
| Carex heleonastes Ehrh. ex L. f. | CYPERACEAE | 1 | 1 |  |  |  | 1 |  |  | 1? | 1 |  | 1 |  |  |
| Carex hostiana DC. | CYPERACEAE |  |  |  | 1 |  |  |  |  |  | 1 | 1 | 1 |  |  |
| Carex krausei Boeck. | CYPERACEAE |  | 1 | 1 |  |  | 1 | 1 | 1 |  |  |  |  |  |  |
| Carex lachenalii Schkuhr | CYPERACEAE | 1 | 1 | 1 |  | 1 | 1 | 1 |  | 1 | 1 | 1 | 1 |  |  |
| Carex leporina L. | CYPERACEAE |  |  |  | 1 |  |  |  |  |  | 1 | 1 | 1 |  |  |
| Carex lidii Hadač | CYPERACEAE | 1 |  | 1 |  |  |  |  |  |  |  |  |  |  |  |
| Carex limosa L. | CYPERACEAE | 1 | 1 |  |  |  | 1 |  |  | 1 | 1 | 1 | 1 |  |  |
| Carex livida (Wahlenb.) Willd. | CYPERACEAE |  | 1 |  |  |  | 1 |  |  |  | 1 |  |  |  |  |
| Carex lyngbyei Hornem. subsp. lyngbyei | CYPERACEAE |  | 1 |  | 1 |  |  | 1 |  |  |  |  |  |  | Endemic to Faeroes, Iceland, and Greenland. The other subspecies, subsp. cryptocarpa (C.A.Mey.) Hultén, is Beringian |
| Carex mackenziei V.I.Krecz. | CYPERACEAE |  | 1 |  |  |  | 1 | 1 |  | 1 | 1 |  |  |  |  |
| Carex macloviana d'Urv. | CYPERACEAE |  | 1 |  |  |  | 1 | 1 |  |  | 1 |  |  |  |  |
| Carex marina Dewey subsp. pseudolagopina (T.J.Sørensen) Böcher | CYPERACEAE | 1 |  | 1 |  |  | 0 | 1 | 1 | 0 |  |  |  |  | Vicariant taxon in E Canada & N Urals: subsp. marina (nearly circumpolar, also in W Greenland) |
| Carex maritima Gunnerus | CYPERACEAE | 1 | 1 | 1 | 1 | 1 | 1 | 1 | 1 | 1 | 1 | 1 | 1 |  |  |
| Carex microglochin Wahlenb. | CYPERACEAE | 1 | 1 |  |  |  | 1 | 1 |  |  | 1 | 1 | 1 |  |  |
| Carex miliaris Michx. | CYPERACEAE | 1 |  |  |  |  | 1 | 1 |  |  |  |  |  |  |  |
| Carex nardina Fr. subsp. hepburnii (Boott) Á.Löve, D.Löve & B.M.Kapoor | CYPERACEAE | 1 |  | 1 |  |  | 1 | 1 | 1 |  |  |  |  |  |  |
| Carex nardina Fr. subsp. nardina | CYPERACEAE |  | 1 |  |  |  |  |  |  |  | 1 |  |  |  |  |
| Carex nigra (L.) Reichard. | CYPERACEAE | 1? | 1 |  | 1 |  | 1 | 1 |  |  | 1 | 1 | 1 |  |  |
| Carex norvegica Retz. subsp. inserrulata Kalela | CYPERACEAE | 1 |  |  |  |  | 1 | 1 |  |  |  |  |  |  |  |
| Carex norvegica Retz. subsp. norvegica | CYPERACEAE | 1 | 1 |  |  |  |  | 1 |  |  | 1 | 1 | 1 |  |  |
| Carex pallescens L. var. pallescens | CYPERACEAE | 1 | 1 |  |  |  | 0 |  |  |  | 1 | 1 | 1 |  | Vicariant taxon in E Canada: var. neogaea Fernald |
| Carex panicea L. | CYPERACEAE |  | 1 |  | 1 |  |  | 1 |  |  | 1 | 1 | 1 |  |  |
| Carex parallella (Laest.) Sommerf. | CYPERACEAE | 1 |  | 1 |  |  |  |  |  | 1 | 1 |  |  |  |  |
| Carex paupercula Michx. (C. magellanica subsp. irrigua (Wahlenb.) Hiitonen) | CYPERACEAE | 1 | 1 |  |  |  | 1 | 1 |  | 1 | 1 | 1 | 1 |  |  |
| Carex pilulifera L. | CYPERACEAE |  | 1 |  | 1 |  |  |  |  |  | 1 | 1 | 1 |  |  |
| Carex praticola Rydb. | CYPERACEAE | 1 |  |  |  |  | 1 | 1 |  |  |  |  |  |  |  |
| Carex pulicaris L. | CYPERACEAE |  | 1 |  | 1 |  |  |  |  |  | 1 | 1 | 1 |  |  |
| Carex ramenskii Kom. subsp. 'nova' | CYPERACEAE |  | 1 |  |  |  |  | 1 |  |  |  |  |  |  | Undescribed subspecies (or nothosubspecies), endemic to Iceland and Greenland. The nominate race is Beringian |
| Carex rariflora (Wahlenb.) Sm. | CYPERACEAE |  | 1 |  |  |  | 1 | 1 |  | 1 | 1 | 1 |  |  |  |
| Carex recta Booth/vacillans Drejer | CYPERACEAE |  |  |  | 1 |  | 1 |  |  |  | 1 | 1 |  |  | Complex of one or more hybridogeneous species |
| Carex rostrata Stokes | CYPERACEAE | 1 | 1 |  | 1 |  | 1 | 1 |  | 1 | 1 | 1 | 1 |  |  |
| Carex rufina Drejer | CYPERACEAE |  | 1 |  |  |  | 1 | 1 |  |  | 1 |  |  |  |  |
| Carex rupestris All. | CYPERACEAE | 1 | 1 | 1 |  | 1 | 1 | 1 | 1 | 1 | 1 | 1 | 1 |  |  |
| Carex saxatilis L. subsp. laxa (Trautv.) Kalela | CYPERACEAE | 1 |  | 1 |  |  | 1 | 1 | 1 | 1 | 1 |  |  |  |  |
| Carex saxatilis L. subsp. saxatilis | CYPERACEAE |  | 1 |  | 1 |  |  |  |  |  | 1 | 1 |  |  |  |
| Carex scirpoidea Michx. | CYPERACEAE | 1 |  |  |  |  | 1 | 1 | 1 |  | 1 |  |  |  |  |
| Carex stylosa C.A.Mey. var. nigritella (Drejer) Fernald | CYPERACEAE | 1 |  |  |  |  | 1 | 1 |  |  | 1 |  |  |  |  |
| Carex subspathacea Wormsk. ex Hornem. | CYPERACEAE |  | 1 | 1 |  |  | 1 | 1 |  | 1 | 1 |  |  |  |  |
| Carex supina Willd. ex Wahlenb. subsp. spaniocarpa (Steud.) Hultén | CYPERACEAE | 1 |  |  |  |  | 1 | 1 | 1 |  |  |  | 0 |  | Vicariant taxon in C Europe and W Asia: subsp. supina |
| Carex ursina Dewey | CYPERACEAE | 1 |  | 1 |  |  | 1 | 1 | 1 | 1 |  |  |  |  |  |
| Carex vaginata Tausch subsp. algida (Turcz. ex V.I.Krecz.) Elven ined. | CYPERACEAE | 1 |  |  |  |  | 1 |  |  | 1 | 1 |  |  |  |  |
| Carex vaginata Tausch subsp. vaginata | CYPERACEAE | 1 | 1 |  |  |  |  |  |  |  | 1 | 1 | 1 |  |  |
| Carex viridula Michx. var. viridula | CYPERACEAE | 1 | 1 |  |  |  | 1 | 1 |  |  | 1 | 1 | 1 |  |  |
| Cassiope tetragona (L.) D.Don | ERICACEAE | 1 |  | 1 |  |  | 1 | 1 | 1 | 1 | 1 |  |  |  |  |
| Catabrosa aquatica (L.) P.Beauv. var. aquatica | POACEAE |  | 1 |  | 1 | 1 | 0 | 1 |  |  | 1 | 1 | 1 |  | Vicariant taxon in E Canada: var. laurentiana Fernald |
| Cerastium alpinum L. subsp. alpinum | CARYOPHYLLACEAE |  | 1 |  |  |  |  |  |  | 1? | 1 |  |  |  |  |
| Cerastium alpinum L. subsp. lanatum (Lam.) Ces. | CARYOPHYLLACEAE | 1 | 1 |  |  |  | 1 | 1 |  |  | 1 | 1 | 1 |  |  |
| Cerastium alpinum L. subsp. 'nova' | CARYOPHYLLACEAE | 1 |  | 1 |  |  | 1 | 1 | 1 |  |  |  |  |  | Undescribed arctic race |
| Cerastium alpinum L. var. glabrum Wahlenb. (C. glabratum Hartm.) | CARYOPHYLLACEAE |  | 1 |  |  |  |  |  |  |  | 1 |  |  |  |  |
| Cerastium arcticum Lange | CARYOPHYLLACEAE | 1 |  | 1 |  |  | 1 | 1 | 1 |  |  |  |  |  |  |
| Cerastium cerastoides (L.) Britton | CARYOPHYLLACEAE | 1 | 1 | 1 | 1 | 1 | 1 | 1 |  | 1 | 1 | 1 | 1 |  |  |
| Cerastium diffusum Pers. | CARYOPHYLLACEAE |  |  |  | 1 |  |  |  |  |  | 1 | 1 |  |  |  |
| Cerastium fontanum Baumg. subsp. fontanum | CARYOPHYLLACEAE |  | 1 |  | 1 |  |  | 1 |  | 1? | 1 |  | 1 |  |  |
| Cerastium nigrescens (H.C.Watson) Edmondston ex H.C.Watson | CARYOPHYLLACEAE |  | 1 |  | 1 |  |  |  |  |  | 1 | 1 |  |  |  |
| Cerastium regelii Ostenf. subsp. caespitosum (Malmgren) Tolm. | CARYOPHYLLACEAE | 1 |  | 1 |  |  | 1 |  | 1 | 1 |  |  |  |  |  |
| Chamaepericlymenum suecicum (L.) Graebn. | CORNACEAE |  | 1 |  | 1 |  | 1 | 1 |  | 1 | 1 | 1 |  |  |  |
| Chamerion angustifolium (L.) Holub | ONAGRACEAE | 1 | 1 |  | 1 |  | 1 | 1 |  | 1 | 1 | 1 | 1 |  |  |
| Chamerion latifolium (L.) Holub | ONAGRACEAE |  | 1 |  |  |  | 1 | 1 | 1 | 1 |  |  |  |  |  |
| Chamerion x intermedium (Wormsk.) | ONAGRACEAE | 1 |  |  |  |  | 1 | 1 |  |  |  |  |  |  |  |
| Chrysosplenium tetrandrum (N.Lund) Th.Fr. | SAXIFRAGACEAE | 1 |  | 1 |  |  | 1 |  |  | 1 | 1 |  |  |  |  |
| Cirsium palustre (L.) Coss. ex Scop. | ASTERACEAE |  |  |  | 1 |  |  |  |  |  | 1 | 1 | 1 |  |  |
| Cochlearia groenlandica L. | BRASSICACEAE |  | 1 | 1 |  |  | 1 | 1 | 1 | 1 |  |  |  |  |  |
| Cochlearia officinalis L. subsp. integrifolia (Hartm.) Nordal & Stabbetorp | BRASSICACEAE |  |  |  | 1 |  |  |  |  |  | 1 |  |  |  |  |
| Cochlearia officinalis L. subsp. islandica (Pobed.) Nordal & Bjorå ined. | BRASSICACEAE |  | 1 |  |  |  |  |  |  |  |  |  |  | End | Endemic to Iceland; taxonomically unclear |
| Cochlearia officinalis L. subsp. officinalis | BRASSICACEAE |  |  |  | 1 |  |  |  |  |  | 1 | 1 |  |  |  |
| Coeloglossum viride (L.) Hartm. (Dactylorhiza viridis (L.) R.M.Bateman, Pridgeon & M.W.Chase) | ORCHIDACEAE | 1 | 1 |  | 1 |  | 1 |  |  | 1 | 1 | 1 | 1 |  |  |
| Comarum palustre L. | ROSACEAE | 1 | 1 |  | 1 |  | 1 | 1 |  | 1 | 1 | 1 | 1 |  |  |
| Comastoma tenellum (Rottb.) Toyok. | GENTIANACEAE | 1? | 1 | 1 |  |  | 1 | 1 |  | 1 | 1 |  | 1 |  |  |
| Conopodium majus (Gouan) Loret | APIACEAE |  |  |  | 1 |  |  |  |  |  | 1 | 1 |  |  |  |
| Coptidium lapponicum (L.) Tzvelev | RANUNCULACEAE |  |  | 1 |  |  | 1 | 1 |  | 1 | 1 |  |  |  |  |
| Coptidium pallasii (Schltdl.) Tzvelev | RANUNCULACEAE |  |  | 1 |  |  | 1 |  |  | 1 | 1 |  |  |  |  |
| Coptidium spitsbergense (Hadač) Elven | RANUNCULACEAE |  |  | 1 |  |  | 1 |  |  | 1 |  |  |  |  |  |
| Coptis trifolia (L.) Salisb. | RANUNCULACEAE | 1 |  |  |  | 1 | 1 | 1 |  |  |  |  |  |  |  |
| Corallorhiza trifida Châtel. | ORCHIDACEAE | 1 | 1 |  |  | 1 | 1 | 1 |  | 1 | 1 | 1 | 1 |  |  |
| Crassula aquatica (L.) Schönland | CRASSULACEAE |  | 1 |  |  |  | 1 |  |  |  | 1 | 1 |  |  |  |
| Crepis paludosa (L.) Moench | ASTERACEAE | 1 | 1 |  |  |  |  |  |  | 1 | 1 | 1 | 1 |  |  |
| Cryptogramma crispa (L.) R.Br. ex Hook. | ADIANTACEAE | 1 | 1 |  |  |  |  |  |  | 1 | 1 | 1 | 1 |  |  |
| Cystopteris fragilis (L.) Bernh. | WOODSIACEAE | 1 | 1 | 1 | 1 | 1 | 1 | 1 | 1 | 1 | 1 | 1 | 1 |  |  |
| Dactylorhiza maculata (L.) Soó | ORCHIDACEAE | 1 | 1 |  | 1 |  |  |  |  | 1 | 1 | 1 | 1 |  |  |
| Dactylorhiza majalis (Rchb.) P.F.Hunt & Summerh. subsp. purpurella (T.Stephenson & T.A.Stephenson) D.M.Moore & Soó | ORCHIDACEAE |  |  |  | 1 |  |  |  |  |  | 1 | 1 |  |  |  |
| Danthonia decumbens (L.) DC. | POACEAE |  | 1 |  | 1 |  |  |  |  |  | 1 | 1 | 1 |  |  |
| Deschampsia alpina (L.) Roem. & Schult. | POACEAE |  | 1 | 1 | 1 |  | 1 | 1 |  | 1 | 1 | 1 |  |  |  |
| Deschampsia brevifolia R.Br. | POACEAE | 1 |  |  |  |  | 1 | 1 | 1 |  |  |  |  |  |  |
| Deschampsia cespitosa (L.) P.Beauv. | POACEAE |  | 1 |  | 1 |  |  |  |  |  | 1 | 1 | 1 |  |  |
| Deschampsia sukatschewii (Popl.) Roshev. subsp. borealis (Trautv.) Tzvelev | POACEAE | 1 |  | 1 |  |  | 1 | 1 | 1 | 1 |  |  |  |  |  |
| Diapensia lapponica L. | DIAPENSIACEAE |  | 1 |  |  |  | 1 | 1 | 1 | 1 | 1 | 1 |  |  |  |
| Diphasiastrum alpinum (L.) Holub | LYCOPODIACEAE | 1 | 1 |  | 1 | 1 | 1 | 1 |  | 1 | 1 | 1 | 1 |  |  |
| Draba alpina L. | BRASSICACEAE | 1 |  | 1 |  |  | 1 | 1 | 1 | 1? | 1 |  |  |  |  |
| Draba arctica J.Vahl subsp. arctica | BRASSICACEAE | 1 |  | 1 |  |  | 1 | 1 | 1 |  |  |  |  |  |  |
| Draba arctica J.Vahl subsp. ostenfeldii (Ekman) Böcher ex Kartesz & S.K.Gandhi | BRASSICACEAE | 1 |  |  |  |  | 1? | 1 | 1 |  |  |  |  |  |  |
| Draba arctogena (Ekman) Ekman | BRASSICACEAE | 1 |  |  |  |  | 1 | 1 | 1 |  |  |  |  |  |  |
| Draba aurea Vahl ex Hornem. | BRASSICACEAE | 1 |  |  |  |  | 1 | 1 |  |  |  |  |  |  |  |
| Draba cana Rydb. | BRASSICACEAE | 1 |  |  |  |  | 1 | 1 |  |  |  |  |  |  |  |
| Draba cinerea Adams | BRASSICACEAE | 1 |  |  |  |  | 1 | 1 | 1 | 1 | 1 |  |  |  |  |
| Draba corymbosa R.Br. ex DC. | BRASSICACEAE | 1 |  | 1 |  |  | 1 | 1 | 1 | 1 |  |  |  |  |  |
| Draba crassifolia Graham | BRASSICACEAE | 1 |  |  |  |  | 1 | 1 |  |  | 1 |  |  |  |  |
| Draba fladnizensis Wulfen | BRASSICACEAE | 1 |  | 1 |  |  | 1 | 1 | 1 | 1 | 1 |  | 1 |  |  |
| Draba glabella Pursh | BRASSICACEAE | 1 |  | 1 |  |  | 1 | 1 | 1 | 1 | 1 |  |  |  |  |
| Draba incana L. | BRASSICACEAE | 1 | 1 |  | 1 |  | 1 | 1 |  |  | 1 | 1 | 1 |  |  |
| Draba lactea Adams | BRASSICACEAE | 1 |  | 1 |  |  | 1 | 1 | 1 | 1 | 1 |  |  |  |  |
| Draba micropetala Hook. | BRASSICACEAE | 1 |  | 1 |  |  | 1 | 1 | 1 | 1 |  |  |  |  |  |
| Draba nivalis Lilj. | BRASSICACEAE |  | 1 | 1 |  |  | 1 | 1 | 1 | 1 | 1 |  |  |  |  |
| Draba norvegica Gunnerus (D. rupestris R.Br.) | BRASSICACEAE |  | 1 | 1 | 1 |  | 1 | 1 |  | 1 | 1 | 1 |  |  |  |
| Draba oblongata R.Br. ex DC. | BRASSICACEAE | 1 |  |  |  |  | 1 | 1 | 1 |  |  |  |  |  |  |
| Draba oxycarpa Sommerf. | BRASSICACEAE |  | 1 | 1 |  |  |  |  |  |  | 1 |  |  |  |  |
| Draba pauciflora R.Br. | BRASSICACEAE | 1 |  | 1 |  |  | 1 | 1 | 1 | 1 |  |  |  |  |  |
| Draba sibirica (Pall.) Thell. subsp. arctica Böcher | BRASSICACEAE | 1 |  |  |  |  |  |  |  | 1 |  |  |  |  |  |
| Draba subcapitata Simmons | BRASSICACEAE | 1 |  | 1 |  |  | 1 | 1 | 1 | 1 | 1 |  |  |  |  |
| Draba verna L. | BRASSICACEAE | 1 | 1 |  |  |  |  |  |  |  | 1 | 1 | 1 |  |  |
| Drosera rotundifolia L. | DROSERACEAE | 1? | 1 |  | 1 |  | 1 | 1 |  |  | 1 | 1 | 1 |  |  |
| Dryas integrifolia Vahl | ROSACEAE | 1 |  |  |  |  | 1 | 1 | 1 |  |  |  |  |  |  |
| Dryas octopetala L. | ROSACEAE | 1 | 1 | 1 | 1 | 1 |  |  |  | 1 | 1 | 1 | 1 |  |  |
| Dryopteris dilatata (Hoffm.) A.Gray | DRYOPTERIDACEAE |  |  |  | 1 |  |  |  |  |  | 1 | 1 | 1 |  |  |
| Dryopteris expansa (C.Presl) Fraser-Jenk. & Jermy | DRYOPTERIDACEAE | 1 | 1 |  | 1 |  | 1 | 1 |  | 1 | 1 | 1 | 1 |  |  |
| Dryopteris filix-mas (L.) Schott | DRYOPTERIDACEAE | 1 | 1 |  | 1 |  | 1 | 1 |  | 1 | 1 | 1 | 1 |  |  |
| Dryopteris fragrans (L.) Schott | DRYOPTERIDACEAE | 1 |  |  |  |  | 1 | 1 | 1 | 1 | 1 |  |  |  |  |
| Dupontia fisheri R.Br. subsp. fisheri | POACEAE |  |  | 1 |  |  | 1 | 1 | 1 | 1 |  |  |  |  |  |
| Dupontia fisheri R.Br. subsp. psilosantha (Rupr.) Hultén | POACEAE | 1 |  | 1 |  |  | 1 | 1 |  | 1 |  |  |  |  |  |
| Eleocharis acicularis (L.) Roem. & Schult. | CYPERACEAE | 1 | 1 |  |  |  | 1 | 1 |  |  | 1 | 1 | 1 |  |  |
| Eleocharis multicaulis (Sm.) Desv. | CYPERACEAE |  |  |  | 1 |  |  |  |  |  | 1 | 1 |  |  |  |
| Eleocharis palustris (L.) Roem. & Schult. s.l. | CYPERACEAE | 1? | 1 |  | 1 |  | 1 | 1 |  |  | 1 | 1 | 1 |  | Several unresolved races or species |
| Eleocharis quinqueflora (Hartmann) O.Schwarz subsp. fernaldii (Svenson) Hultén | CYPERACEAE | 1 |  |  |  |  | 1 | 1 |  |  |  |  |  |  |  |
| Eleocharis quinqueflora (Hartmann) O.Schwarz subsp. quinqueflora | CYPERACEAE | 1 | 1 |  | 1 |  |  |  |  |  | 1 | 1 | 1 |  |  |
| Eleocharis uniglumis (Link) Schult. | CYPERACEAE | 1? | 1 |  | 1 |  | 1 | 1 |  |  | 1 | 1 | 1 |  |  |
| Elymus alaskanus (Scribn. & Merr.) Á.Löve subsp. borealis (Turcz.) Á.Löve & D.Löve | POACEAE |  | 1 |  |  |  |  |  |  | 1 | 1 |  |  |  |  |
| Elymus alaskanus (Scribn. & Merr.) Á.Löve subsp. hyperarcticus (Polunin) Á.Löve & D.Löve | POACEAE | 1 |  |  |  |  | 1 | 1 | 1 |  |  |  |  |  |  |
| Elymus alopex B.Salomon | POACEAE |  | 1 |  |  |  |  |  |  |  |  | 0 | 0 | End | Possibly the same as E. caninus (L.) L. in Europe |
| Elymus trachycaulus (Link) Gould ex Shinners subsp. virescens (Lange) Á.Löve & D.Löve | POACEAE | 1? |  |  |  |  | 0 | 1 |  |  |  |  |  |  | Vicariant taxa in E Canada: several other subspecies |
| Elytrigia juncea (L.) Nevski | POACEAE |  |  |  | 1 |  |  |  |  |  | 1 | 1 |  |  |  |
| Empetrum nigrum L. 2x | ERICACEAE |  | 1 |  | 1 |  | 1 |  |  |  | 1 | 1 | 1 |  | Diploid morphs |
| Empetrum nigrum L. 4x (E. hermaphroditum Hagerup p.p.) | ERICACEAE | 1 | 1 | 1 | 1 | 1 | 1 | 1 | 1 | 1 | 1 | 1 | 1 |  | Tetraploid morphs |
| Epilobium alsinifolium Vill. | ONAGRACEAE | 1? | 1 |  | 1 |  |  | 1? |  |  | 1 | 1 | 1 |  |  |
| Epilobium anagallidifolium Lam. | ONAGRACEAE | 1 | 1 |  | 1 | 1 | 1 | 1 |  | 1 | 1 | 1 | 1 |  |  |
| Epilobium arcticum Sam. | ONAGRACEAE | 1 |  |  |  |  | 1 | 1 | 1 | 1 |  |  |  |  |  |
| Epilobium collinum C.C.Gmel. | ONAGRACEAE |  | 1 |  |  |  |  |  |  |  | 1 |  | 1 |  |  |
| Epilobium hornemannii Rchb. | ONAGRACEAE |  | 1 |  |  |  | 1 | 1 |  | 1 | 1 |  |  |  |  |
| Epilobium lactiflorum Hausskn. | ONAGRACEAE |  | 1 |  | 1 |  | 1 | 1 |  |  | 1 |  |  |  |  |
| Epilobium montanum L. | ONAGRACEAE |  |  |  | 1 |  |  |  |  |  | 1 | 1 | 1 |  |  |
| Epilobium palustre L. | ONAGRACEAE | 1 | 1 |  | 1 |  | 1 | 1 |  | 1 | 1 | 1 | 1 |  |  |
| Equisetum arvense L. subsp. alpestre (Wahlenb.) Schönswetter & Elven | EQUISETACEAE | 1 | 1 | 1 |  | 1 | 1 | 1 | 1 | 1 | 1 | 1? | 1 |  |  |
| Equisetum arvense L. subsp. arvense | EQUISETACEAE | 1 | 1 |  | 1 |  | 1 | 1? |  | 1 | 1 | 1 | 1 |  |  |
| Equisetum fluviatile L. | EQUISETACEAE | 1 | 1 |  | 1 |  | 1 |  |  | 1 | 1 | 1 | 1 |  |  |
| Equisetum hyemale L. subsp. hyemale | EQUISETACEAE | 1 | 1 |  | 1 |  | 0 |  |  | 1 | 1 | 1 | 1 |  | Vicariant taxon in E Canada: subsp. affine (Engelm.) Calder & R.L.Taylor |
| Equisetum mackaii (Newman) Brichan (E. trachyodon A.Braun) W.D.J.Koch) | EQUISETACEAE |  | 1 |  |  |  | 1 | 1 |  |  | 1 | 1 |  |  |  |
| Equisetum palustre L. | EQUISETACEAE | 1 | 1 |  | 1 |  | 1 |  |  | 1 | 1 | 1 | 1 |  |  |
| Equisetum pratense Ehrh. | EQUISETACEAE | 1 | 1 |  | 1 |  | 1 |  |  | 1 | 1 | 1 | 1 |  |  |
| Equisetum scirpoides Michx. | EQUISETACEAE |  |  | 1 |  |  | 1 | 1 |  | 1 | 1 |  |  |  |  |
| Equisetum sylvaticum L. | EQUISETACEAE | 1 | 1 |  | 1 |  | 1 | 1 |  | 1 | 1 | 1 | 1 |  |  |
| Equisetum variegatum Schleich. ex F.Weber & D.Mohr | EQUISETACEAE | 1 | 1 | 1 | 1 | 1 | 1 | 1 | 1 | 1 | 1 | 1 | 1 |  |  |
| Erica cinerea L. | ERICACEAE |  |  |  | 1 |  |  |  |  |  | 1 | 1 |  |  |  |
| Erigeron alpiniformis Cronquist | ASTERACEAE | 1? |  |  |  |  |  | 1 |  |  |  |  |  |  | Endemic to Greenland |
| Erigeron borealis (Vierh.) Simmons | ASTERACEAE |  | 1 |  |  |  |  |  |  | 1 | 1 | 1 | 0 |  | Vicariant taxa in C European mts: E. neglectus A.Kern. etc. |
| Erigeron compositus Pursh | ASTERACEAE | 1 |  |  |  |  | 1 | 1 | 1 |  |  |  |  |  |  |
| Erigeron eriocephalus J.Vahl | ASTERACEAE | 1 |  | 1 |  |  | 1 | 1 | 1 | 1 | 1 |  |  |  |  |
| Erigeron humilis Graham | ASTERACEAE |  | 1 | 1 |  |  | 1 | 1 |  |  | 1 |  |  |  |  |
| Erigeron uniflorus L. | ASTERACEAE |  | 1 |  |  |  |  |  |  |  | 1 |  | 1 |  |  |
| Eriophorum angustifolium Honck. | CYPERACEAE | 1 | 1 |  | 1 |  | 1 | 1 |  | 1 | 1 | 1 | 1 |  |  |
| Eriophorum callitrix Cham. ex C.A.Mey. | CYPERACEAE | 1 |  |  |  |  | 1 |  | 1 | 1 |  |  |  |  |  |
| Eriophorum scheuchzeri Hoppe subsp. arcticum M.S.Novos. | CYPERACEAE | 1 |  | 1 |  |  | 1 | 1 | 1 | 1 |  |  |  |  |  |
| Eriophorum scheuchzeri Hoppe subsp. scheuchzeri | CYPERACEAE |  | 1 |  |  |  | 1 | 1 |  | 1 | 1 |  | 1 |  |  |
| Eriophorum triste (Th.Fr.) Hadač & Á.Löve | CYPERACEAE | 1 |  | 1 |  |  | 1 |  | 1 | 1 |  |  |  |  |  |
| Eriophorum vaginatum L. subsp. vaginatum | CYPERACEAE |  |  |  | 1 |  | 0 | 0 |  | 1 | 1 | 1 | 1 |  | Vicariant taxon in E Canada & W Greenland: subsp. spissum (Fernald) Hultén |
| Eriophorum x sorenseni Raymond | CYPERACEAE | 1 |  | 1 |  |  | 1 |  |  |  |  |  |  |  |  |
| Erysimum strictum P.Gaertn., B.Mey. & Scherb. | BRASSICACEAE |  | 1 |  |  |  |  |  |  |  | 1 |  | 1 |  |  |
| Euphrasia arctica Lange ex Rostr. | OROBANCHACEAE |  | 1 |  | 1 |  |  |  |  |  | 1 | 1 |  |  |  |
| Euphrasia atropurpurea (Rostr.) Ostenf. | OROBANCHACEAE |  |  |  | 1 |  |  |  |  |  |  |  |  | End |  |
| Euphrasia calida Yeo | OROBANCHACEAE |  | 1 |  |  |  |  |  |  |  |  |  |  | End |  |
| Euphrasia confusa Pugsl. | OROBANCHACEAE |  |  |  | 1 |  |  |  |  |  |  | 1 |  |  |  |
| Euphrasia foulaënsis Towns. ex Wettst. | OROBANCHACEAE |  |  |  | 1 |  |  |  |  |  |  | 1 |  |  |  |
| Euphrasia frigida Pugsley | OROBANCHACEAE | 1 |  |  |  |  | 1? | 1 |  |  |  |  |  |  |  |
| Euphrasia micrantha Rchb. | OROBANCHACEAE |  |  |  | 1 |  |  |  |  |  | 1 | 1 | 1 |  |  |
| Euphrasia ostenfeldii (Pugsley) Yeo | OROBANCHACEAE |  |  |  | 1 |  |  |  |  |  |  | 1 |  |  |  |
| Euphrasia scottica Wettst. | OROBANCHACEAE |  |  |  | 1 |  |  |  |  |  | 1 | 1 |  |  |  |
| Euphrasia wettsteinii G.L.Gusarova | OROBANCHACEAE |  | 1 | 1 | 1 |  | 1 | 1 | 1 |  | 1 | 1 |  |  |  |
| Eutrema edwardsii R.Br. | BRASSICACEAE | 1 |  | 1 |  |  | 1 | 1 | 1 | 1 |  |  |  |  |  |
| Festuca baffinensis Polunin | POACEAE | 1 |  | 1 |  |  | 1 | 1 | 1 | 1 |  |  |  |  |  |
| Festuca brachyphylla Schult. | POACEAE | 1 |  | 1 |  |  | 1 | 1 | 1 | 1 |  |  |  |  |  |
| Festuca edlundiae S.G.Aiken, Consaul & Lefk. | POACEAE | 1 |  | 1 |  |  | 1 |  | 1 |  |  |  |  |  |  |
| Festuca frederikseniae E.B.Alexeev | POACEAE | 1 |  | 1? |  |  | 1 | 1 |  |  |  |  |  |  | Identity of Svalbard plant uncertain |
| Festuca groenlandica | POACEAE | 1? |  |  |  |  |  | 1 |  |  |  |  |  |  | Endemic to Greenland (W & E) |
| Festuca hyperborea Holmen ex Fred. | POACEAE | 1 |  | 1 |  |  | 1 | 1 | 1 | 1 |  |  |  |  |  |
| Festuca ovina L. subsp. ovina | POACEAE | 1 |  | 1 | 1 |  |  |  |  | 1 | 1 | 1 | 1 |  |  |
| Festuca rubra L. subsp. richardsonii (Hook.) Hultén | POACEAE |  | 1 | 1 |  |  | 1 | 1 |  | 1 | 1 |  |  |  |  |
| Festuca rubra L. subsp. rubra | POACEAE | 1 | 1 |  | 1 | 1 | 1 | 1 |  | 1 | 1 | 1 | 1 |  |  |
| Festuca vivipara (L.) Sm. | POACEAE |  | 1 | 1 | 1 | 1 |  | 1 |  |  | 1 | 1 |  |  |  |
| Festuca viviparoidea Krajina ex Pavlick | POACEAE | 1 |  | 1 |  |  |  |  | 1 | 1 |  |  |  |  |  |
| Filipendula ulmaria (L.) Maxim. | ROSACEAE |  | 1 |  | 1 |  |  |  |  |  | 1 | 1 | 1 |  |  |
| Fragaria vesca L. subsp. vesca | ROSACEAE | 1 | 1 |  |  |  | 0 |  |  |  | 1 | 1 | 1 |  | Vicariant taxon in E Canada: subsp. americana (Porter) Staudt |
| Galeopsis tetrahit L. | LAMIACEAE |  |  |  | 1 |  |  |  |  |  | 1 | 1 | 1 |  |  |
| Galium boreale L. | RUBIACEAE | 1 | 1 |  |  |  | 1 | 1 |  | 1 | 1 | 1 | 1 |  |  |
| Galium normanii O.C.Dahl | RUBIACEAE |  | 1 |  |  |  |  |  |  |  | 1 |  |  |  |  |
| Galium palustre L. | RUBIACEAE |  | 1 |  | 1 |  | 1 |  |  |  | 1 | 1 | 1 |  |  |
| Galium saxatile L. | RUBIACEAE |  |  |  | 1 |  |  |  |  |  | 1 | 1 | 1 |  |  |
| Galium trifidum L. | RUBIACEAE |  | 1 |  |  |  | 1 | 1 |  |  | 1 |  |  |  |  |
| Galium triflorum Michx. | RUBIACEAE | 1 |  |  |  |  | 1 | 1 |  |  | 1 |  | 1 |  |  |
| Galium uliginosum L. | RUBIACEAE | 1 | 1 |  |  |  |  |  |  | 1 | 1 | 1 | 1 |  |  |
| Gentiana nivalis L. | GENTIANACEAE | 1 | 1 |  |  |  | 1 | 1 |  |  | 1 | 1 | 1 |  |  |
| Gentianella amarella (L.) Börner subsp. acuta (Michx.) J.M.Gillett | GENTIANACEAE | 1 |  |  |  |  | 1 | 1 |  |  |  |  |  |  |  |
| Gentianella amarella (L.) Börner subsp. septentrionalis (Druce) N.M.Pritch. | GENTIANACEAE |  | 1 |  |  |  |  |  |  |  | 1 | 1 | 0 |  | Very rare in SW Scandinavia; subsp. amarella vicariant in major parts of Europe, incl. Fennoscandia |
| Gentianella aurea (L.) Harry Sm. | GENTIANACEAE |  | 1 |  |  |  |  | 1 |  |  | 1 |  |  |  |  |
| Gentianella campestris (L.) Börner | GENTIANACEAE |  | 1 |  | 1 |  |  |  |  |  | 1 | 1 | 1 |  |  |
| Gentianopsis detonsa (Rottb.) Ma | GENTIANACEAE |  | 1 |  |  |  |  | 1 |  | 1? | 1 |  |  |  |  |
| Geranium sylvaticum L. | GERANIACEAE | 1 | 1 |  | 1 |  |  | 1 |  | 1 | 1 | 1 | 1 |  |  |
| Geum rivale L. | ROSACEAE | 1 | 1 |  | 1 |  | 1 | 1 |  | 1 | 1 | 1 | 1 |  |  |
| Glyceria fluitans (L.) R.Br. | POACEAE |  | 1 |  | 1 |  |  |  |  |  | 1 | 1 | 1 |  |  |
| Gnaphalium uliginosum L. | ASTERACEAE | 1 | 1 |  |  |  | 1 | 1 |  |  | 1 | 1 | 1 |  |  |
| Gymnocarpium dryopteris (L.) Newman | WOODSIACEAE | 1 | 1 |  | 1 |  | 1 | 1 |  | 1 | 1 | 1 | 1 |  |  |
| Hammarbya paludosa (L.) Kuntze | ORCHIDACEAE |  |  |  | 1 |  |  |  |  |  | 1 | 1 | 1 |  |  |
| Harrimanella hypnoides (L.) Coville | ERICACEAE |  | 1 | 1 |  |  | 1 | 1 |  | 1 | 1 |  |  |  |  |
| Hieracium sect. Alpina | ASTERACEAE | 1 | 1 |  | 1 |  |  | 1 |  | 1 | 1 | 1 | 1 |  |  |
| Hieracium sect. Caesia | ASTERACEAE | 1 | 1 |  | 1? | 1 |  |  |  |  | 1 | 1 | 1? |  |  |
| Hieracium sect. Cerinthoidea | ASTERACEAE |  | 1 |  | 1 |  |  |  |  |  | 1 | 1 | 1 |  |  |
| Hieracium sect. Dovrensia (sect. Alpestria) | ASTERACEAE |  | 1 |  | 1 |  |  | 1 |  |  | 1 | 1 |  |  |  |
| Hieracium sect. Hieracium | ASTERACEAE | 1 | 1 |  | 1? |  |  |  |  | 1 | 1 | 1 | 1 |  |  |
| Hieracium sect. Oreadea | ASTERACEAE |  | 1 |  | 1 |  |  |  |  |  | 1 | 1 | 1 |  |  |
| Hieracium sect. Prenanthoidea | ASTERACEAE |  | 1 |  |  |  |  |  |  |  | 1 | 1 |  |  |  |
| Hieracium sect. Subalpina | ASTERACEAE |  | 1 |  |  |  |  | 1 |  |  | 1 | 1 |  |  |  |
| Hieracium sect. Tridentata | ASTERACEAE | 1? |  |  | 1 |  | 1 | 1 |  | 1 | 1 | 1 | 1 |  |  |
| Hieracium sect. Vulgata | ASTERACEAE |  |  |  | 1 |  |  |  |  |  | 1 | 1 | 1 |  |  |
| Hierochloë alpina (Sw. ex Willd.) Roem. & Schult. | POACEAE | 1 |  | 1 |  |  | 1 | 1 | 1 | 1 | 1 |  |  |  |  |
| Hierochloë odorata (L.) P.Beauv. subsp. odorata | POACEAE | 1 | 1 |  |  |  | 0 | 0 |  | 1 | 1 | 1 | 1 |  | Vicariant taxon in E Canada & W Greenland: subsp. arctica (J.Presl) Tzvelev (also in Fennoscandia etc.) |
| Hierochloë orthantha T.J.Sørensen | POACEAE | 1 |  |  |  |  | 1 | 1 |  |  |  |  |  |  |  |
| Hippuris lanceolata Retz. | PLANTAGINACEAE |  | 1 | 1 |  |  | 1 | 1 | 1 | 1 | 1 |  |  |  |  |
| Hippuris tetraphylla L. f. | PLANTAGINACEAE |  | 1 |  |  |  | 1 |  |  | 1? | 1 |  |  |  |  |
| Hippuris vulgaris L. | PLANTAGINACEAE | 1 | 1 |  |  |  | 1 |  |  | 1 | 1 | 1 | 1 |  |  |
| Holcus mollis L. | POACEAE |  |  |  | 1 |  |  |  |  |  | 1 | 1 | 1 |  |  |
| Honckenya peploides (L.) Ehrh. subsp. diffusa (Hornem.) Hultén | CARYOPHYLLACEAE |  | 1 | 1 | 1? |  | 1 | 1 | 1 | 1 | 1 |  |  |  |  |
| Honckenya peploides (L.) Ehrh. subsp. peploides | CARYOPHYLLACEAE |  |  |  | 1? |  |  |  |  |  | 1 | 1 |  |  |  |
| Huperzia appressa (Bach.Pyl. ex Desv.) Á.Löve & D.Löve | LYCOPODIACEAE | 1 | 1 |  | 1 | 1 | 1 | 1 |  | 1 | 1 | 1 | 1? |  |  |
| Huperzia arctica (Grossh. ex Tolm.) Sipliv. | LYCOPODIACEAE |  | 1 | 1 |  |  | 1 | 1 | 1 | 1 | 1 |  |  |  |  |
| Huperzia selago (L.) Bernh. ex Schrank & Mart. | LYCOPODIACEAE | 1 | 1? |  |  |  | 1 | 1 |  | 1 | 1 | 1 | 1 |  |  |
| Hydrocotyle vulgaris L. | ARALIACEAE | 1 | 1 |  |  |  |  |  |  |  | 1 | 1 | 1 |  |  |
| Hymenophyllum wilsonii Hook. | HYMENOPHYLLACEAE |  | 1 |  | 1 |  |  |  |  |  | 1 | 1 |  |  |  |
| Hypericum maculatum Crantz | HYPERICACEAE |  |  |  | 1 |  |  |  |  |  | 1 | 1 | 1 |  |  |
| Hypericum pulchrum L. | HYPERICACEAE |  |  |  | 1 |  |  |  |  |  | 1 | 1 | 1 |  |  |
| Isoëtes echinospora Durieu | ISOËTACEAE |  | 1 |  | 1 |  |  |  |  |  | 1 | 1 | 1 |  |  |
| Isoëtes lacustris L. | ISOËTACEAE |  | 1 |  | 1 |  |  | 1 |  |  | 1 | 1 | 1 |  |  |
| Isoëtes muricata Durieu | ISOËTACEAE | 1 |  |  |  |  | 1 | 1 |  |  |  |  |  |  |  |
| Juncus albescens (Lange) Fernald | JUNCACEAE | 1 |  | 1 |  |  | 1 | 1 | 1 |  |  |  |  |  |  |
| Juncus alpinoarticulatus Chaix | JUNCACEAE | 1 | 1 |  |  |  | 1 | 1 |  | 1 | 1 | 1 | 1 |  |  |
| Juncus arcticus Willd. subsp. arcticus | JUNCACEAE | 1 | 1 | 1 |  |  | 1 | 1 |  | 1 | 1 |  | 1 |  |  |
| Juncus arcticus Willd. subsp. intermedius Hyl. | JUNCACEAE |  | 1 |  |  |  |  |  |  |  |  |  |  | End | Intermediate between subsp. arcticus and subsp. balticus |
| Juncus articulatus L. | JUNCACEAE |  | 1 |  | 1 |  | 1 |  |  |  | 1 | 1 | 1 |  |  |
| Juncus balticus Willd. subsp. balticus | JUNCACEAE |  | 1 |  | 1 |  |  | 1 |  |  | 1 | 1 |  |  |  |
| Juncus biglumis L. | JUNCACEAE |  | 1 | 1 | 1 |  | 1 | 1 | 1 | 1 | 1 | 1 |  |  |  |
| Juncus bulbosus L. | JUNCACEAE |  | 1 |  | 1 |  |  |  |  |  | 1 | 1 | 1 |  |  |
| Juncus castaneus Sm. | JUNCACEAE | 1 | 1 |  |  |  |  |  |  | 1 | 1 | 1 | 1 |  |  |
| Juncus conglomeratus L. | JUNCACEAE |  |  |  | 1 |  |  |  |  |  | 1 | 1 | 1 |  |  |
| Juncus effusus L. | JUNCACEAE |  |  |  | 1 |  |  |  |  |  | 1 | 1 | 1 |  |  |
| Juncus filiformis L. | JUNCACEAE | 1 | 1 |  |  | 1 | 1 | 1 |  | 1 | 1 | 1 | 1 |  |  |
| Juncus gerardii Loisel. subsp. gerardii | JUNCACEAE |  | 1 |  |  |  | 1 | 1 |  | 0 | 1 | 1 |  |  | Vicariant taxon in N Urals: subsp. atrofuscus (Rupr.) Printz (also W to N Norway) |
| Juncus leucochlamys V.J.Zinger ex V.I.Krecz. | JUNCACEAE |  | 1 | 1 |  |  | 1 | 1 | 1 |  |  |  |  |  |  |
| Juncus ranarius Songeon & E.P.Perrier | JUNCACEAE | 1 | 1 |  |  | 1 | 1 | 1 |  | 1 | 1 | 1 | 1 |  |  |
| Juncus squarrosus L. | JUNCACEAE |  | 1 |  | 1 |  |  | 1 |  |  | 1 | 1 | 1 |  |  |
| Juncus subtilis E.Mey. | JUNCACEAE | 1 |  |  |  |  | 1 | 1 |  |  |  |  |  |  |  |
| Juncus trifidus L. | JUNCACEAE | 1 | 1 |  | 1 |  | 1 | 1 |  | 1 | 1 | 1 | 1 |  |  |
| Juncus triglumis L. | JUNCACEAE | 1 | 1 |  | 1 |  | 1 |  |  | 1 | 1 | 1 | 1 |  |  |
| Juniperus communis L. subsp. communis | CUPRESSACEAE | 1 | 1 |  |  |  | 0 |  |  | 1 | 1 | 1 | 1 |  | Vicariant taxon in E Canada: subsp. depressa (Pursh) Franco (across North America) |
| Juniperus communis L. subsp. nana Syme | CUPRESSACEAE |  | 1 |  | 1 |  |  | 1 |  | 1 | 1 | 1 |  |  |  |
| Kalmia procumbens (L.) Gift & Kron ex Galasso, Banfi & F.Conti (Loiseleuria procumbens (L.) Loisel.) | ERICACEAE | 1 | 1 |  | 1 | 1 | 1 | 1 |  | 1 | 1 | 1 | 1 |  |  |
| Kobresia myosuroides (Vill.) Fiori | CYPERACEAE | 1 | 1 |  |  |  | 1 | 1 | 1 | 1? | 1 |  | 1 |  |  |
| Kobresia simpliciuscula (Wahlenb.) Mack. subsp. subholarctica T.V.Egorova | CYPERACEAE | 1 |  | 1 |  |  | 1 | 1 | 1 |  | 0 | 0 | 0 |  | Vicariant taxon in S Scandinavia, Britain & C Europe: subsp. simpliciuscula |
| Koenigia islandica L. | POLYGONACEAE |  | 1 | 1 | 1 |  | 1 | 1 | 1 | 1 | 1 | 1 |  |  |  |
| Lathyrus japonicus Willd. | FABACEAE |  | 1 |  |  |  | 1 | 1 |  |  | 1 | 1 |  |  |  |
| Lathyrus palustris L. subsp. pilosus (Cham.) Hultén | FABACEAE |  | 1 |  |  |  | 1 |  |  | 1 | 1 | 0 | 0 |  | Vicariant taxon in S Scandinavia, Britain & C Europe: subsp. palustris |
| Leymus arenarius (L.) Hochst. | POACEAE |  | 1 |  | 1 |  |  | 1 |  | 1 | 1 | 1 |  |  |  |
| Leymus mollis (Trin.) Pilg. subsp. mollis | POACEAE | 1 |  |  |  |  | 1 | 1 |  |  |  |  |  |  |  |
| Ligusticum scothicum L. subsp. scothicum | APIACEAE |  | 1 |  | 1 |  | 1 | 1 |  |  | 1 | 1 |  |  |  |
| Limnorchis hyperborea (L.) Rydb. (Platanthera hyperborea (L.) Lindl.) | ORCHIDACEAE |  | 1 |  |  |  |  | 1 |  |  |  |  |  |  | Endemic to Greenland (W & E) and Iceland; related to North American L. aquilonis (Sheviak) Rebrist. & Elven and L. huronensis (Nutt.) Rydb. |
| Limosella aquatica L. | SCROPHULARIACEAE | 1 | 1 |  |  |  | 1 | 1 |  |  | 1 | 1 | 1 |  |  |
| Linnaea borealis L. subsp. americana (J.Forbes) Hultén | LINNAEACEAE | 1 |  |  |  |  | 1 | 1 |  |  | 0 | 0 | 0 |  | Vicariant taxon in Europe (and Asia): subsp. borealis |
| Linum catharticum L. | LINACEAE |  | 1 |  | 1 |  |  |  |  |  | 1 | 1 | 1 |  |  |
| Listera cordata (L.) R.Br. | ORCHIDACEAE | 1 | 1 |  | 1 |  | 1 | 1 |  | 1 | 1 | 1 | 1 |  |  |
| Listera ovata (L.) R.Br. | ORCHIDACEAE | 1 | 1 |  |  |  |  |  |  |  | 1 | 1 | 1 |  |  |
| Littorella uniflora (L.) Asch. | PLANTAGINACEAE |  | 1 |  | 1 |  |  |  |  |  | 1 | 1 | 1 |  |  |
| Lobelia dortmanna L. | CAMPANULACEAE |  |  |  | 1 |  | 1 |  |  |  | 1 | 1 |  |  |  |
| Lomatogonium rotatum (L.) Fr. ex Fernald | GENTIANACEAE |  | 1 |  |  |  | 1 | 1 |  |  | 1 |  |  |  |  |
| Luzula arcuata (Wahlenb.) Sw. | JUNCACEAE |  | 1 | 1 | 1 |  |  | 1 |  | 1 | 1 | 1 |  |  |  |
| Luzula campestris (L.) DC. | JUNCACEAE |  |  |  | 1 |  |  |  |  |  | 1 | 1 | 1 |  |  |
| Luzula confusa Lindeb. | JUNCACEAE | 1 |  | 1 |  |  | 1 | 1 | 1 | 1 | 1 |  |  |  |  |
| Luzula congesta (Thuill.) Lej. | JUNCACEAE |  |  |  | 1 |  |  |  |  |  | 1 | 1 | 1 |  |  |
| Luzula multiflora (Ehrh.) Lej. subsp. frigida (Buchenau) V.I.Krecz. | JUNCACEAE | 1 | 1 |  |  | 1 | 1 | 1 |  | 1 | 1 |  | 1? |  |  |
| Luzula multiflora (Ehrh.) Lej. subsp. multiflora | JUNCACEAE |  | 1 |  | 1 |  |  |  |  |  | 1 | 1 | 1 |  |  |
| Luzula nivalis (Laest.) Spreng. | JUNCACEAE | 1 |  | 1 |  |  | 1 | 1 | 1 | 1 | 1 |  |  |  |  |
| Luzula parviflora (Ehrh.) Desv. subsp. parviflora | JUNCACEAE | 1 |  |  |  |  | 1 | 1 |  | 1 | 1 |  |  |  |  |
| Luzula spicata (L.) DC. | JUNCACEAE | 1 | 1 |  | 1 | 1 | 1 | 1 |  | 1 | 1 | 1 | 1 |  |  |
| Luzula sudetica (Willd.) Schult. | JUNCACEAE |  | 1 |  |  |  |  |  |  |  | 1 |  | 1 |  |  |
| Luzula sylvatica (Huds.) Gaudin | JUNCACEAE |  |  |  | 1 |  |  |  |  |  | 1 | 1 | 1 |  |  |
| Luzula wahlenbergii Rupr. | JUNCACEAE | 1 |  | 1 |  |  | 1 |  |  | 1 | 1 |  |  |  |  |
| Lychnis flos-cuculi L. | CARYOPHYLLACEAE |  | 1 |  | 1 |  |  |  |  |  | 1 | 1 | 1 |  |  |
| Lycopodium annotinum L. subsp. alpestre (Hartm.) Á.Löve & D.Löve | LYCOPODIACEAE | 1 | 1 |  | 1 | 1 | 1 | 1 |  | 1 | 1 | 1? | 1? |  |  |
| Lycopodium annotinum L. subsp. annotinum | LYCOPODIACEAE | 1 | 1 |  |  | 1 | 1 | 1 |  | 1 | 1 | 1 | 1 |  |  |
| Lycopodium clavatum L. subsp. clavatum | LYCOPODIACEAE | 1 | 1 |  |  |  | 1 |  |  | 1 | 1 | 1 | 1 |  |  |
| Lycopodium clavatum L. subsp. monostachyon (Grev. & Hook.) Selander | LYCOPODIACEAE | 1 |  |  |  |  | 1 | 1 |  | 1 | 1 |  |  |  |  |
| Lysimachia europaea (L.) U.Manns & Anderb. (Trientalis europaea L.) | MYRSINACEAE | 1 | 1 |  |  |  |  |  |  | 1 | 1 | 1 | 1 |  |  |
| Lysimachia maritima (L.) Galasso, Banfi & Soldano (Glaux maritima L.) | MYRSINACEAE |  | 1 |  |  |  | 1 |  |  |  | 1 | 1 |  |  |  |
| Lysimachia nemorum L. | MYRSINACEAE |  |  |  | 1 |  |  |  |  |  | 1 | 1 | 1 |  |  |
| Lysimachia tenella L. (Anagallis tenella (L.) L.) | MYRSINACEAE |  |  |  | 1 |  |  |  |  |  |  | 1 | 1 |  |  |
| Melampyrum sylvaticum L. | OROBANCHACEAE | 1 | 1 |  |  |  |  |  |  |  | 1 | 1 | 1 |  |  |
| Mentha aquatica L. | LAMIACEAE |  | 1 |  | 1 |  |  |  |  |  | 1 | 1 | 1 |  |  |
| Menyanthes trifoliata L. subsp. minor (Raf.) Á.Löve & Ritchie | MENYANTHACEAE | 1 |  |  |  |  | 1 | 1 |  |  |  |  |  |  |  |
| Menyanthes trifoliata L. subsp. trifoliata | MENYANTHACEAE | 1 | 1 |  | 1 |  |  |  |  | 1 | 1 | 1 | 1 |  |  |
| Mertensia maritima (L.) Gray subsp. maritima | BORAGINACEAE |  | 1 |  | 1 |  |  |  |  |  | 1 | 1 |  |  |  |
| Mertensia maritima (L.) Gray subsp. tenella (Th.Fr.) Elven & Skarpaas | BORAGINACEAE | 1 |  | 1 |  |  | 1 | 1 | 1 |  |  |  |  |  |  |
| Micranthes foliolosa (R.Br.) Gornall | SAXIFRAGACEAE |  | 1 | 1 |  |  | 1 | 1 | 1 | 1 | 1 |  |  |  |  |
| Micranthes hieraciifolia (Waldst. & Kit. ex Willd.) Haw. | SAXIFRAGACEAE | 1 |  | 1 |  |  | 1 |  |  | 1 | 1 |  | 1 |  |  |
| Micranthes nivalis (L.) Small | SAXIFRAGACEAE |  | 1 | 1 | 1 |  | 1 | 1 | 1 | 1 | 1 | 1 |  |  |  |
| Micranthes stellaris (L.) Galasso, Banfi & Soldano | SAXIFRAGACEAE |  | 1 |  | 1 |  | 1 | 1 |  |  | 1 | 1 | 0 |  | Vicariant taxon in C Europe: M. engleri (Dalla Torre) Galasso, Banfi & Soldano |
| Micranthes tenuis (Wahlenb.) Small | SAXIFRAGACEAE | 1 | 1 | 1 |  | 1 | 1 | 1 | 1 | 1 | 1 |  |  |  |  |
| Milium effusum L. var. effusum | POACEAE | 1 | 1 |  |  |  | 0 |  |  | 1 | 1 | 1 | 1 |  | Vicariant taxon in E Canada: var. cisatlanticum Fernald |
| Minuartia biflora (L.) Schinz & Thell. | CARYOPHYLLACEAE | 1 | 1 | 1 |  |  | 1 | 1 | 1 | 1 | 1 |  | 1 |  |  |
| Minuartia groenlandica (Retz.) Ostenf. | CARYOPHYLLACEAE | 1 |  |  |  | 1 | 1 | 1 |  |  |  |  |  |  |  |
| Minuartia rossii (R.Br. ex Richardson) Graebn. | CARYOPHYLLACEAE | 1 |  | 1 |  |  | 1 | 1 | 1 |  |  |  |  |  |  |
| Minuartia rubella (Wahlenb.) Hiern | CARYOPHYLLACEAE |  | 1 | 1 | 1 |  | 1 | 1 | 1 | 1 | 1 | 1 |  |  |  |
| Minuartia stricta (Sw.) Hiern | CARYOPHYLLACEAE | 1 | 1 | 1 |  | 1 | 1 | 1 |  | 1 | 1 | 1 | 1 |  |  |
| Molinia caerulea (L.) Moench | POACEAE |  |  |  | 1 |  |  |  |  |  | 1 | 1 | 1 |  |  |
| Montia fontana L. | MONTIACEAE |  | 1 |  | 1 |  | 1 | 1 |  |  | 1 | 1 |  |  |  |
| Myosotis scorpioides L. | BORAGINACEAE | 1 | 1 |  | 1 |  |  |  |  | 1 | 1 | 1 | 1 |  |  |
| Myosotis secunda Al.Murray | BORAGINACEAE |  |  |  | 1 |  |  |  |  |  | 1? | 1 |  |  |  |
| Myosotis stricta Link ex Roem. & Schult. | BORAGINACEAE |  | 1 |  |  |  |  |  |  |  | 1 |  | 1 |  |  |
| Myriophyllum alterniflorum DC. | HALORAGACEAE | 1 | 1 |  | 1 |  | 1 | 1 |  |  | 1 | 1 | 1 |  |  |
| Myriophyllum sibiricum Kom. | HALORAGACEAE |  | 1 |  |  |  | 1 | 1 |  | 1 | 1 |  |  |  |  |
| Nardus stricta L. | POACEAE | 1 | 1 |  | 1 |  | 1 | 1 |  |  | 1 | 1 | 1 |  |  |
| Narthecium ossifragum (L.) Huds. | NARTHECIACEAE |  |  |  | 1 |  |  |  |  |  | 1 | 1 |  |  |  |
| Omalotheca norvegica (Gunnerus) Sch.Bip. & F.W.Schultz | ASTERACEAE | 1 | 1 |  |  |  | 1 | 1 |  | 1 | 1 | 1 | 1 |  |  |
| Omalotheca supina (L.) DC. | ASTERACEAE | 1 | 1 |  | 1 | 1 | 1 | 1 |  | 1 | 1 | 1 | 1 |  |  |
| Omalotheca sylvatica (L.) Sch.Bip. & F.W.Schultz | ASTERACEAE | 1 | 1 |  |  |  | 1 |  |  |  | 1 | 1 | 1 |  |  |
| Ophioglossum azoricum C.Presl | OPHIOGLOSSACEAE |  | 1 |  |  |  |  |  |  |  |  | 1 |  |  |  |
| Orchis mascula (L.) L. | ORCHIDACEAE |  |  |  | 1 |  |  |  |  |  | 1 | 1 | 1 |  |  |
| Orthilia secunda (L.) House | ERICACEAE | 1 | 1 |  |  |  | 1 | 0 |  | 1 | 1 | 1 | 1 |  | Vicariant taxon in W Greenland (across North America and Asia): O. obtusata (Turcz.) H.Hara |
| Oxalis acetosella L. | OXALIDACEAE | 1 | 1 |  |  |  |  |  |  |  | 1 | 1 | 1 |  |  |
| Oxycoccus microcarpus Turcz. ex Rupr. | ERICACEAE | 1 | 1 |  |  |  | 1 | 0 |  | 1 | 1 | 1 | 1 |  | Vicariant taxon in W Greenland and parts of E Canada: O. palustris Pers. subsp. microphyllus (Lange) Á.Löve & D.Löve |
| Oxyria digyna (L.) Hill | POLYGONACEAE | 1 | 1 | 1 | 1 | 1 | 1 | 1 | 1 | 1 | 1 | 1 | 1 |  |  |
| Papaver cornwallisense D.Löve | PAPAVERACEAE | 1 |  | 1 |  |  | 1 |  | 1 |  |  |  |  |  |  |
| Papaver dahlianum Nordh. | PAPAVERACEAE | 1 |  | 1 |  |  | 1 | 1 | 1 | 1 | 1 |  |  |  |  |
| Papaver labradoricum (Fedde) Solstad & Elven | PAPAVERACEAE | 1 |  |  |  |  | 1 | 1 |  |  |  |  |  |  |  |
| Papaver lapponicum (Tolm.) Nordh. subsp. lapponicum | PAPAVERACEAE | 1 |  |  |  |  |  |  |  | 1 | 1 |  |  |  |  |
| Papaver lapponicum (Tolm.) Nordh. subsp. occidentale (C.E.Lundstr.) Knaben | PAPAVERACEAE | 1 |  |  |  |  | 1 | 1 | 1 |  |  |  |  |  |  |
| Papaver radicatum Rottb. | PAPAVERACEAE |  | 1 |  | 1 |  |  |  |  |  | 1 |  |  |  |  |
| Paris quadrifolia L. | MELANTHIACEAE | 1 | 1 |  |  |  |  |  |  | 1 | 1 | 1 | 1 |  |  |
| Parnassia palustris L. subsp. palustris | PARNASSIACEAE | 1 | 1 |  |  |  | 0 |  |  | 1 | 1 | 1 | 1 |  | Vicariant taxon in E Canada (across North America): subsp. neogaea (Fernald) Hultén |
| Pedicularis dasyantha (Trautv.) Hadač | OROBANCHACEAE |  |  | 1 |  |  | 0 | 0 | 0 | 1 |  |  |  |  | Vicariant taxon in Canada and N and W Greenland: Pedicularis lanata Willd. ex Cham. & Schltdl. |
| Pedicularis flammea L. | OROBANCHACEAE |  | 1 |  |  |  | 1 | 1 | 1 |  | 1 |  |  |  |  |
| Pedicularis hirsuta L. | OROBANCHACEAE | 1 |  | 1 |  |  | 1 | 1 | 1 | 1 | 1 |  |  |  |  |
| Pedicularis lapponica L. | OROBANCHACEAE | 1 |  |  |  |  | 1 | 1 |  | 1 | 1 |  |  |  |  |
| Pedicularis palustris L. | OROBANCHACEAE |  |  |  | 1 |  |  |  |  |  | 1 | 1 | 1 |  |  |
| Persicaria amphibia (L.) Delarbre subsp. amphibia | POLYGONACEAE |  | 1 |  | 1 |  | 0 |  |  |  | 1 | 1 | 1 |  | Vicariant taxon i E Canada (across North America): subsp. laevimarginata (Hultén) Soják |
| Petasites frigidus (L.) Fr. | ASTERACEAE |  |  | 1 |  |  |  |  |  | 1 | 1 |  |  |  |  |
| Phegopteris connectilis (Michx.) Watt | THELYPTERIDACEAE | 1 | 1 |  | 1 |  | 1 | 1 |  | 1 | 1 | 1 | 1 |  |  |
| Phippsia algida (Sol.) R.Br. | POACEAE |  | 1 | 1 |  |  | 1 | 1 | 1 | 1 | 1 |  |  |  |  |
| Phippsia concinna (Th.Fr.) Lindeb. | POACEAE | 1 |  | 1 |  |  | 1 | 1 | 1 | 1 | 1 |  |  |  |  |
| Phleum alpinum L. | POACEAE | 1 | 1 |  | 1 |  | 1 | 1 |  | 1 | 1 | 1 | 1 |  |  |
| Phragmites australis (Cav.) Trin. ex Steud. | POACEAE |  |  |  | 1 |  | 1 |  |  | 1 | 1 | 1 | 1 |  |  |
| Phyllodoce caerulea (L.) Bab. | ERICACEAE |  | 1 |  |  |  | 1 | 1 |  | 1 | 1 | 1 |  |  |  |
| Physaria arctica (Wormsk. ex Hornem.) O'Kane & Al-Shehbaz (Lesquerella arctica (Wormsk. ex Hornem.) S.Watson) | BRASSICACEAE | 1 |  |  |  |  | 1 | 1 | 1 |  |  |  |  |  |  |
| Pilosella islandica (Lange) Á.Löve | ASTERACEAE |  | 1 |  |  |  |  |  |  |  |  | 0 | 0 | End | Closely related to or possibly identical with the European P. floribunda (Wimm. & Grab.) Fr. |
| Pinguicula alpina L. | LENTIBULARIACEAE |  |  | 1 |  |  |  |  |  | 1 | 1 |  | 1 |  |  |
| Pinguicula vulgaris L. | LENTIBULARIACEAE | 1 | 1 |  | 1 |  | 1 | 1 |  | 1 | 1 | 1 | 1 |  |  |
| Plantago coronopus L. | PLANTAGINACEAE |  |  |  | 1 |  |  |  |  |  | 1 | 1 |  |  |  |
| Plantago lanceolata L. | PLANTAGINACEAE |  | 1 |  | 1 |  |  |  |  |  | 1 | 1 | 1 |  |  |
| Plantago maritima L. | PLANTAGINACEAE | 1 | 1 |  | 1 |  | 1 | 1 |  | 1 | 1 | 1 | 1 |  |  |
| Pleuropogon sabinei R.Br. | POACEAE | 1 |  | 1 |  |  | 1 |  | 1 | 1 |  |  |  |  |  |
| Poa abbreviata R.Br. | POACEAE | 1 |  | 1 |  |  | 1 | 1 | 1 | 1 |  |  |  |  |  |
| Poa alpina L. var. alpina | POACEAE | 1 | 1 | 1 | 1 | 1 | 1 | 1 |  | 1 | 1 | 1 | 1 |  |  |
| Poa alpina L. var. vivipara L. | POACEAE |  | 1 | 1 |  |  |  |  |  | 1? | 1 | 1 |  |  |  |
| Poa arctica R.Br. subsp. arctica | POACEAE | 1 |  | 1 |  |  | 1 | 1 | 1 | 1 | 1 |  |  |  |  |
| Poa arctica R.Br. subsp. caespitans Simmons ex Nannf. | POACEAE | 1 |  | 1 |  |  | 1 | 1 | 1 | 1 | 1 |  |  |  |  |
| Poa flexuosa Sm. | POACEAE |  | 1 |  |  |  | 0 | 0 |  |  | 1 | 1 | 0 |  | Vicariant taxa in C Europe: P. laxa Haenke; in E Canada & W Greenland: undescribed species |
| Poa glauca Vahl | POACEAE | 1 | 1 | 1 | 1 | 1 | 1 | 1 | 1 | 1 | 1 | 1 | 1 |  |  |
| Poa hartzii Gand. | POACEAE | 1 |  | 1 |  |  | 1 | 1 | 1 |  |  |  |  |  |  |
| Poa jemtlandica (Almq.) K.Richt. | POACEAE |  | 1 |  |  |  |  |  |  |  | 1 | 1 |  |  |  |
| Poa nemoralis L. | POACEAE | 1 | 1 |  | 1 |  |  | 1 |  | 1 | 1 | 1 | 1 |  |  |
| Poa pratensis L. subsp. alpigena (Lindm.) Hiitonen | POACEAE |  | 1 | 1 | 1 |  | 1 | 1 | 1 | 1 | 1 |  |  |  |  |
| Poa pratensis L. subsp. colpodea (Th.Fr.) Tzvelev | POACEAE | 1 |  | 1 |  |  | 1 | 1 | 1 | 1 |  |  |  |  |  |
| Poa pratensis L. subsp. irrigata (Lindm.) H.Lindb. | POACEAE | 1 | 1 |  | 1 | 1 |  | 1? |  |  | 1 | 1 | 1? |  |  |
| Polemonium boreale Adams | POLEMONIACEAE | 1 |  | 1 |  |  |  |  |  | 1 | 1 |  |  |  |  |
| Polygala serpyllifolia Hosé | POLYGALACEAE |  |  |  | 1 |  |  |  |  |  | 1 | 1 | 1 |  |  |
| Polygala vulgaris L. | POLYGALACEAE |  |  |  | 1 |  |  |  |  |  | 1 | 1 | 1 |  |  |
| Polygonum boreale Lange | POLYGONACEAE |  | 1 |  | 1? |  | 1 | 1? |  |  | 1 | 1 |  |  |  |
| Polypodium vulgare L. | POLYPODIACEAE | 1 | 1 |  | 1 |  |  |  |  | 1 | 1 | 1 | 1 |  |  |
| Polystichum lonchitis (L.) Roth | DRYOPTERIDACEAE | 1 | 1 |  | 1 |  | 1 | 1 |  | 1 | 1 | 1 | 1 |  |  |
| Populus tremula L. | SALICACEAE | 1 | 1 |  |  |  |  |  |  | 1 | 1 | 1 | 1 |  |  |
| Potamogeton alpinus Balb. subsp. alpinus | POTAMOGETONACEAE | 1 | 1 |  | 1 |  | 0 | 0 |  | 1 | 1 | 1 | 1 |  | Vicariant taxon in E Canana & W Greenland: subsp. tenuifolius (Raf.) Hultén |
| Potamogeton berchtoldii Fieber | POTAMOGETONACEAE | 1 | 1 |  | 1 |  | 1 |  |  | 1 | 1 | 1 | 1 |  |  |
| Potamogeton gramineus L. | POTAMOGETONACEAE | 1 | 1 |  | 1 |  | 1 | 1 |  | 1 | 1 | 1 | 1 |  |  |
| Potamogeton groenlandicus Hagstr. | POTAMOGETONACEAE | 1? |  |  |  |  |  | 1 |  |  |  |  |  |  | Endemic to Greenland (W & E?), related to American and Asian P. subsibiricus Hagstr. |
| Potamogeton natans L. | POTAMOGETONACEAE | 1 | 1 |  | 1 |  | 1 | 1? |  | 1 | 1 | 1 | 1 |  |  |
| Potamogeton perfoliatus L. subsp. perfoliatus | POTAMOGETONACEAE | 1 | 1 |  | 1 |  | 0 |  |  | 1 | 1 | 1 | 1 |  | Vicariant taxon in E Canada (across North America): subsp. richardsonii (A.Benn.) Hultén |
| Potamogeton polygonifolius Pourr. | POTAMOGETONACEAE |  |  |  | 1 |  | 1 |  |  |  | 1 | 1 |  |  |  |
| Potamogeton praelongus Wulfen | POTAMOGETONACEAE | 1 | 1 |  | 1 |  | 1 | 1 |  | 1 | 1 | 1 | 1 |  |  |
| Potentilla anserina L. subsp. anserina | ROSACEAE |  | 1 |  | 1 |  | 1? |  |  |  | 1 | 1 | 1 |  |  |
| Potentilla anserina L. subsp. groenlandica Tratt. | ROSACEAE |  | 1 |  |  |  | 1 | 1 |  |  | 1 |  |  |  |  |
| Potentilla arenosa (Turcz.) Juz. subsp. arenosa | ROSACEAE | 1 |  |  |  |  | 1 | 1 | 1 | 1 |  |  |  |  |  |
| Potentilla arenosa (Turcz.) Juz. subsp. chamissonis (Hultén) Elven & D.F.Murray | ROSACEAE | 1 |  | 1 |  |  | 1 | 1 |  |  | 1 |  |  |  |  |
| Potentilla crantzii (Crantz) Beck ex Fritsch | ROSACEAE | 1 | 1 | 1 | 1 | 1 | 1 | 1 |  | 1 | 1 | 1 | 1 |  |  |
| Potentilla erecta (L.) Raeusch. | ROSACEAE |  | 1 |  | 1 |  |  |  |  |  | 1 | 1 | 1 |  |  |
| Potentilla glaucophylla Lehm. | ROSACEAE | 1 |  |  |  |  | 1 | 1 |  |  |  |  |  |  |  |
| Potentilla hyparctica Malte subsp. elatior (Abrom.) Elven & D.F.Murray | ROSACEAE | 1 |  |  |  |  | 1 | 1 |  |  |  |  |  |  |  |
| Potentilla hyparctica Malte subsp. hyparctica | ROSACEAE | 1 |  | 1 |  |  | 1 | 1 | 1 | 1 |  |  |  |  |  |
| Potentilla insularis Soják | ROSACEAE |  |  | 1 |  |  |  |  |  |  |  |  |  | End | Endemic to Svalbard |
| Potentilla lyngei Soják | ROSACEAE | 1 |  | 1 |  |  |  |  |  | 1 | 1 |  |  |  |  |
| Potentilla nivea L. | ROSACEAE | 1 |  | 1 |  | 1 | 1 | 1 | 1 | 1 | 1 |  | 1 |  |  |
| Potentilla pedersenii Rydb. | ROSACEAE | 1 |  |  |  |  | 1 | 1 | 1 |  |  |  |  |  |  |
| Potentilla pulchella R.Br. | ROSACEAE | 1 |  | 1 |  |  | 1 | 1 | 1 | 1 |  |  |  |  |  |
| Potentilla rubella T.J.Sørensen | ROSACEAE |  |  |  |  | 1 |  |  |  |  |  |  |  |  | Not endemic, but hybridogenous and with polytopic origins in at least three parts of Siberia, Russian Far East, and Greenland |
| Potentilla stipularis L. | ROSACEAE | 1 |  |  |  |  |  |  |  | 1 |  |  |  |  |  |
| Primula egaliksensis Wormsk. ex Hornem. | PRIMULACEAE |  | 1 |  |  |  | 1 | 1 |  |  |  |  |  |  |  |
| Primula stricta Hornem. | PRIMULACEAE |  | 1 |  |  |  | 1 | 1 |  | 1 | 1 |  |  |  |  |
| Primula vulgaris Huds. | PRIMULACEAE |  |  |  | 1 |  |  |  |  |  | 1 | 1 | 1 |  |  |
| Prunella vulgaris L. subsp. vulgaris | LAMIACEAE | 1 | 1 |  | 1 |  | 0 |  |  | 1 | 1 | 1 | 1 |  | Vicariant taxon in E Canada (across North America): subsp. lanceolata (W.P.C.Barton) Hultén |
| Pseudorchis straminea (Fernald) Soó | ORCHIDACEAE |  | 1 |  | 1 |  | 1 | 1 |  | 1? | 1 |  |  |  |  |
| Puccinellia andersonii Swallen | POACEAE | 1 |  |  |  |  | 1 | 1 | 1 |  |  |  |  |  |  |
| Puccinellia angustata (R.Br.) E.L.Rand & Redfield | POACEAE | 1 |  | 1 |  |  | 1 | 1 | 1 |  |  |  |  |  |  |
| Puccinellia bruggemannii T.J.Sørensen | POACEAE | 1 |  |  |  |  | 1 |  | 1 |  |  |  |  |  |  |
| Puccinellia capillaris (Lilj.) Jansen | POACEAE |  |  |  | 1 |  |  |  |  |  | 1 | 1 |  |  |  |
| Puccinellia coarctata Fernald & Weath. | POACEAE |  | 1 | 1 |  |  | 1 | 1 |  |  | 1 |  |  |  |  |
| Puccinellia maritima (Huds.) Parl. | POACEAE |  | 1 |  | 1 |  | 1 | 1 |  |  | 1 | 1 |  |  |  |
| Puccinellia phryganodes (Trin.) Scribn. & Merr. subsp. neoarctica (Á.Löve & D.Löve) Elven | POACEAE | 1 |  |  |  |  | 1 | 1 | 1 | 0 | 0 |  |  |  | Vicariant taxon in N Fennoscandia & N Urals: subsp. sibirica (Hadač & Á.Löve) Elven |
| Puccinellia phryganodes (Trin.) Scribn. & Merr. subsp. vilfoidea (Á.Löve & D.Löve) Elven | POACEAE |  |  | 1 |  |  |  |  |  | 1 | 0 |  |  |  | Vicariant taxon in N Fennoscandia & N Urals: subsp. sibirica (Hadač & Á.Löve) Elven (subsp. vilfoidea in Novaya Zemlya) |
| Puccinellia svalbardensis Rønning | POACEAE |  |  | 1 |  |  |  |  |  |  |  |  |  | End | Endemic to Svalbard |
| Puccinellia vaginata (Lange) Fernald & Weath. | POACEAE | 1 |  |  |  |  | 1 | 1 | 1 |  |  |  |  |  |  |
| Puccinellia vahliana (Liebm.) Scribn. & Merr. | POACEAE | 1 |  | 1 |  |  | 1 | 1 | 1 | 1 |  |  |  |  |  |
| Pucciphippsia vacillans (Th.Fr.) Tzvelev | POACEAE | 1 |  | 1 |  |  | 1 |  | 1 | 1 |  |  |  |  |  |
| Pyrola grandiflora Radius | ERICACEAE |  | 1 |  |  |  | 1 | 1 | 1 | 1 | 0 |  |  |  | Vicariant taxon in Fennoscandia P. norvegica Knaben |
| Pyrola minor L. | ERICACEAE | 1 | 1 |  | 1 |  | 1 | 1 |  | 1 | 1 | 1 | 1 |  |  |
| Ranunculus arcticus Richardson | RANUNCULACEAE | 1 |  | 1 |  |  | 1 | 1 | 1 | 1 |  |  |  |  |  |
| Ranunculus auricomus L. agg. | RANUNCULACEAE | 1 | 1 | 1 | 1 | 1 |  |  |  | 1 | 1 | 1 | 1 |  | Two microspecies in E Greenland: R. boecheri (Fagerstr. & G.Kvist) Ericsson & R. hartzii (Fagerstr. & G. Kvist) Ericsson; one in Iceland: R. islandicus (Fagerstr. & G.Kvist) Ericsson; one in Svalbard: R. wilanderi (Nath.) Á.Löve & D.Löve; several in N Fennoscandia & N Urals |
| Ranunculus confervoides (Fr.) Fr. | RANUNCULACEAE | 1 | 1 |  |  | 1 | 1 | 1 |  | 1 | 1 |  | 1? |  |  |
| Ranunculus flammula L. | RANUNCULACEAE |  |  |  | 1 |  |  |  |  |  | 1 | 1 | 1 |  |  |
| Ranunculus glabratus (Lynge) Ericsson | RANUNCULACEAE | 1 |  |  |  |  |  |  |  | 1 |  |  |  |  | Perhaps better as part of the R. auricomus L. aggregate |
| Ranunculus glacialis L. | RANUNCULACEAE |  | 1 | 1 | 1 |  |  |  |  |  | 1 |  | 1 |  |  |
| Ranunculus hyperboreus Rottb. subsp. arnellii Scheutz | RANUNCULACEAE | 1 |  | 1 |  |  |  |  |  | 1 |  |  |  |  |  |
| Ranunculus hyperboreus Rottb. subsp. hyperboreus | RANUNCULACEAE |  | 1 | 1 |  |  | 1 | 1 | 1 | 1 | 1 |  |  |  |  |
| Ranunculus nivalis L. | RANUNCULACEAE | 1 |  | 1 |  |  | 1 | 1 | 1 | 1 | 1 |  |  |  |  |
| Ranunculus pygmaeus L. | RANUNCULACEAE | 1 | 1 | 1 |  |  | 1 | 1 | 1 | 1 | 1 |  | 1 |  |  |
| Ranunculus repens L. | RANUNCULACEAE |  | 1 |  | 1 |  |  |  |  |  | 1 | 1 | 1 |  |  |
| Ranunculus reptans L. | RANUNCULACEAE | 1 | 1 |  | 1 |  | 1 | 1 |  | 1 | 1 | 1 | 1 |  |  |
| Ranunculus subborealis Tzvelev subsp. pumilus (Wahlenb.) Elven | RANUNCULACEAE |  | 1 |  | 1 |  |  |  |  |  | 1 | 1 |  |  |  |
| Ranunculus subborealis Tzvelev subsp. villosus (Drabble) Elven | RANUNCULACEAE |  | 1 |  | 1 |  |  | 1 |  |  | 1 | 1 |  |  |  |
| Ranunculus subrigidus W.B.Drew | RANUNCULACEAE | 1 |  |  |  |  | 1 |  | 1 |  |  |  |  |  |  |
| Ranunculus sulphureus Sol. | RANUNCULACEAE | 1 |  | 1 |  |  | 1 | 1 | 1 | 1 | 1 |  |  |  |  |
| Ranunculus trichophyllus Chaix ex Vill. | RANUNCULACEAE | 1 |  |  |  | 1 | 1? | 1? |  | 1? | 1 | 1 | 1 |  |  |
| Rhinanthus minor L. subsp. groenlandicus (Ostenf.) Neuman | OROBANCHACEAE |  | 1 |  |  |  | 1 | 1 |  |  | 1 |  |  |  |  |
| Rhinanthus minor L. subsp. minor | OROBANCHACEAE |  | 1 |  | 1 |  |  |  |  |  | 1 | 1 | 1 |  |  |
| Rhodiola rosea L. | CRASSULACEAE | 1 | 1 | 1 | 1 | 1 | 1 | 1 |  | 1 | 1 | 1 | 1 |  |  |
| Rhododendron lapponicum (L.) Wahlenb. | ERICACEAE | 1 |  |  |  |  | 1 | 1 | 1 |  | 1 |  |  |  |  |
| Rorippa islandica (Oeder) Borbás | BRASSICACEAE | 1 | 1 |  |  |  |  | 1 |  |  | 1 | 1 | 1 |  |  |
| Rosa dumalis Bechst. | ROSACEAE | 1 | 1 |  |  |  |  |  |  |  | 1 | 1 | 1 |  |  |
| Rosa mollis Sm. | ROSACEAE |  |  |  | 1 |  |  |  |  |  | 1 | 1 | 1 |  |  |
| Rosa pimpinellifolia L. | ROSACEAE | 1 | 1 |  |  |  |  |  |  |  | 1 | 1 | 1 |  |  |
| Rubus chamaemorus L. | ROSACEAE |  |  | 1 |  |  | 1 | 1 |  | 1 | 1 | 1 |  |  |  |
| Rubus saxatilis L. | ROSACEAE | 1 | 1 |  | 1 |  |  | 1 |  | 1 | 1 | 1 | 1 |  |  |
| Rumex acetosa L. subsp. islandicus (Á.Löve) Ö.Nilsson | POLYGONACEAE |  | 1 |  | 1 |  |  |  |  |  |  |  |  | End | Very closely related to European races of R. acetosa L. |
| Rumex acetosella L. subsp. acetosella | POLYGONACEAE | 1 | 1 |  |  |  |  |  |  |  | 1 | 1 | 1 |  |  |
| Rumex acetosella L. subsp. arenicola Y.Mäkinen ex Elven | POLYGONACEAE |  | 1 |  |  |  | 1? | 1 |  | 1? | 1 |  |  |  |  |
| Rumex acetosella L. subsp. tenuifolius (Wallr.) O.Schwarz | POLYGONACEAE | 1 | 1 |  |  | 1 |  |  |  | 1? | 1 | 1 | 1 |  |  |
| Ruppia cirrhosa (Petagna) Grande | RUPPIACEAE |  | 1 |  |  |  |  |  |  |  | 1 | 1 |  |  |  |
| Ruppia maritima L. | RUPPIACEAE |  | 1 |  | 1 |  | 1 |  |  |  | 1 | 1 |  |  |  |
| Sagina caespitosa (J.Vahl) Lange | CARYOPHYLLACEAE |  | 1 | 1 |  |  | 1 | 1 | 1 |  | 1 |  |  |  |  |
| Sagina nivalis (Lindblom) Fr. | CARYOPHYLLACEAE |  | 1 | 1 | 1 |  | 1 | 1 | 1 | 1 | 1 | 1 |  |  |  |
| Sagina nodosa (L.) Fenzl subsp. borealis G.E.Crow | CARYOPHYLLACEAE |  | 1 |  | 1 |  | 1 | 1 |  |  | 1 | 0 |  |  | Vicariant taxon in S Scandinavia & Britain (and elsewhere in Europe): subsp. nodosa |
| Sagina procumbens L. | CARYOPHYLLACEAE | 1 | 1 |  | 1 |  | 1 | 1 |  |  | 1 | 1 | 1 |  |  |
| Sagina saginoides (L.) H.Karst. | CARYOPHYLLACEAE | 1 | 1 |  |  |  | 1 | 1 |  | 1 | 1 | 1 | 1 |  |  |
| Sagina subulata (Sw.) C.Presl | CARYOPHYLLACEAE |  | 1 |  | 1 |  |  |  |  |  | 1 | 1 | 1 |  |  |
| Salix arctica Pallas | SALICACEAE |  | 1 |  | 1 |  | 1 | 1 | 1 | 1 | 1 |  |  |  |  |
| Salix glauca L. subsp. callicarpaea (Trautv.) Böcher | SALICACEAE | 1 |  |  |  |  | 1 | 1 |  | 0 | 0 |  |  |  | Vicariant taxa in Fennoscandia subsp. glauca; and in Fennoscandia & N Urals: subsp. stipulifera (Flod. ex Häyrén) Hiitonen |
| Salix herbacea L. | SALICACEAE | 1 | 1 | 1 | 1 | 1 | 1 | 1 | 1 | 1 | 1 | 1 | 1 |  |  |
| Salix lanata L. | SALICACEAE |  | 1 | 1 | 1 |  |  |  |  | 1 | 1 | 1 |  |  |  |
| Salix phylicifolia L. | SALICACEAE | 1 | 1 |  | 1 |  |  |  |  | 1 | 1 | 1 | 1 |  |  |
| Salix polaris Wahlenb. | SALICACEAE |  |  | 1 |  |  |  |  |  | 1 | 1 |  |  |  |  |
| Salix reticulata L. | SALICACEAE |  |  | 1 |  |  | 1 |  |  | 1 | 1 | 1 | 1 |  |  |
| Sanguisorba officinalis L. | ROSACEAE | 1 | 1 |  |  |  |  |  |  | 1 | 1 | 1 | 1 |  |  |
| Saxifraga aizoides L. | SAXIFRAGACEAE | 1 | 1 | 1 |  | 1 | 1 | 1 | 1 | 1 | 1 | 1 | 1 |  |  |
| Saxifraga cernua L. | SAXIFRAGACEAE | 1 | 1 | 1 |  | 1 | 1 | 1 | 1 | 1 | 1 | 1 | 1 |  |  |
| Saxifraga cespitosa L. | SAXIFRAGACEAE |  | 1 | 1 |  |  | 1 | 1 | 1 | 1 | 1 |  |  |  |  |
| Saxifraga cotyledon L. | SAXIFRAGACEAE |  | 1 |  |  |  |  |  |  |  | 1 |  | 1 |  |  |
| Saxifraga hirculus L. subsp. compacta Hedberg | SAXIFRAGACEAE |  | 1 | 1 |  |  | 0 |  | 0 | 1 | 0 | 0 | 0 |  | Vicariant taxa in E Canada: subsp. hirculus & subsp. propinqua (R.Br.) Á.Löve & D.Löve; N Greenland: subsp. propinqua; N Fennoscandia, S Scandinavia & Britain: subsp. hirculus (also N Urals) |
| Saxifraga hyperborea R.Br. | SAXIFRAGACEAE | 1 |  | 1 |  |  | 1 | 1 | 1 | 1 |  |  |  |  |  |
| Saxifraga hypnoides L. | SAXIFRAGACEAE |  | 1 |  | 1 |  |  |  |  |  | 1 | 1 |  |  |  |
| Saxifraga nathorstii (Dusén) Hayek | SAXIFRAGACEAE | 1 |  |  |  |  |  |  |  |  |  |  |  | End | Allopolyploid species from S. aizoides & S. oppositifolia |
| Saxifraga oppositifolia L. | SAXIFRAGACEAE | 1 | 1 | 1 | 1 | 1 | 1 | 1 | 1 | 1 | 1 | 1 | 1 |  |  |
| Saxifraga paniculata Mill. subsp. laestadii (Neuman) Karlsson | SAXIFRAGACEAE |  | 1 |  |  |  | 1 | 1 |  |  | 1 |  | 0 |  | Present in N Norway vicariant taxon in S Scandinavia & C Europe: subsp. paniculata |
| Saxifraga platysepala (Trautv.) Tolm. | SAXIFRAGACEAE | 1 |  | 1 |  |  | 1 |  | 1 | 1 |  |  |  |  |  |
| Saxifraga rivularis L. subsp. arctolitoralis (Jurtz. & V.V.Petrovsky) M.H.Jørg. & Elven | SAXIFRAGACEAE | 1 |  |  |  |  | 1 | 1 | 1? |  |  |  |  |  |  |
| Saxifraga rivularis L. subsp. rivularis | SAXIFRAGACEAE |  | 1 | 1 | 1 |  | 1 | 1 | 1 | 1 | 1 | 1 |  |  |  |
| Saxifraga rosacea Moench | SAXIFRAGACEAE |  | 1 |  | 1 |  |  | 1? |  |  |  | 1 | 1 |  |  |
| Saxifraga svalbardensis Øvstedal | SAXIFRAGACEAE |  |  | 1 |  |  |  |  |  | 1 |  |  |  | End | Endemic to Svalbard |
| Saxifraga tricuspidata Rottb. | SAXIFRAGACEAE | 1 |  |  |  |  | 1 | 1 | 1 |  |  |  |  |  |  |
| Scorzoneroides autumnalis (L.) Moench (Leontodon autumnalis L.) | ASTERACEAE |  | 1 |  | 1 |  |  |  |  |  | 1 | 1 | 1 |  |  |
| Scrophularia nodosa L. | SCROPHULARIACEAE |  |  |  | 1 |  |  |  |  |  | 1 | 1 | 1 |  |  |
| Sedum acre L. | CRASSULACEAE | 1 | 1 |  |  |  |  |  |  |  | 1 | 1 | 1 |  |  |
| Sedum annuum L. | CRASSULACEAE |  | 1 |  |  |  |  | 1 |  |  | 1 |  | 1 |  |  |
| Sedum villosum L. | CRASSULACEAE | 1 | 1 |  | 1 |  |  | 1 |  |  | 1 | 1 | 1 |  |  |
| Selaginella selaginoides (L.) P.Beauv. ex Mart. & Schrank | SELAGINELLACEAE | 1 | 1 |  | 1 |  | 1 | 1 |  | 1 | 1 | 1 | 1 |  |  |
| Sesleria albicans Kit. | POACEAE | 1 | 1 |  |  |  |  |  |  |  |  | 1 | 1 |  |  |
| Sibbaldia procumbens L. | ROSACEAE | 1 | 1 | 1 | 1 | 1 | 1 | 1 |  | 1 | 1 | 1 | 1 |  |  |
| Sibbaldia tridentata (Sol.) Paule & Soják (Potentilla tridentata Sol.) | ROSACEAE | 1 |  |  |  |  | 1 | 1 |  |  |  |  |  |  |  |
| Silene acaulis (L.) Jacq. | CARYOPHYLLACEAE | 1 | 1 | 1 | 1 | 1 | 1 | 1 | 1 | 1 | 1 | 1 | 1 |  |  |
| Silene dioica (L.) Clairv. | CARYOPHYLLACEAE |  |  |  | 1 |  |  |  |  |  | 1 | 1 | 1 |  |  |
| Silene involucrata (Cham. & Schltdl.) Bocquet subsp. furcata (Raf.) V.V.Petrovsky & Elven | CARYOPHYLLACEAE | 1 |  | 1 |  |  | 1 | 1 | 1 | 1 | 0 |  |  |  | Vicariant taxon in N Fennoscandia: subsp. tenella (Tolm.) Bocquet. Also subsp. tenella in N Urals |
| Silene sorensenis (B.Boivin) Bocquet | CARYOPHYLLACEAE | 1 |  |  |  |  | 1 | 1 | 1 |  |  |  |  |  |  |
| Silene uniflora Roth | CARYOPHYLLACEAE |  | 1 |  |  |  |  |  |  |  | 1 | 1 |  |  |  |
| Silene uralensis (Rupr.) Bocquet subsp. arctica (Th.Fr.) Bocquet | CARYOPHYLLACEAE | 1 |  | 1 |  |  | 1 | 1 | 1 | 1 | 0 |  |  |  | Vicariant taxa in Fennoscandia: S. wahlbergella Chowdhuri; in N Urals in addition the vicariant S. uralensis subsp. uralensis |
| Sorbus aucuparia L. | ROSACEAE | 1 | 1 |  |  |  | 0 | 0 |  | 1 | 1 | 1 | 1 |  | Vicariant taxon in E Canada & Greenland: S. groenlandica (C.K.Schneid.) Á.Löve & D.Löve |
| Sparganium angustifolium Michx. | SPARGANIACEAE | 1 | 1 |  | 1 |  | 1 | 1 |  |  | 1 | 1 | 1 |  |  |
| Sparganium hyperboreum Laest. ex Beurl. | SPARGANIACEAE |  | 1 |  |  |  | 1 | 1 |  | 1 | 1 |  |  |  |  |
| Spergularia salina J.Presl & C.Presl (S. marina (L.) Besser) | CARYOPHYLLACEAE |  | 1 |  |  |  | 0 | 0 |  |  | 1 | 1 |  |  | Vicariant taxon in E Canada & W Greenland: S. canadensis (Pers.) G.Don |
| Stellaria borealis Bigelow | CARYOPHYLLACEAE |  | 1 |  |  |  | 1 | 1 |  | 1 | 1 |  |  |  |  |
| Stellaria crassifolia Ehrh. | CARYOPHYLLACEAE |  | 1 |  |  |  | 1 |  |  | 1 | 1 |  |  |  |  |
| Stellaria humifusa Rottb. | CARYOPHYLLACEAE |  | 1 | 1 |  |  | 1 | 1 | 1 | 1 | 1 |  |  |  |  |
| Stellaria longipes Goldie s.l. | CARYOPHYLLACEAE | 1 |  | 1 |  |  | 1 | 1 | 1 | 1 | 1 |  |  |  |  |
| Stellaria media (L.) Vill. | CARYOPHYLLACEAE |  | 1 |  | 1 |  |  |  |  |  | 1 | 1 | 1 |  |  |
| Stellaria uliginosa Murray (S. alsine Grimm) | CARYOPHYLLACEAE |  |  |  | 1 |  | 1 |  |  |  | 1 | 1 | 1 |  |  |
| Stuckenia filiformis (Pers.) Börner subsp. borealis (Raf.) Tzvelev & Elven | POTAMOGETONACEAE | 1 |  |  |  |  | 1 | 1 |  |  |  |  |  |  |  |
| Stuckenia filiformis (Pers.) Börner subsp. filiformis | POTAMOGETONACEAE |  | 1 |  | 1 |  |  |  |  |  | 1 | 1 | 1 |  |  |
| Subularia aquatica L. subsp. americana G.A.Mulligan & Calder | BRASSICACEAE | 1 |  |  |  |  | 1 | 1 |  |  |  |  |  |  |  |
| Subularia aquatica L. subsp. aquatica | BRASSICACEAE |  | 1 |  | 1? |  |  |  |  |  | 1 | 1 |  |  |  |
| Succisa pratensis Moench | DIPSACACEAE |  | 1 |  | 1 |  |  |  |  |  | 1 | 1 | 1 |  |  |
| Taraxacum sect. Arctica | ASTERACEAE | 1 |  | 1 |  |  | 1 | 1 | 1 |  |  |  |  |  | Taraxacum arcticum (Trautv.) Dahlst. in N & E Greenland & Svalbard; T. holmenianum Sahlin in E Canada & N & E Greenland; T. phymatocarpum J.Vahl in E Canada & W, N & E Greenland; T. porsildii (Jurtz. ined) in E Canada & N & E Greenland |
| Taraxacum sect. Borea | ASTERACEAE |  | 1 |  |  |  |  |  |  |  | 1 |  |  |  |  |
| Taraxacum sect. Borealia | ASTERACEAE | 1 |  | 1 |  |  | 1? | 1 | 1 |  | 1 |  |  |  |  |
| Taraxacum sect. Erythrosperma | ASTERACEAE | 1 | 1 |  |  |  |  |  |  |  | 1 | 1 | 1 |  |  |
| Taraxacum sect. Macrodonta | ASTERACEAE |  | 1 |  | 1 | 1 |  | 1 |  |  | 1 |  |  |  |  |
| Taraxacum sect. Naevosa | ASTERACEAE |  | 1 |  | 1 |  |  | 1 |  |  | 1 | 1 |  |  |  |
| Taraxacum sect. Ruderalia | ASTERACEAE |  | 1 |  | 1 |  |  |  |  |  |  | 1 | 1 |  |  |
| Taraxacum sect. Spectabilia | ASTERACEAE |  | 1 |  | 1 |  | 1 |  |  | 1 | 1 | 1 |  |  |  |
| Taraxacum sect. Taraxacum (sect. Crocea) | ASTERACEAE |  | 1 | 1 | 1 |  | 1 | 1 |  |  | 1 | 1 |  |  |  |
| Thalictrum alpinum L. | RANUNCULACEAE | 1 | 1 |  | 1 |  | 1 | 1 |  | 1 | 1 | 1 | 1 |  |  |
| Thymus praecox subsp. britannicus (Ronniger) Holub (subsp. arcticus (Durand) Jalas) | LAMIACEAE |  | 1 |  | 1 |  |  | 1 |  |  | 1 | 1 |  |  |  |
| Tofieldia coccinea Richardson | TOFIELDIACEAE | 1 |  |  |  |  | 1 | 1 | 1 |  |  |  |  |  |  |
| Tofieldia pusilla (Michx.) Pers. | TOFIELDIACEAE | 1 | 1 | 1 | 1 | 1 | 1 | 1 |  | 1 | 1 | 1 | 1 |  |  |
| Tractema verna (Huds.) Speta (Scilla verna Huds.) | HYACINTHACEAE |  |  |  | 1 |  |  |  |  |  | 1 | 1 |  |  |  |
| Trichophorum cespitosum (L.) Hartm. subsp. cespitosum | CYPERACEAE | 1 | 1 |  |  | 1 | 1 | 1 |  | 1 | 1 | 1 | 1 |  |  |
| Trichophorum cespitosum (L.) Hartm. subsp. germanicum (Palla) Hegi | CYPERACEAE |  |  |  | 1 |  |  |  |  |  | 1 | 1 |  |  |  |
| Triglochin maritima L. | JUNCAGINACEAE |  | 1 |  | 1 |  | 1 |  |  |  | 1 | 1 |  |  |  |
| Triglochin palustris L. | JUNCAGINACEAE | 1 | 1 |  | 1 |  | 1 | 1 |  | 1 | 1 | 1 | 1 |  |  |
| Tripleurospermum maritimum (L.) W.D.J.Koch subsp. maritimum | ASTERACEAE |  | 1 |  | 1? |  |  |  |  |  | 1 | 1 |  |  |  |
| Tripleurospermum maritimum (L.) W.D.J.Koch subsp. phaeocephalum (Rupr.) Hämet-Ahti | ASTERACEAE |  | 1 |  | 1? |  | 1 | 1 |  | 1 | 1 |  |  |  |  |
| Tripleurospermum maritimum (L.) W.D.J.Koch subsp. subpolare (Pobed.) Hämet-Ahti | ASTERACEAE | 1? | 1 |  | 1? |  |  |  |  | 1 | 1 | 1? |  |  |  |
| Trisetum molle (Michx.) Kunth | POACEAE |  | 1 |  |  |  | 1 | 1 |  |  |  |  |  |  |  |
| Trisetum spicatum (L.) K.Richt. | POACEAE | 1 | 1 | 1 |  |  | 1 | 1 | 1 | 1 | 1 |  | 1 |  |  |
| Utricularia minor L. | LENTIBULARIACEAE | 1 | 1 |  |  |  | 1 | 1 |  |  | 1 | 1 | 1 |  |  |
| Utricularia vulgaris L. subsp. vulgaris | LENTIBULARIACEAE |  |  |  | 1 |  | 0 |  |  |  | 1 | 1 | 1 |  | Vicariant taxon in E Canada (across North America): subsp. macrorhiza (Leconte) R.T.Clausen |
| Vaccinium myrtillus L. | ERICACEAE |  | 1 |  | 1 |  |  | 1 |  |  | 1 | 1 | 1 |  |  |
| Vaccinium uliginosum L. | ERICACEAE | 1 | 1 | 1 | 1 | 1 | 1 | 1 | 1 | 1 | 1 | 1 | 1 |  |  |
| Vaccinium vitis-idaea L. | ERICACEAE | 1 | 1 |  | 1 |  | 1 | 1 | 1 | 1 | 1 | 1 | 1 |  |  |
| Vahlodea atropurpurea (Wahlenb.) Fr. ex Hartm. | POACEAE | 1 |  |  |  |  | 1 | 1 |  |  | 1 |  |  |  |  |
| Valeriana sambucifolia J.Mikan | VALERIANACEAE | 1 | 1 |  |  |  |  |  |  |  | 1 | 1 | 1 |  |  |
| Veronica alpina L. subsp. alpina | PLANTAGINACEAE |  | 1 |  | 1 |  | 1 | 1 |  | 1 | 1 | 1 |  |  |  |
| Veronica alpina L. subsp. pumila (All.) Pennell | PLANTAGINACEAE |  | 1 |  |  |  |  | 1 |  |  | 1 |  | 1 |  |  |
| Veronica anagallis-aquatica L. | PLANTAGINACEAE | 1 | 1 |  |  |  |  |  |  |  | 1 | 1 | 1 |  |  |
| Veronica arvensis L. | PLANTAGINACEAE |  |  |  | 1 |  |  |  |  |  | 1 | 1 | 1 |  |  |
| Veronica fruticans Jacq. | PLANTAGINACEAE | 1? | 1 |  | 1 |  |  | 1 |  |  | 1 | 1 | 1 |  |  |
| Veronica officinalis L. | PLANTAGINACEAE |  | 1 |  | 1 |  |  |  |  |  | 1 | 1 | 1 |  |  |
| Veronica scutellata L. | PLANTAGINACEAE | 1 | 1 |  |  |  | 1 |  |  |  | 1 | 1 | 1 |  |  |
| Veronica serpyllifolia L. subsp. serpyllifolia | PLANTAGINACEAE |  | 1 |  | 1 |  |  |  |  |  | 1 | 1 | 1 |  |  |
| Veronica wormskjoldii Roem. & Schult. | PLANTAGINACEAE | 1? |  |  |  |  | 1 | 1 |  |  |  |  |  |  |  |
| Viola adunca Sm. | VIOLACEAE | 1 |  |  |  |  | 1 | 1 |  |  |  |  |  |  |  |
| Viola canina L. | VIOLACEAE |  | 1 |  | 1 |  |  | 1 |  |  | 1 | 1 | 1 |  |  |
| Viola epipsila Ledeb. | VIOLACEAE |  | 1 |  |  |  |  |  |  | 1 | 1 |  |  |  |  |
| Viola palustris L. | VIOLACEAE | 1 | 1 |  | 1 |  | 1 | 1 |  | 1 | 1 | 1 | 1 |  |  |
| Viola riviniana Rchb. | VIOLACEAE |  | 1 |  | 1 |  |  |  |  |  | 1 | 1 | 1 |  |  |
| Viola tricolor L. | VIOLACEAE |  | 1 |  | 1 |  |  |  |  |  | 1 | 1 | 1 |  |  |
| Viscaria alpina (L.) G.Don | CARYOPHYLLACEAE | 1 | 1 |  |  |  | 1 | 1 |  |  | 1 | 1 | 1 |  |  |
| Woodsia alpina (Bolton) Gray | WOODSIACEAE | 1 | 1 |  |  | 1 | 1 | 1 | 1 | 1 | 1 | 1 | 1 |  |  |
| Woodsia glabella R.Br. ex Richardson | WOODSIACEAE | 1 |  | 1 |  |  | 1 | 1 | 1 | 1 | 1 |  |  |  |  |
| Woodsia ilvensis (L.) R.Br. | WOODSIACEAE | 1 | 1 |  |  | 1 | 1 | 1 |  | 1 | 1 | 1 | 1 |  |  |
| Zannichellia palustris L. subsp. palustris | POTAMOGETONACEAE | 1 | 1 |  |  |  | 1 |  |  |  | 1 | 1 | 1 |  |  |
| Zostera angustifolia (Hornem.) Rchb. | ZOSTERACEAE |  | 1 |  |  |  | 1 | 1 |  |  | 1 | 1 |  |  |  |
| Zostera marina L. | ZOSTERACEAE |  |  |  | 1 |  | 1 | 1 |  |  | 1 | 1 |  |  |  |

**Table S4.** Model selection using Akaike’s information criterion corrected for small sample size (AICc) for proportion of regional diversity in recipient island relative to main source region (n = 46) depending on the log of the size of the recipient island in km^2^ (*island size*), the mode of pollination (*pollination*: insect, wind), the presence of dispersal adaptations (*dispersal*: long-distance, short-distance), and dispersal distance (*distance*: km). Models with ΔAICc <2 were considered equally adequate and are highlighted in bold.

| Model | K | ΔAICc |
| --- | --- | --- |
| Constant | 3 | 12.18 |
| Island size | 4 | 2.07 |
| Pollination | 4 | 10.53 |
| Dispersal | 4 | 14.25 |
| Distance | 4 | 13.93 |
| **Island size + Pollination** | **5** | **1.05** |
| Island size + Dispersal | 5 | 4.58 |
| Island size + Distance | 5 | 2.09 |
| Pollination + Dispersal | 5 | 13.04 |
| Pollination + Distance | 5 | 12.47 |
| Dispersal + Distance | 5 | 15.89 |
| **Island size * Pollination** | **6** | **0** |
| Island size * Dispersal | 6 | 7.14 |
| **Island size * Distance** | **6** | **0.68** |
| Pollination * Dispersal | 6 | 14.82 |
| Pollination * Distance | 6 | 15.04 |
| Dispersal * Distance | 6 | 18.49 |

**LITERATURE CITED**

Alsos IG, Eidesen PB, Ehrich D*, et al.* 2007. Frequent long-distance colonization in the changing Arctic. *Science* 316: 1606-1609.

Alsos IG, Alm T, Normand S, Brochmann C. 2009. Past and future range shift and loss of genetic diversity in dwarf willow (*Salix herbacea* L.) inferred from genetics, fossils, and modelling. *Global Ecology and Biogeography* 18: 223-239.

Alsos IG, Ehrich D, Thuiller W*, et al.* 2012. Genetic consequences of climate change for northern plants. *Proceedings of the Royal Society of London Series B* 279: 2042-2051.

Corander J, Marttinen P. 2005. BAPS: Bayesian Analysis of Population Structure. *Manual v. 3.1*.

Ehrich D, Gaudeul M, Assefa A*, et al.* 2007. Genetic consequences of Pleistocene range shifts: contrast between the Arctic, the Alps and the East African mountains. *Molecular Ecology* 16: 2542-2559.

Eidesen P, Carlsen T, Molau U, Brochmann C. 2007a. Repeatedly out of Beringia: *Cassiope tetragona* embraces the Arctic. *Journal of Biogeography* 34: 1559-1574.

Eidesen PB. 2007. *Arctic-alpine plants on the move: Individual and comparative phylogeographies reveal responses to climate change.* PhD PhD Thesis, Natural History Museum, Oslo.

Eidesen PB, Alsos IG, Popp M, Stensrud Ø, Suda J, Brochmann C. 2007b. Nuclear versus plastid data: complex Pleistocene history of a circumpolar key species. *Molecular Ecology* 16: 3902-3925.

Eidesen PB, Ehrich D, Bakkestuen V*, et al.* 2013. Genetic roadmap of the Arctic: plant dispersal highways, traffic barriers and capitals of diversity. *New Phytologist* 200: 898-910.

Elven R. 2005. *J. Lid & D.T. Lid. Norsk flora. 7th edition,* Oslo, Det Norske Samlaget.

Elven R, Murray DF, Razzhivin VY, Yurtsev BA. 2011. Annotated checklist of the Panarctic Flora (PAF). Vascular plants. <http://nhm2.uio.no/paf/>.

Evanno G, Regnaut S, Goudet J. 2005. Detecting the number of clusters of individuals using the software structure: a simulation study. *Molecular Ecology* 14: 2611-2620.

Falush D, Stephens M, Pritchard JK. 2007. Inference of population structure using multilocus genotype data: dominant markers and null alleles. *Molecular Ecology Notes* 7: 574-578.

Hansen K. 1966. Vascular plants in the Faeroes. Horizontal and vertical distribution. *Dansk Botanisk Arkiv* 24: 141.

Hubisz MJ, Falush D, Stephens M, Pritchard JK. 2009. Inferring weak population structure with the assistance of sample group information. *Molecular Ecology Resources* 9: 1322-1332.

Hultén E, Fries M. 1986. *Atlas of North European vascular plants north of the Tropic of Cancer,* Königstein, Koeltz Scientific Books.

Jørgensen MH, Elven R, Tribsch A, Gabrielsen TM, Stedje B, Brochmann C. 2006. Taxonomy and evolutionary relationships in the *Saxifraga rivularis* complex. *Systematic Botany* 31: 702-729.

Kristinsson H. 2008. Íslensk plöntutal, blómplöntur og byrkingar. *Fjölrit Náttúrufrædistofnunar* 51: 58.

Lid J. 1964. The flora of Jan Mayen. *Norsk Polarinstitutt Skrifter* 130: 1-109.

Mossberg B, Stenberg L. 2007. *Gyldendals store Nordiske flora. Revidert og utvidet utgave,* Oslo, Gyldendal Norsk Forlag.

Pritchard JK, Stephens M, Donnelly PJ. 2000. Inference of population structure using multilocus genotype data. *Genetics* 155: 945-959.

R_Core_Team. 2013. R: A language and environment for statistical computing Foundation for Statistical Computing, Vienna, Austria.

Rohlf F. 1990. NTSYS-PC. Numerical taxonomy and multivariate analysis system. Version 2.02 ed. Setauket, NY, Exeter Software.

Rosenberg NA, Pritchard JK, Weber JL*, et al.* 2002. Genetic structure of human populations. *Science* 298: 2381-2385.

Schönswetter P, Paun O, Tribsch A, Niklfeld H. 2003. Out of the Alps: colonization of Northern Europe by East Alpine populations of the Glacier Buttercup *Ranunculus glacialis* L. (Ranunculaceae). *Molecular Ecology* 12: 3373-3381.

Schönswetter P, Elven R, Brochmann C. 2008. Trans-Atlantic dispersal and large-scale lack of genetic structure in the circumpolar, arctic-alpine sedge *Carex bigelowii* s. l. (Cyperaceae). *Am. J. Bot.* 95: 1006-1014.

Skrede I, Eidesen PB, Portela RP, Brochmann C. 2006. Refugia, differentiation and postglacial migration in arctic-alpine Eurasia, exemplified by the mountain avens (*Dryas octopetala* L.). *Molecular Ecology* 15: 1827-1840.

Stace CA. 1997. *New Flora of the British Isles,* Cambridge, Cambridge University Press.

Van de Peer Y, De Wachter Y. 1994. TREECON for Windows: a software package for the construction and drawing of evolutionary trees for the Microsoft Windows environment. *Computer Applications in the Bioscience* 10: 569-570.

Westergaard K, Alsos I, Engelskjøn T, Flatberg K, Brochmann C. 2011a. Trans-Atlantic genetic uniformity in the rare snowbed sedge *Carex rufina*. *Conservation Genetics* 12: 1367-1371.

Westergaard KB, Jørgensen MH, Gabrielsen TM, Alsos IG, Brochmann C. 2010. The extreme Beringian/Atlantic disjunction in *Saxifraga rivularis* (Saxifragaceae) has formed at least twice. *Journal of Biogeography* 37: 1262-1276.

Westergaard KB, Alsos IG, Popp M, Egelskjøn T, Flatberg KI, Brochmann C. 2011b. Glacial survival may matter after all: nunatak signatures in the rare European populations of two west-arctic species. *Molecular Ecology* 20: 376-393.

Whitlock R, Hipperson H, Mannarelli M, Butlin RK, Burke T. 2008. An objective, rapid and reproducible method for scoring AFLP peak-height data that minimizes genotyping error. *Molecular Ecology Resources* 8: 725-735.

Ziegenhagen B, Guillemaut P, Scholz F. 1993. A procedure for mini-preparations of genomic DNA from needles of silver fir (*Abies alba* mill.). *Plant Molecular Biology Reporter* 11: 117-121.
